# Molecular Evolution of Peptide Ligands with Custom-Tailored Characteristics for Targeting of Glycostructures

**DOI:** 10.1371/journal.pcbi.1002800

**Published:** 2012-12-13

**Authors:** Niels Röckendorf, Markus Borschbach, Andreas Frey

**Affiliations:** 1 Division of Mucosal Immunology & Diagnostics, Priority Program Asthma & Allergy, Research Center Borstel, Airway Research Center North (ARCN), Member of the German Center for Lung Research (DZL), Borstel, Germany; 2 FHDW, University of Applied Sciences, Bergisch Gladbach, Germany; Bar Ilan University, Israel

## Abstract

As an advanced approach to identify suitable targeting molecules required for various diagnostic and therapeutic interventions, we developed a procedure to devise peptides with customizable features by an iterative computer-assisted optimization strategy. An evolutionary algorithm was utilized to breed peptides *in silico* and the “fitness” of peptides was determined in an appropriate laboratory *in vitro* assay. The influence of different evolutional parameters and mechanisms such as mutation rate, crossover probability, gaussian variation and fitness value scaling on the course of this artificial evolutional process was investigated. As a proof of concept peptidic ligands for a model target molecule, the cell surface glycolipid ganglioside G_M1_, were identified. Consensus sequences describing local fitness optima were reached from diverse sets of L- and proteolytically stable D lead peptides. Ten rounds of evolutional optimization encompassing a total of just 4400 peptides lead to an increase in affinity of the peptides towards fluorescently labeled ganglioside G_M1_ by a factor of 100 for L- and 400 for D-peptides.

## Author Summary

A clever identification procedure is crucial when peptidic ligands for diagnostic and therapeutic techniques such as *in vivo* imaging or drug targeting are to be developed. Here, we present a propitious and versatile approach for the discovery of peptide sequences with custom features that is based on an iterative computer-assisted optimization process. The methodology smartly combines *in silico* evolution with *in vitro* testing to quickly obtain promising peptide ligand candidates with desired properties. To validate our method in a proof of concept we tried to identify peptide sequences that can bind to a glycosidic cell membrane component. We applied the evolution process by starting out with a small population of peptide lead sequences and achieved a constant increase in affinity between the peptide candidates and their target molecule with each generation. After 10 rounds and a total number of only 4400 peptides synthesized and tested, a more than 100fold improvement in target recognition could be achieved. Since all kinds of building blocks useable in chemical solid phase peptide synthesis can in principle be employed in this evolutionary optimization process, our method should prove a most versatile approach for the optimization of peptides, peptoids and peptomers towards a preset functionality.

## Introduction

In the field of bioactive substances, peptides are drawing increasing attention as they close the gap between small molecules and proteins, combining the compact size and synthetic accessibility of the former with the high specificity in molecular recognition processes of the latter. Of particular interest in this context are tasks where targeting of an active compound to a defined cellular or molecular structure is desired, e.g. the site-specific delivery of drugs, vaccines, or contrast agents for molecular imaging applications [1], [2].

To date, mainly antibodies are used in such situations [2], [3], yet the large size of an antibody ligand severely hampers tissue penetration and optical resolution, and its antigenicity and degradability limit its use *in vivo*. Hence, various approaches to artificially reduce ligand size while maintaining specificity are being pursued to establish the next generation of targeting molecules [4]. Small peptides built up of 10 to 20 amino acid residues which permit highly specific interactions with biological targets carry this concept to its final consequence [5], [6]. Although the use of peptides in therapy and diagnostics may be hampered by their proteolytic lability or limited cell penetration, too, these obstacles can be overcome by building up proteolytically stable peptide isomers from D-amino acid residues or by coupling the peptides to membrane shuttles [7], [8]. Far more challenging is the identification of peptide sequences that exhibit the necessary sensitivity and specificity of a targeting ligand. To date, high throughput screening of large peptide libraries is a common approach for the identification of peptide ligands, but with increasing ligand length the procedure rapidly reaches its limits. Beyond a length of 9–10 amino acids such libraries are no longer representative due to the exponentially growing peptide sequence space (e.g. 10^21^ sequences for 16mer L-peptides).

In order to overcome this limitation, computational structure based design methods suitable for reduction of the sequence space allocatable have been established. If the 3D molecular structure of the target is available it can be used in docking approaches for the design of peptide ligands for these targets using mere *in silico* procedures [9], [10]. Another way to optimize peptide sequences for desired applications is the use of structural scaffolds [10] in molecular dynamics simulations. Both approaches work best with rigid proteinacious target molecules.

Structure-independent design of peptides can be accomplished by e. g. sequence motif scanning [10] utilizing learning algorithms such as artificial neural networks. This technique, however, is limited to sequence data already present in training sets and often fails to create novelty.

In protein design, directed evolution strategies which aim to improve candidates by iterative rounds of mutations and functional screenings constitute another way to optimize biomolecules [11]. These methods, which include gene-shuffling, site-directed mutagenesis and chimeragenesis, work on the DNA-level and hence are restricted to gene encoded optimization candidates. Therefore the incorporation of non-natural building blocks or the optimization of all D-peptides cannot be achieved with these techniques. Yet, the inclusion of a function-screening step in such directed evolution strategies represents a definite strength. In light of the above, it appears most reasonable to employ not a structure, but a function-driven strategy for the identification of peptides suitable for the desired applications [12], [13], [14].

We have devised such a strategy based on a molecular optimization process that mimics Darwinian evolution. The evolutionary process is initiated with a peptide library of random sequences or with lead peptides either of known rudimentary suitability or designed by structural considerations. The functional prowess of each peptide is assessed in an appropriate biological assay, in result of which all individuals are assigned “fitness values”. The resulting peptide population is operator-inspected and top candidates are selected to act as parent peptides for the follow up generation. In a computational step, an evolutionary algorithm (EA) is used by which the selected peptides are propagated *in silico* via crossing and mutating them, with the “fittest” candidates having the highest probability of passing on their “genetic information”, i.e. their peptide sequence, to produce a filial generation. We have applied this cooperative *in silico* and *in vitro* optimization methodology to identify peptidic ligands for the cell membrane glycolipid ganglioside G_M1_, a potential target *e. g.* for diagnostic imaging applications at the mucosal wall or for mucosal vaccine delivery systems [15].

## Results/Discussion

Evolutionary optimization of peptidic ligands is a complex process where numerous parameters and different evolutionary mechanisms may depend on and influence each other. In order to keep those variables at a manageable level a general framework was defined in the beginning: i) the length of the ligand to be evolved was set to 16 amino acids, which was deemed a good compromise between synthetic accessibility and sequence space; ii) a single most relevant criterion – optimal binding to the desired target – was selected as evolutionary goal and iii) appropriate parameter settings and combinations of evolutionary mechanisms were selected on the basis of empirical *in silico* simulation studies. The latter was done by shaping 16mer peptides towards a defined characteristic (molecular mass) as “pseudo-fitness”. The fitness values were optimized in distance metric simulations, and the evolutionary optimization data were evaluated in order to identify settings which lead the algorithm to converge in a minimal generation count.

As evolutionary goal we decided to optimize a peptide ligand for binding to ganglioside G_M1_. This particular target molecule was chosen for several reasons. Firstly, carbohydrate molecules, e. g. on cell surface receptors, are a highly relevant class of biological targets, but they are demanding candidates in computational design due to their dynamic solution structure and microheterogeneity [16]. We reasoned that our structure-independent, function-driven approach should be particularly suited to identify ligands for such targets. Secondly, G_M1_ is a small target, its molecular mass of 1.6 kDa lies within the same range as that of a putative peptide ligand. Therefore its binding sites for different leads should largely overlap which heightens the probability for cross-bred offspring to also bind in this region. Proteins and other large target molecules, on the other hand, may offer multiple, independent binding sites for which leads can be identified. Such leads will not produce meaningful progeny upon crossing and thus would unreasonably discredit our approach. And lastly, G_M1_ is an attractive candidate from the biomedical point of view because it has already been singled out as potential target molecule [15]. Moreover, we already had identified a battery of structurally diverse alleged G_M1_ binders which could serve as lead sequences for the optimization process [17] and set up an *in vitro* assay which allowed the simultaneous investigation of a large number of ligand-target interactions.

For this assay, all peptides are synthesized in arrays on cellulose membranes. These peptide libraries are probed in a dot-blot type biochemical binding procedure by incubating them with a fluorophore-labeled ganglioside G_M1_ derivative (lysoG_M1_/DY650) [17], and the fluorescence intensities of the individual peptide spots after excitation are quantitated.

As initial population for the evolution process the previously identified 64 lead peptide sequences were used [17]. To enable the parallel identification of proteolytically stable D-peptide ligands corresponding retro-inverso D-peptides were submitted to the same process in parallel. The L- and D-peptides were analyzed for their capacity to bind to the G_M1_ probe, and ranked according to the fluorescence intensity of their respective spots (Table S1). Peptides yielding fluorescence signals above a statistically defined background [18] were manually selected as lead sequences for the subsequent evolutionary optimization processes (Table 1). In these lead peptides, the potential influence of individual amino acids on G_M1_-probe binding was estimated by an “alanine walk” experiment; arginine, phenylalanine, tryptophan and histidine were revealed to be critical for binding.

**Table 1:** Lead peptides for the molecular evolution process identified from a panel of alleged G_M1_-binding peptides.

No.	Lead sequences L	Fitness	Lead sequences D	Fitness
1	LPRHRDTGILDSIGRF	34,68	apqrlqwfagplrrfd	157,50
2	DFRRLPGAFWQLRQPA	31,36	tnhyekifsyteslag	102,36
3	PQIAMFCGRLNMNMNV	27,93	drhrplfghrahdmts	90,77
4	GALSETYSFIKEYHNT	26,06	plfghrahdmtsatal	87,02
5	YEVNWKTHEIKVKGQN	25,81	gtdrhrplfghrahdm	82,55
6	AEPQIAMFCGRLNMHM	25,12	ligtdrhrplfghrah	77,87
7	HHCSILKEVWHVKKLG	24,71	fikdnlthiqtnhyea	75,51
8	GWWYKGRARPVSAVAA	24,04	hrplfghrahdmtsat	64,35
9	AKVEKLCVWNNKTPHA	22,65	frgisdligtdrhrpl	59,27
10	ASDWYDEMLTWNIHGA	22,32	mhmnlrgcfmaiqpea	53,31
11	VWRLLAPPFSNRLLPA	21,46	apllrnsfppallrwv	52,06
12			vnmhmnlrgcfmaiqp	51,71
13			lrgcfmaiqpeallga	50,05

Fitness values correspond to fluorescence intensities obtained in a solid-phase binding assay with lysoG_M1_/DY650-conjugate (arbitrary units) and reflect the probe-binding strength of each peptide. Highlighted amino acids were identified in “alanine walk” experiments as beneficial (boldface) or detrimental (underlined) for binding.

**Figure 1:**
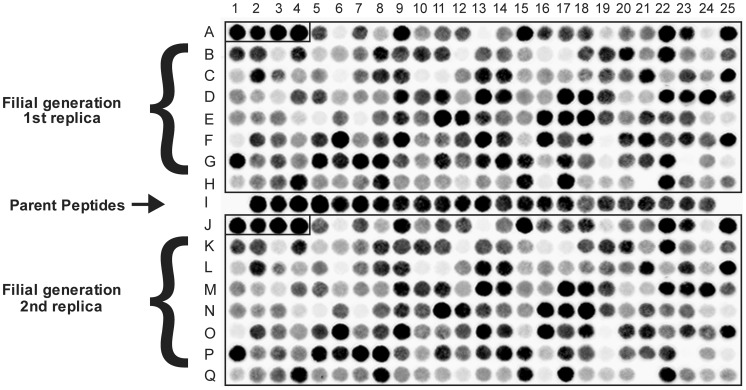
Biochemical assay to determine candidate peptide fitness. SPOT Libraries of peptides were synthesized on cellulose membrane supports and screened for ganglioside binding with fluorophore-labeled lysoG_M1_. Rows A–H and J–Q contain two arrays of the same filial generation of 200 candidate peptides, row I contains the parent peptides used for the creation of this generation, providing an internal reference for the improvement of fitness. The four best lead peptides are again included in each array of the filial generation (spots A1–A4 and J1–J4).

To test the probe binding capacity of the peptide candidates in each generation of the evolutionary process, the peptide sequences proposed by the evolutionary algorithm were synthesized in duplicate onto cellulose membranes and probed with lysoG_M1_/DY650. In each array, the “fittest” peptides of the corresponding parent generation were included as a reference to monitor the progress of the optimization process (Figure 1).

**Figure 2:**
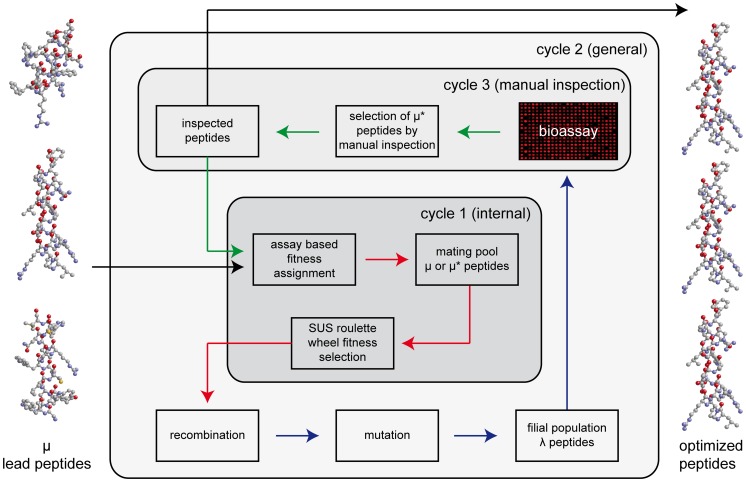
Diagram of the evolutionary optimization process. The process is divided into three cycles, an internal, genetic algorithm (GA)-like cycle 1, a general, evolutionary strategy (ES)-like cycle 2, and a bioassay-based cycle 3 which includes the selection of peptides by manual inspection. Internal cycle 1: fitness assignment to lead or parent peptides, generation of a mating pool of μ or μ* peptides, nonlinear fitness scaling of all the assigned values and afterwards λ-times stochastic universal sampling (SUS) by a roulette wheel function of the peptides from the mating pool for recombination. General cycle 2: λ-times recombination and subsequent mutation with specific gaussian variation of the recombination and mutation setup thereby creating a filial population of λ peptides. Cycle 3: biochemical testing and manual inspection of the resulting population in the bioassay and manual selection of μ* peptides out of λ peptides in the filial population to act as new parent peptides in the next generation.

The evolutionary algorithm (EA) that was used for the mating of the peptide sequences was a generic population-based heuristic optimization algorithm designed for the java runtime environment (Figure 2 and Supplementary Figure S1).

Our evolutionary process differs from the standard genetic algorithm (GA) and evolutionary strategy (ES) onset [19] as well as from state of the art directed evolution of custom tailored characteristics [20], [21], [22] in several points. The work flow in the software is divided into two modules, one module is in charge of the general, ES-like cycle 2 and a second one for the internal, GA-like cycle 1. A third, external cycle handles the determination of fitness values for all individuals of a population by the biochemical assay. At the beginning of cycle 1, the fitness values assigned to the peptides are scaled by a fitness scaling function, and fitness proportional selection by stochastic universal sampling (SUS) of parent peptides is performed to create a mating pool of peptide sequences. Sequences from that pool enter cycle 2 and are first 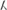-times recombined, and the resulting peptides are then mutated to establish a finished filial generation of λ peptides. The peptide sequences created this way are synthesized in parallel for cycle 3, where their fitness is determined in the biochemical assay. The results are manually inspected to select μ* candidates to act as parent peptides for the next generation. This process is repeated until optimized peptides that meet predefined criteria or display a consensus motif (see below) are obtained (see Supplementary Figure S1 for a more detailed description of the algorithm).

The fitness values that quantify the suitability of a peptide sequence were deduced from the fluorescence intensities of the respective peptide spot in the *in vitro* G_M1_-binding assay. The probability of each candidate sequence to participate in recombination events was determined by subjecting the fitness values to a fitness scaling function. Populations of filial peptides were created by applying the “evolutional parameters” - crossover rate, number of fracture sites and mutation rate - to the lead peptides. In contrast to the situation in biological systems, in our artificial setup these essential EA-parameter settings which strongly influence the performance of the optimization progress can be configured freely [23]. As no theoretical model for the global optimization of parameter settings in such algorithms is available to date [24], we had to work out appropriate settings for our problem. As a starting point, the crossover rate was adjusted to 100%, i.e. all sequences underwent recombination, the number of fracture sites was set to 1 and the mutation rate to 7% [23], i.e. an average of one sequence position in each 16mer peptide was mutated. The probability distribution onset for recombination and mutation were equal for all positions in the peptide sequences [23]. These initial parameter settings had been determined in empirical simulation experiments using distance metric calculations.

Concerning the population size which also is an important parameter in the optimization procedure we had to consider that the number of peptides synthesized in each generation ought to be large enough to provide sufficient sequence variability for a successful evolution progress. We chose a population size of 200 peptides in each generation as this could be easily managed in form of a SPOT-synthesized peptide array with replicas. The influence of changes in the parameter settings on the results of the optimization process was investigated in the first generations of L-peptide evolution. To begin with, the scaling function which is applied to the fitness values of the peptides in order to increase the differences between these values before starting the mating process was varied. From the lead peptides, two populations of filial peptides were generated, one resulting from a square, the other from an exponential scaling function. By using the square fitness scaling function, candidate sequences with a high fitness value are more often selected for reproduction than it would be the case without or with e. g. a linear scaling function. The exponential fitness scaling function magnifies this effect and puts even more emphasis on only the very fittest candidates, strongly reducing the sequence space available in the filial generation. In our set up, exponential fitness scaling led to a premature decrease in sequence diversity and was therefore deemed inappropriate for a successful optimization and not pursued further.

Manual population inspection as the driving force of a directed selection process (cycle 3) was embedded into the flow of the evolutionary algorithm (Figure 2) thereby introducing research experience into each optimization cycle. This knowledge was utilized to choose the number of succeeding peptides out of a population that shall - according to their fitness - serve as “parents” for the next generation. The influence of larger and smaller populations of such parent peptide sequences on the optimization process was investigated using sequence data from the second generation (gen2). We compared a choice of the 20 fittest (“very fit”) versus the 32 fittest peptides (“very fit+fit”) from gen2 as parent sequences to generate two populations of the third generation (gen3). While we observed that the increase in average fitness of the 22 top candidates of the filial generation was higher with only 20 “very fit” parents (1.8 fold) than with 32 “very fit+fit” parents (1.5 fold), the total number of peptides with high fitness values was lower in the filial population derived from the smaller parent population than in the filial population derived from the larger parent population. We therefore chose a compromise between high increase in average fitness and high number of very fit filial peptides and decided on using the 25 fittest peptides of each generation as parents for the next evolution round from generation 4 onwards.

**Figure 3:**
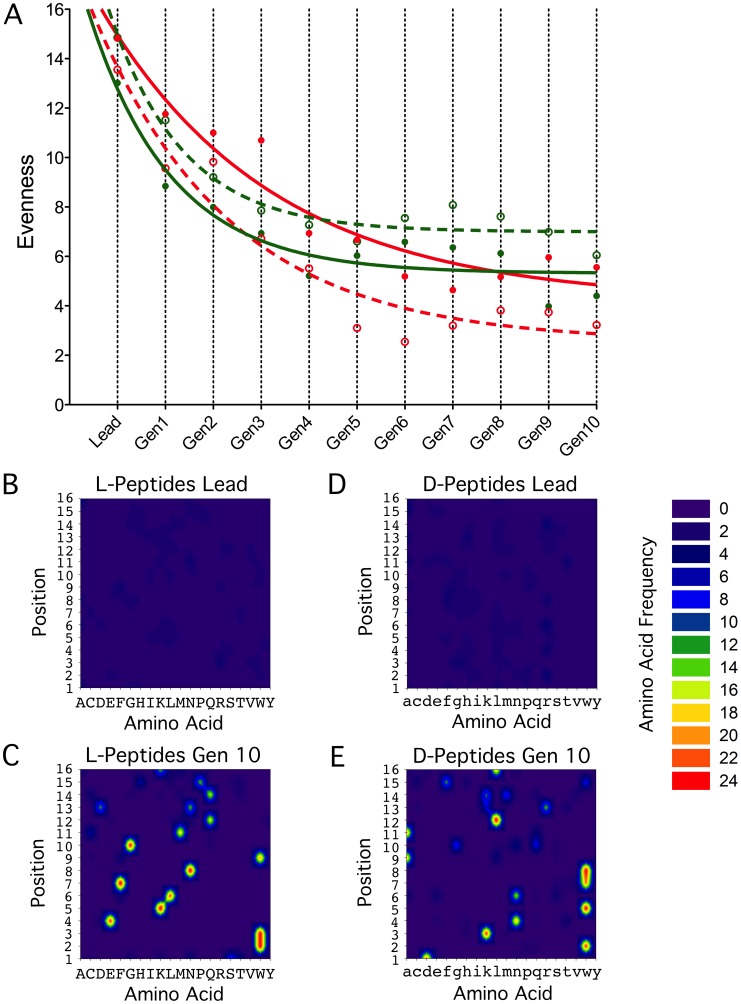
Emergence of consensus sequences. **A.** Change in evenness of the amino acid distribution in each generation. The diversity of the peptide sequences is expressed by Pielou's evenness index (full circles: complete populations of 200 peptides of each generation, open circles: subpopulations of 25 “fittest” peptides chosen as parents for the next generation; red: L-peptides, green: D-peptides). **B–E.** Formation of a consensus sequence for L- (**B**, **C**) and D-peptide populations (**D**, **E**). The frequencies of the individual amino acids (given in one letter code; capital letters for L amino acids, small letters for D amino acids) were determined in the lead peptides (**B**, **D**) and the 25 best G_M1_-binders of the 10^th^ generation (**C**, **E**). The sequence positions 1–16 are plotted versus the respective amino acid residues. Color codes show how often a certain amino acid is found at the respective position (“Amino Acid Frequency”): purple-blue staining indicates a uniform occurrence of all amino acids, yellow-red reveals frequent occurrence of a certain amino acid at the respective sequence position.

A careful balance between the decrease of diversity in the peptide population and the increase in fitness of the candidate peptides had to be preserved in order to prevent inbreeding. The evenness of amino acid distribution in the peptide populations was therefore continually monitored [25] (Figure 3 A) and the decrease in diversity in the populations was counteracted by an elevation of the mutation rate to 12% in order to prevent premature termination of the optimization process.

Even so, in 10 rounds of evolution consensus sequences had emerged (Figure 3 B–E) which could not be broken by a mutational rate of 12% and appeared to be a local fitness optimum reachable from the population sets of lead peptide sequences. Although the fraction of peptides which carried the consensus motif still increased at this point of the evolution - and hence the mean fitness of the population – no new sequence motifs were obtainable because of the lack of diversity in the peptide population and consequently no further optimization could be achieved. While we could observe basically no homology between the consensus motif and the respective sequence positions in the lead peptides, it is noteworthy that the amino acids identified by an alanine-scan in the lead peptides as “beneficial” for binding of the G_M1_-probe were - independent of their position - mostly aromatic (tryptophan, phenylalanine) and, to a lesser extent, apolar (leucine, isoleucine) and positively charged (arginine, lysine) amino acids. This tendency is reflected in the consensus motifs formed in generation 10 where the tryptophan content in the L-peptides rose to 18% and where the D-peptides even had a tryptophan at 50% of the N-terminal positions 1–8. Our data are consistent with other studies [26] where arginine, phenylalanine and tryptophan were found to be important for binding in phage-mutant experiments with non-labeled G_M1_.

**Figure 4:**
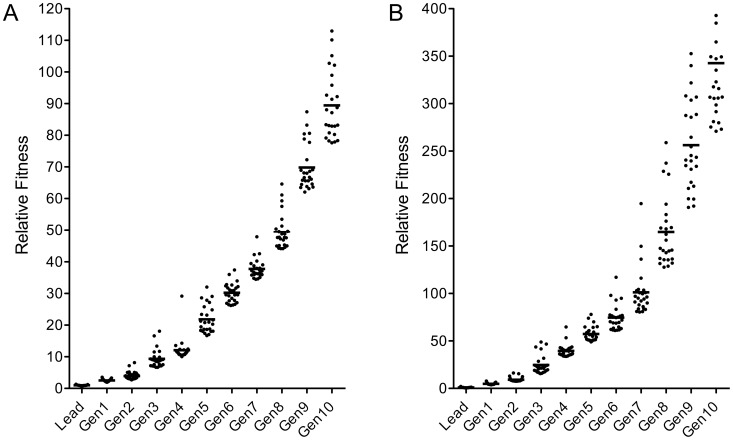
Increase in target-binding strength of peptide sequences in the course of the molecular evolution process. **A.** L-peptides **B.** D-peptides. The relative fitness (compared to the lead peptides) of the 25 best lysoG_M1_/Dy650 binding peptides from each generation is shown. Horizontal bars indicate the arithmetic mean of the 25 best fitness values of each generation.

In the course of the 10-round molecular optimization process performed the fitness of the candidate L- and D-peptide sequences progressed steadily as shown in Figure 4. The increase in fitness of the peptides could be determined by normalization of the fitness values of all peptides in one generation on the values of their respective parent peptides which were always synthesized and tested anew along with each filial generation (Figure 1). Since the parent peptides of a filial generation were identical to the fittest candidates of the previous generation a normalization chain over the entire evolutionary process was possible and eliminated potential synthesis-to-synthesis variations. This rendered data from different peptide array experiments comparable and also allowed readjustment of the laser intensity settings used for readout in the imager. The adaption of laser intensity used for the readout of the peptide arrays from different generations was necessary in order to stay in the maximal dynamic measuring range of the instrument. Over the total evolutionary process encompassing ten generations, the fitness of the 25 fittest candidates of each generation of L- and D-peptides increased steadily but the improvement did not follow a simple exponential growth curve. Logarithmic transformation revealed two exponential growth phases, a fast one over the first 5 generations and a second, slower one for the last five generations (Figure S2). Whether this deceleration is due to the increased mutation rate that was necessary to prevent inbreeding beyond generation 5 or whether it already represents the logistic growth typical for natural growth processes remains disputable. Over all ten generations an average fitness improvement factor per generation of 1.6 for the L- and 1.7 for the D-peptides was observed leading to a cumulative affinity improvement of about 100-fold for the L- and 400-fold for the D-peptide candidates (Figure 4). This gain in affinity was attained by synthesizing a total of just 2400 16mer L- and 2000 D-peptides out of a peptide space of 10^21^ permutations imaginable.

These results demonstrate that the evolutionary optimization of a peptidic 16mer G_M1_ ligand is possible, that the improvement progresses rapidly, that in just 10 generations of 200 peptides each a 400fold improvement in affinity is achievable and that both L- and D-peptides can be optimized this way. Such a dramatic and rapid improvement is not self-evident. Yokobayashi and colleagues [20] for instance achieved only a 3fold improvement over 6 generations when optimizing a 6mer peptidic trypsin inhibitor. The reasons for this discrepancy may reside in the length of the peptide since the peptide space of a 6mer peptide solely composed of proteinogenic amino acids is 10^14^-times smaller than that of a respective 16mer; the lead structure may already be close to perfection such that there is only little room for improvement; and there may be colliding features in the optimization parameters that per se preclude the existence of an optimal candidate. Such limitations must always be kept in mind when setting up an EA-based molecular evolution procedure. An EA optimizes a molecule within a preset selection of constraints, nothing less but also nothing more. Neither does it correct shortcomings in assay design, ill-chosen amino acid pools or peptide length nor does it introduce properties one has not asked for. For example, a G_M1_-ligand evolved in a solid phase assay where solubility was not an issue may not be optimally suited for soluble G_M1_-targeting systems but could perform well on a particulate vaccine delivery system or contrast agent. Likewise, an all-D peptidic G_M1_-ligand can be perfect for gastrointestinal applications where all-L-peptides are broken down quickly [27] but the same all-D-peptide may be toxic upon systemic application because of its high stability. Another difficulty in the application of the procedure presented here might arise when peptidic ligands for large protein targets are to be developed. Here, the pool of lead sequences must be chosen carefully in order to prevent crossing of leads directed against topologically different binding sites on the target surface. Yet, even though such limitations exist, the EA-based molecular optimization procedure presented in this work is superior to classical high-throughput screening approaches as the latter suffer from similar shortcomings without the time- and material-saving advantages of the evolution process.

In light of these considerations we are confident that the work presented in this study will be a valuable stimulus to fields where site specific delivery is crucial, such as drug targeting, molecular imaging and personalized medicine.

## Materials and Methods

Casein (Hammarsten grade) was obtained from BDH (via VWR International, Darmstadt, Germany). Cellulose membranes (Whatman 540) were from Whatman International (Dassel, Germany). Fmoc amino acid building blocks with side chain protecting groups if required (*t*Bu (serine, threonine and tyrosine), O*t*Bu (aspartic- and glutamic acid), Boc (lysine and tryptophan), Acm (cysteine), Trt (histidine, asparagine and glutamine), Pbf (arginine)) were obtained from Merck Biosciences (Bad Soden, Germany). Anhydrous hydroxybenzotriazole (HOBt) was from Dojindo Laboratories (Kumamoto, Japan) and dimethylformamide (DMF) from LGC Promochem (Wesel, Germany). N-methylpyrrolidone (NMP; Fluka, Taufkirchen, Germany) was deionised by treatment with dried ion exchange mixed bed resin AG501-X8 (Bio Rad Laboratories, München, Germany). Lysoganglioside G_M1_ was from Sigma-Aldrich (Taufkirchen, Germany) and DY650-NHS was from Dyomics (Jena, Germany). All other chemicals and solvents were of analytical grade and purchased from Sigma-Aldrich; they were used without further purification.

Fluorescence intensities were quantified with an Odyssey Infrared Imager running software version 2.1.12 (LI-COR Biosciences, Bad Homburg, Germany). Spatially addressable multiple peptide synthesis was carried out using a pipetting robot ASP 222 (Intavis Bioanalytical Instruments, Köln, Germany).

### Peptide libraries

Peptide arrays were synthesized onto cellulose membranes following a modified version of the procedure described by Frank [28]. The cellulose membranes were derivatized with epibromohydrine in dioxane (10% (v/v) containing 1% of 60% perchloric acid, 0.02 ml/cm^2^ membrane), for 3 h at room temperature (RT) according to Ast et al. [29]. The membranes were washed 3× with dioxane (0.13 ml/cm^2^ membrane, 5 min, RT) and subsequently exposed to a solution of 4,7,10-trioxa-undecane-1,13-diamine (20% (v/v) in DMF, 0.21 ml/cm^2^ membrane) for 3 h at RT. This solution was removed and the membranes were incubated in a solution of NaOMe in Methanol (5 M, 0.21 ml/cm^2^ membrane) for 30 min, washed 7× with aqueous methanol (0.21 ml/cm^2^ membrane, 5 min, RT) and air dried.

For definition of the synthesis areas, 0.1 µl of 0.2 M Fmoc-β-alanine-pentafluorophenyl (Pfp) ester (0.2 M Fmoc-β-alanine-Pfp-ester in NMP) were applied by the pipetting robot at defined positions on the membrane, then excess hydroxyl groups were blocked (“capping”) with a solution of acetic anhydride (Ac_2_O) and diisopropylethylamine in DMF (8% Ac_2_O, 15% diisopropylethylamine (v/v), 0.13 ml/cm^2^ membrane, 1 h, RT, rocking) and the membranes were washed 3× with DMF (0.13 ml/cm^2^ membrane, 3 min, RT). The Fmoc protecting groups were cleaved using piperidine (20% (v/v) in DMF, 1 ml/cm^2^ membrane, 20 min, RT). To confirm the presence of free amino functions in the spot areas, the membranes were washed 5× with DMF (0.13 ml/cm^2^ membrane, 3 min, RT), stained with bromophenol blue in DMF (0.01% (v/v), 0.13 ml/cm^2^ membrane, 10 min, RT, rocking), washed 3× with 100% ethanol (0.13 ml/cm^2^ membrane, 3 min, RT) and air dried.

The above capping, washing, Fmoc-cleavage and staining steps were repeated between all synthesis cycles from the definition of the synthesis areas (spots) onwards, but after the third synthesis cycle capping was performed for 20 min only using 2% Ac_2_O (v/v) in DMF.

For all peptide synthesis cycles, the amino acid building blocks were converted into their corresponding HOBt esters immediately before use by adding 1.25 moles diisopropylcarbodiimide per mole amino acid to a solution containing 0.4 M N-α-Fmoc-protected amino acid and 0.7 M HOBt in NMP (final concentration: 0.2 M amino acid, 0.35 M HOBt, 0.25 M diisopropylcarbodiimide) and allowing the mixture to react for 30 min at RT. Precipitates were removed by a short centrifugation step and 0.2 µl of these N-α-Fmoc-protected amino acid active esters were applied to the respective synthesis areas. Coupling of each amino acid was repeated 3 times and a minimum of 40 min reaction time was allowed in each synthesis cycle. After the last cycle the peptides were N-terminally acetylated with 2% (v/v) Ac_2_O in DMF (15 ml (0.13 ml/cm^2^ membrane), 20 min, RT, rocking).

Side chain protecting groups (except for Acm) were removed by immersing the membranes twice in a cleavage cocktail (50% (v/v) trifluoroacetic acid, 3% (v/v) triisobutylsilane, 2% (v/v) water in dichloromethane, 0.09 ml/cm^2^ membrane, 1 h each, RT, rocking). Subsequently, membranes were washed 4× with dichloromethane, 3× with DMF, 4× with 1 M acetic acid and finally 3× with 100% ethanol (each 0.13 ml/cm^2^ membrane, 3 min, RT). Membranes were air-dried, desiccated overnight *in vacuo* and stored in the presence of desiccant at −20 °C.

### G_M1_-binding assays

To obtain a fluorescent G_M1_ without modification of its carbohydrate part which is essential for ligand binding, we substituted the generic fatty acid of the ganglioside's ceramide moiety with a dark red fluorescent dye. Lysoganglioside G_M1_ (lysoG_M1_) was chosen as starting material as it already lacks the fatty acid residue and contains a free amino function instead. For preparation of the lysoG_M1_/DY650 conjugate, 200 µl of 2 mg/ml lysoG_M1_ in dry DMF were mixed with 225 µl of 2 mg/ml DY650-NHS in dry DMF, 7 µl of diisopropylethylamine were added, and the mixture was incubated for 36 h at 30°C under an argon atmosphere. The solvent was removed *in vacuo*, the residue was dissolved in 500 µl of acetonitrile/water (20% (v/v)) and purified by HPLC on RP-18 silica gel (A = water, B = acetonitrile, 20% B to 70% B, 1 ml/min, 60 ml, retention volume 40 ml).

For screening of the peptide libraries, the cellulose membranes carrying the peptide arrays were blocked with casein/PBS (1% casein in Dulbecco's PBS (D-PBS) (1.47 mM KH_2_PO_4_, 8.10 mM Na_2_HPO_4_, 137 mM NaCl, 2.68 mM KCl, pH 7.4) for 3 h at RT and incubated over night at RT with a solution of lysoG_M1_/Dy650 (approx. 10 ng/ml (approx. 5 nM)) in casein/PBS. Then they were washed at RT 6×10 min with D-PBS-Tween (D-PBS containing 0.5% (v/v) Tween20), 2×10 min with D-PBS and the bound lysoG_M1_/DY650 was quantitated on the wet membranes in an Odyssey Infrared Imager (excitation wavelength 680 nm, emission wavelength 700 nm). All experiments were performed in duplicate using 2 identical libraries that had been synthesized in parallel.

### Alanine scan experiments in the lead peptide sequences

Peptide sequences for alanine scan experiments were synthesized on cellulose membranes as described above. All sequence-positions in the L- and D-lead peptides were replaced with L-alanine for the L-peptides and D-alanine for the D-peptides, and the “point-mutated” sequences were probed for their lysoG_M1_/DY650 binding capacity as described above. An influence on the G_M1_ binding capacity due to an amino acid that had been replaced by alanine was assumed, if the fluorescence signal of the mutated peptide spot was increased or decreased by 50% in comparison to the original sequence. If the exchange of an amino acid residue lead to a decrease in signal of the mutated peptide by 50% the replaced residue was judged to be beneficial in G_M1_ binding and highlighted by boldface in table 1. If the exchange of a residue lead to an increase in signal of the mutated peptide by 50% the replaced residue was judged to be disadvantageous for binding in the original sequence and underlined in table 1.

### Generation of peptide sequences using the evolutionary algorithm

The work flow in the software for the evolutionary optimization of peptides is summarized in Figure 1 and depicted in detail in form of a 10-step-flow chart in Supplementary Figure S1. L-Peptide lead sequences for the evolution process were identified (step 1) by manual inspection from a library of 64 alleged G_M1_ binding peptides (Supplementary Table S1) which had been screened with fluorophore-labeled lysoG_M1_/Dy650 [17]. Eleven peptides (μ = 11) displaying a G_M1_-probe binding affinity above background, as determined by applying a statistically defined cut-off value [18], were character encoded using a 1-letter code for the respective amino acids (step 2), assigned fitness-values (step 3) according to their fluorescence signals in the assay and entered as lead peptides into the evolution process.

Different parameter settings in the EA - fitness scaling function, parent population size, crossover rate, number of fracture sites and mutation rate - were worked out in an empirical simulation study based on a “pseudo” fitness function and artificial peptide motifs and optimized experimentally in the first generations of the evolution process. The initial gaussian configuration interval of probabilities for recombination and mutation was equal for all positions in the peptide sequences [23]. During the evolutionary process, each setup of the recombination and mutation operators was generated within the pre-configured gaussian distribution interval [23]. As a starting point a crossover rate of 100% with one fracture site in each sequence and a mutation rate of 7% were chosen. In generation 1 two subpopulations of λ = 200 peptide sequences each were generated by applying different scaling functions to the fitness values (*x*) of the lead peptides (step 4); the first subpopulation (gen1sq) was created by using a square fitness scaling function:
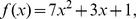
whereas the second subpopulation (gen1ex) was generated by using an exponential scaling function:
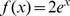
Both functions have been chosen to markedly scale the fitness values within the entire dataset range and therefore increase the selection pressure. Generation 2 (gen2, λ = 200 peptides) was created by using the μ* = 16 sequences with the highest fitness values from each, gen1sq and gen1ex, as parents. From this point onward, only the square fitness scaling function was used. In generation 3 the influence of the size μ* of the parent population resulting from manual inspection of the peptides (step 9) was investigated. For that purpose, a first subpopulation (gen3a, λ = 200 peptides) was created from μ* = 32 good parents out of gen2 while a second subpopulation (gen3b, λ = 200 peptides) was based on the μ* = 20 best sequences from gen2. The μ* = 25 sequences with the highest fitness values from gen3a and gen3b were taken as parent peptides for the creation of generation 4. For all the following generations (gen5–gen10, λ = 200 peptides each), always the μ* = 25 best motifs of the entire population were used as parents. From generation 6 onwards (gen6–gen10, λ = 200 peptides each) the mutational rate was elevated to 12% but the crossover rate of 100% and the single fracture site were kept.

For identification of D-peptide lead sequences, the 64 alleged G_M1_ binding peptides were synthesized in form of their retro-inverso peptide isomers and the resulting library of D-peptides was analyzed as described for the L-peptides. The D-peptide lead sequences were selected accordingly (Supplementary Table S1). For the optimization process of the D-peptides, the parameter settings were adopted from the procedure conducted with the L-peptides (crossover rate 100%; mutation rate 7% in gen 1–5, 12% in gen 6–10; square scaling function). From the μ = 13 lead peptides selected as a starting population, λ = 200 filial peptides were generated in gen1. The μ* = 17 fittest peptides from gen1 were chosen as parent peptides for gen2, for all the following generations (gen3–gen10, λ = 200 peptides each) the best μ* = 25 peptides were selected as parent sequences.

For the L- and the D-peptides the evolutional process was continued until a consensus motif was reached (step 10) and the evenness in the populations had reached a low plateau (Figure 3). All peptides synthesized and analyzed in this evolutionary process are listed in Supplementary Table S2.

### Determination of evenness

In all populations of D- and L-peptides, the evenness was calculated which is a measure of the population diversity. To do so, the frequency f of any individual amino acid (i) at each sequence position in a population of peptides was counted, and the species richness S (number of different amino acids which occur in total at this position) was determined. The individual amino acid frequency f_i_ was divided by the population size (N) (i.e. number of peptides in the respective population) to compute P_i_ (P_i_ = f_i_/N) which is the relative frequency of an amino acid in a given sequence position. With the relative frequency P for each amino acid (i) which is present at this sequence position, the Shannon Index (H) can be determined:
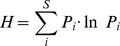
On the basis of the Shannon index H the Pielou evenness index (E) (E = H/H_max_) [25] can be calculated with H_max_ being the value for H in case that all amino acids available are present and equally distributed at this sequence position (H_max_ = lnS). The evenness index E can assume values between 1 (all amino acids present in equal numbers at the respective sequence position, i.e. “even distribution” of all amino acid species) and 0 (only one amino acid present at the respective sequence position, i.e. “maximally uneven distribution” of the amino acid species). For the 16 mer peptides the evennesses per position were summed up to calculate the population evenness.

## Supporting Information

Figure S1 **Flow chart of the evolutionary algorithm for function-driven peptide optimization.** A population of μ lead peptides is chosen (step 1) and character encoded (step 2). To each peptide, a fitness value is assigned according to the results of the biochemical assay (step 3). The fitness values assigned are scaled by a fitness scaling function (step 4) and fitness proportional selection by stochastic universal sampling (SUS) of the peptides is performed to create a mating pool of peptide sequences (step 5). Sequences from that pool are λ-times recombined with gaussian variation of the recombination points (step 6), and the resulting sequence motifs are then mutated with specific gaussian variation (step 7) to establish a filial generation of λ peptides. The peptide sequences created that way are synthesized in parallel and their fitness is determined in the biochemical assay (step 8). The results are manually inspected to select μ* candidates (step 9) to act as parent peptides for the next generation. This process is repeated until optimized peptides are obtained (step 10). The character encoding (step 2) of the peptide sequence data is a more intuitive one in comparison to binary coding which has often been used for genetic algorithms. Character encoding ensures a higher efficiency in the coding space and an easier manual inspection of the results. The stochastic universal sampling (SUS, step 5) as performed here is a state of the art selection method used for genetic algorithms (GAs) and is used as a GA-like internal cycle 1 to select the parents for each recombination repeatedly - depending on their fitness status - from the mating pool. The evolutionary strategy (ES)-like general cycle 2 starts with a number of μ lead peptides or with μ* manually selected ones from the overall population in each following generation. The default gaussian probability onset of the recombination (step 6) and mutation (step 7) operator configuration was determined by a simulation study based on a “pseudo” fitness function and an artificial peptide-motif. The values of μ and λ reflect the generation changes and selection pressure. In that regard the manual selection of e. g. μ* = 25 from an intermediate population size λ = 200 stands for a selection pressure of 8. In contrast to standard ES the population size of λ peptides is here generated by breeding out of a limited mating pool, i.e. λ-times recombination (step 5+6) of the manually selected μ or μ*-peptides.
(PDF)

Figure S2 **Fitness ”growth„ over 10 generations of evolution.** Mean fitness of the 25 best candidates of each generation (normalized to fitness of lead peptides = 1) is shown after logarithmic transformation. Ideal exponential growth is reflected in a linear relationship between ”log Fitness„ and “Generation„. Here, improvement of fitness can be divided into two “exponential” phases: “fast” growth in generation 0–5, “slower” growth in generation 5–10.
(PDF)

Table S1 **Identification of lead sequences for the molecular evolution of G_M1_-binding peptides.**
(PDF)

Table S2 **List of all peptides generated in the course of the evolutionary process of optimization for G_M1_-binding.**
(PDF)
