# Supplementary figures and images for: Molecular Evolution of Peptide Ligands with Custom-Tailored Characteristics for Targeting of Glycostructures
Source: PLoS Comput Biol. 2012 Dec 13;8(12):e1002800. doi: 10.1371/journal.pcbi.1002800 (PMC3521706; doi:10.1371/journal.pcbi.1002800)

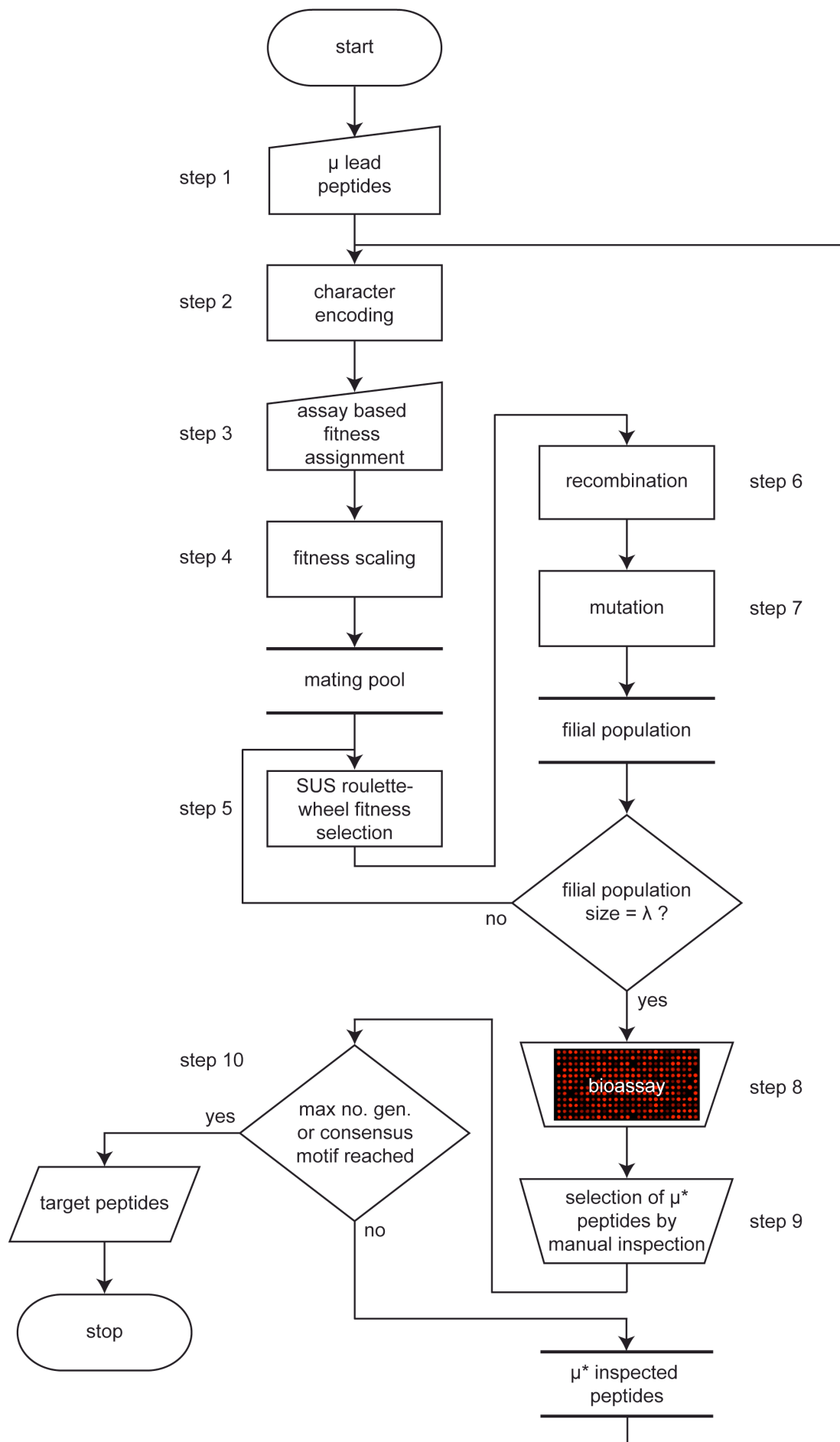

Supplement: Figure S1 — Flow chart of the evolutionary algorithm for function-driven peptide optimization. A population of μ lead peptides is chosen (step 1) and character encoded (step 2). To each peptide, a fitness value is assigned according to the results of the biochemical assay (step 3). The fitness values assigned are scaled by a fitness scaling function (step 4) and fitness proportional selection by stochastic universal sampling (SUS) of the peptides is performed to create a mating pool of peptide sequences (step 5). Sequences from that pool are λ-times recombined with gaussian variation of the recombination points (step 6), and the resulting sequence motifs are then mutated with specific gaussian variation (step 7) to establish a filial generation of λ peptides. The peptide sequences created that way are synthesized in parallel and their fitness is determined in the biochemical assay (step 8). The results are manually inspected to select μ* candidates (step 9) to act as parent peptides for the next generation. This process is repeated until optimized peptides are obtained (step 10). The character encoding (step 2) of the peptide sequence data is a more intuitive one in comparison to binary coding which has often been used for genetic algorithms. Character encoding ensures a higher efficiency in the coding space and an easier manual inspection of the results. The stochastic universal sampling (SUS, step 5) as performed here is a state of the art selection method used for genetic algorithms (GAs) and is used as a GA-like internal cycle 1 to select the parents for each recombination repeatedly - depending on their fitness status - from the mating pool. The evolutionary strategy (ES)-like general cycle 2 starts with a number of μ lead peptides or with μ* manually selected ones from the overall population in each following generation. The default gaussian probability onset of the recombination (step 6) and mutation (step 7) operator configuration was determined by a simulation study ba [file pcbi.1002800.s001.pdf]

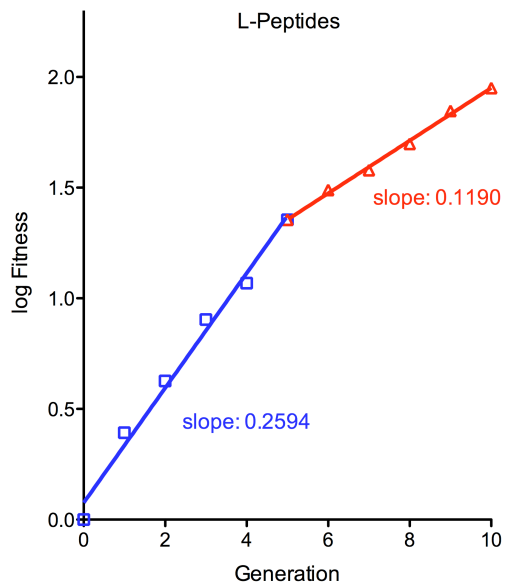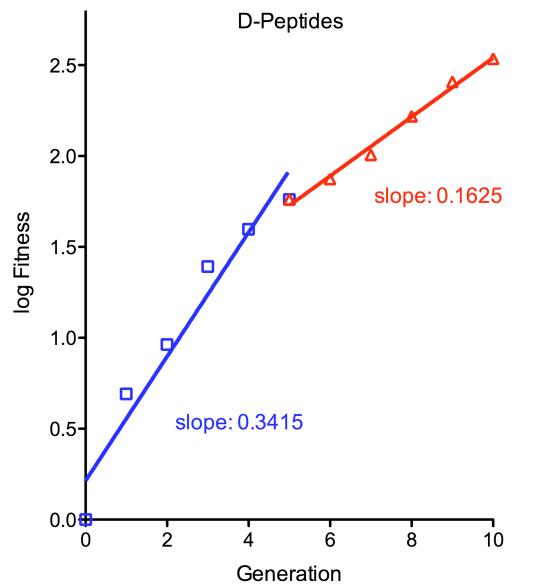

Supplement: Figure S2 — Fitness ”growth„ over 10 generations of evolution. Mean fitness of the 25 best candidates of each generation (normalized to fitness of lead peptides = 1) is shown after logarithmic transformation. Ideal exponential growth is reflected in a linear relationship between ”log Fitness„ and “Generation„. Here, improvement of fitness can be divided into two “exponential” phases: “fast” growth in generation 0–5, “slower” growth in generation 5–10. (PDF) [file pcbi.1002800.s002.pdf]
