# Supplementary material for: Molecular Evolution of Peptide Ligands with Custom-Tailored Characteristics for Targeting of Glycostructures
Source: PLoS Comput Biol. 2012 Dec 13;8(12):e1002800. doi: 10.1371/journal.pcbi.1002800 (PMC3521706; doi:10.1371/journal.pcbi.1002800)
Supplement: Table S1 — Identification of lead sequences for the molecular evolution of GM1-binding peptides. (PDF) [file pcbi.1002800.s003.pdf]

Table S1:

Identification of lead sequences for the molecular evolution of G_{M1}-binding L-peptides. 64 Known or alleged G_{M1} binding motifs obtained from the literature (compilated in [1]) were synthesized as L-peptides on filter membranes and screened for G_{M1} binding with lysoG_{M1}-DY650-conjugate. Fitness values reflect the fluorescence intensities of individual peptide areas as determined in a Licor Odyssey Infrared Imager (arbitrary units). Peptides which showed fluorescence signals significantly above background [2] were selected as lead peptides (written in boldface).

No.	Sequence	Fitness	No.	Sequence	Fitness
1	VEVPGQHIDSQKKAIE	5,14	33	YSEIAIQMMMHMQPKE	7,62
2	VPGQHIDSQKKAIERM	22,09	34	CEFLVKEVTKLIDNNK	50,57
3	GQHIDSQKKAIERMKN	38,86	35	FLVKEVTKLIDNNKTE	57,67
4	HIDSQKKAIERMKNTL	40,54	36	VKEVTKLIDNNKTEKE	17,05
5	DSQKKAIERMKNTLRI	57,76	37	EVTKLIDNNKTEKEIL	5,61
6	SQKKAIERMKNTLRIA	55,99	38	ASQKRPSQRHGSKYLA	56,15
7	SQKKAIERMKNTLRIA	13,64	39	QKRPSQRHGSKYLATA	45,20
8	VPGSQHIDSQKKAIER	24,05	40	RPSQRHGSKYLATAST	36,13
9	GSQHIDSQKKAIERMK	41,53	41	SQRHGSKYLATASTMD	17,07
10	QHIDSQKKAIERMKDT	16,75	42	RHGSKYLATASTMDHA	31,38
11	IDSQKKAIERMKDTLR	33,05	43	GSKYLATASTMDHARH	55,51
12	SQKKAIERMKDTLRIA	37,23	44	KYLATASTMDHARHGF	59,64
13	SYTESMAGKREMVIIT	9,60	45	LATASTMDHARHGFLP	50,61
14	TESMAGKREMVIITFK	49,04	46	TASTMDHARHGFLPRH	58,55
15	SMAGKREMVIITFKSG	48,01	47	STMDHARHGFLPRHRD	49,57
16	LEVPTDGNAGLLAEPQ	5,36	48	MDHARHGFLPRHRDTG	61,29
17	VPTDGNAGLLAEPQIA	6,87	49	HARHGFLPRHRDTGIL	52,37
18	TDGNAGLLAEPQIAMF	8,39	50	RHGFLPRHRDTGILDS	29,82
19	GNAGLLAEPQIAMFCG	17,33	51	GFLPRHRDTGILDSIG	23,39
20	AGLLAEPQIAMFCGRL	30,50	**52**	**LPRHRDTGILDSIGRF**	**104,05**
21	LLAEPQIAMFCGRLNM	27,92	53	KYEVNWKTHEIKVKGQ	63,41
**22**	**AEPQIAMFCGRLNMHM**	**75,37**	**54**	**YEVNWKTHEIKVKGQN**	**77,44**
**23**	**PQIAMFCGRLNMHMNV**	**83,79**	55	AEYHNTQIHTLNDKIF	18,80
24	IAMFCGRLNMHMNVQN	60,97	56	ATFQVEVPGSQHIDSQ	11,26
25	MFCGRLNMHMNVQNGK	56,71	**57**	**AKVEKLCVWNNKTPHA**	**67,96**
26	CGRLNMHMNVQNGKWD	22,38	**58**	**GALSETYSFIKEYHNT**	**78,21**
27	RLNMHMNVQNGKWDSD	9,66	59	ESLAGKREQSDIHQSG	12,39
28	NMHMNVQNGKWDSDPS	4,79	**60**	**ASDWYDEMLTWNIHGA**	**66,96**
**29**	**DFRRLPGAFWQLRQPA**	**94,08**	61	DGTVSTHECRLEYKYP	43,00
**30**	**GWWYKGRARPVSAVAA**	**72,12**	62	FSHSGEGDMIWERMID	12,94
**31**	**VWRLLAPPFSNRLLPA**	**64,39**	**63**	**HHCSILKEVWHVKKLG**	**74,12**
32	SQYSEIAIQMMMHMQP	9,37	64	PDQDGVVTAMHGFTHN	14,38

Identification of lead sequences for the molecular evolution of G_{M1}-binding D-peptides. For the evolution of D-peptides the 64 alleged G_{M1} binding motifs of above were transformed from L-peptides into their retro-inverso form (reversal of sequence and use of D-amino acids). Screening of the D-peptides and assignation of fitness values was performed as for the L-peptides.

No.	Sequence	Fitness	No.	Sequence	Fitness
1	eiakkqsdihqgpvev	1,53	33	ekpqmhmmmqiaiesy	1,84
2	mreiakkqsdihqgpv	4,35	34	knndilktvekvlfec	5,12
3	nkmreiakkqsdihqg	10,05	35	etknndilktvekvlf	7,20
4	ltnkmreiakkqsdih	11,28	36	eketknndilktvekv	0,65
5	irltnkmreiakkqsd	10,09	37	lieketknndilktve	0,20
6	airltnkmreiakkqs	12,97	38	alyksghrqsprkqsa	17,01
7	iakkqsdihqsgpvev	2,59	39	atalyksghrqsprkq	18,53
8	reiakkqsdihqsgpv	4,91	40	tsatalyksghrqspr	16,25
9	kmreiakkqsdihqsg	11,98	41	dmtsatalyksghrqs	11,63
10	tdkmreiakkqsdihq	6,83	42	ahdmtsatalyksghr	10,07
11	rltdkmreiakkqsdi	5,56	43	hrahdmtsatalyksg	3,90
12	airltdkmreiakkqs	11,73	44	fghrahdmtsatalyk	35,71
13	tiivmerkgamsetys	1,63	**45**	**plfghrahdmtsatal**	**87,02**
14	kftiivmerkgamset	26,79	**46**	**hrplfghrahdmtsat**	**64,35**
15	gskftiivmerkgams	21,83	**47**	**drhrplfghrahdmts**	**90,77**
16	qpeallgangdtpvel	1,45	**48**	**gtdrhrplfghrahdm**	**82,55**
17	aiqpeallgangdtpv	1,50	**49**	**ligtdrhrplfghrah**	**77,87**
18	fmaiqpeallgangdt	17,73	50	sdligtdrhrplfghr	15,55
19	gcfmaiqpeallgang	19,47	51	gisdligtdrhrplfg	15,69
**20**	**lrgcfmaiqpeallga**	**50,05**	**52**	**frgisdligtdrhrpl**	**59,27**
21	mnlrgcfmaiqpeall	44,35	53	qgkvkiehtkwnveyk	22,36
**22**	**mhmnlrgcfmaiqpea**	**53,31**	54	nqgkvkiehtkwnvey	24,24
**23**	**vnmhmnlrgcfmaiqp**	**51,71**	**55**	**fikdnlthiqtnhyea**	**75,51**
24	nqvnmhmnlrgcfmai	7,94	56	qsdihqsgpvevqfta	0,92
25	kgnqvnmhmnlrgcfm	8,74	57	ahptknnwvclkevka	8,22
26	dwkgnqvnmhmnlrgc	4,67	**58**	**tnhyekifsyteslag**	**102,36**
27	dsdwkgnqvnmhmnlr	4,46	59	gsqhidsqerkgalse	-1,23
28	spdsdwkgnqvnmhmn	-1,82	60	aghinwtlmedywdsa	9,73
**29**	**apqrlqwfagplrrfd**	**157,50**	61	pykyelrcehtsvtgd	6,17
30	aavasvprargkywwg	28,12	62	dimrewimdgegshsf	2,29
**31**	**apllrnsfppallrwv**	**52,06**	63	glkkvhwveklischh	12,78
32	pqmhmmmqiaiesyqs	1,57	64	nhtfghmatvvgdqdp	9,23

References:

[1] Röckendorf N, Bade S, Hirst T R, Gorris H-H, Frey A (2007) Synthesis of a fluorescent ganglioside G_{M1} derivative and screening of a synthetic peptide library for G_{M1} binding sequence motifs. Bioconjugate Chem 18: 573-578.

[2] Frey A, Di Canzio J, Zurakowski D (1998) A statistically defined endpoint titer determination method for immunoassays. J Immunol Methods 221: 35-41.
