# Supplementary material for: Molecular Evolution of Peptide Ligands with Custom-Tailored Characteristics for Targeting of Glycostructures
Source: PLoS Comput Biol. 2012 Dec 13;8(12):e1002800. doi: 10.1371/journal.pcbi.1002800 (PMC3521706; doi:10.1371/journal.pcbi.1002800)
Supplement: Table S2 — List of all peptides generated in the course of the evolutionary process of optimization for GM1-binding. (PDF) [file pcbi.1002800.s004.pdf]

Table S2: List of all peptides generated in the course of the evolutionary process of optimization for G_{M1}-binding. All peptides were synthesized on cellulose membranes (200 peptides each generation) and analyzed for target binding with a fluorophore-labeled G_{M1}-derivative. Fitness values were assigned to each peptide according to fluorescence signals obtained in this binding assay. Peptides are listed from the top-ranked peptide (1) to the peptide sequence displaying the lowest binding affinity for the G_{M1} probe (200) for each optimization cycle ("generation"). The top candidates of each generation which were selected as parent peptides for the next generation are written in boldface, these peptides (designated "lead peptides") were synthesized again onto the cellulose membrane carrying the filial peptides to enable standardization. Fitness values for all peptides are given, both measured fluorescence intensities (x; arbitrary units) and values standardized across all generations (c) for direct comparison of peptides from different generations.

L-peptide sequences

	Generation 1a	x	c	Generation 1b	x	c	Generation 2	x	c	Generation3a	x	c
1	**GWWYKGTAFWQLRQPG**	61,99	2,18	**YPRHRTYGIYYCNVRF**	71,89	2,52	**GWWEKLCVWGRLNMHM**	181,90	5,85	**GWWEKLCVWGRQNQPA**	252,14	9,61
2	**DFRRLKGAFWQSIGRF**	59,08	2,07	**DPMQRGTGILFSIYRF**	67,81	2,38	**MPRHRDTEVWAVSKLG**	160,27	5,15	**GWWEKLCAFWDFCERK**	217,07	8,27
3	**AEVNWKGAFWQLRQPA**	55,82	1,96	**LVRHRLRGILKSIFRF**	64,09	2,25	**AEVNWKGAFWDFIERK**	116,19	3,74	**GWWEKLCVWGRLNMHM**	188,10	7,17
4	**VWRLLLKELWHVKKLG**	54,15	1,90	**MPRHRDTGYLVSIGRF**	63,98	2,25	**AKVEKLCVWWWNIHGA**	113,47	3,65	**VWRELLKAWGRLNMHM**	186,03	7,09
5	**AKVEKLCVWWSVKKLG**	52,95	1,86	**LGYHRATLNFDFIGRK**	63,02	2,21	**LDRAYAKWCLWMFTDF**	112,99	3,63	**GWWEKLCVWGRLNMHG**	175,18	6,67
6	**PQIAMFCAFWQLRQRA**	51,37	1,80	**GKTHRATRLLDYIGRY**	54,91	1,93	**DFQRWKGAFWQLRQPA**	108,95	3,50	**GWWEKLCVWGRLNMHM**	170,99	6,51
7	**AKVEKLCAFWQLRQPA**	50,26	1,76	**LPTFYYTGFLCSIGDC**	54,70	1,92	**VWRLLLKAFWQLRQPG**	100,93	3,24	**VWWEKLCVWGRQNMHM**	161,66	6,16
8	**DFRRLLKEVWHVNKLG**	48,30	1,70	**LPRLRDFVILFSIERY**	53,94	1,89	**VWRLLLKELWNVKKLG**	98,12	3,15	**GWWEKLCVWGQLRQSN**	158,72	6,05
9	**GWWYKGTGILVLIGMF**	47,66	1,67	**LRFACRTYILDVIGRC**	53,68	1,88	**KKFFYYTGFLGSIGDC**	97,21	3,13	**GWWEKLCVWGRLNQPA**	142,42	5,43
10	**LPRVKNTGILAFIGRF**	46,91	1,65	**LPRQRDTGILVVIYRQ**	51,40	1,80	**EKVEKLGAFWGSIGRF**	96,06	3,09	**AEVNWRGAFWCSCKLG**	142,02	5,41
11	**GWWYKGTARPWNIHGA**	45,44	1,59	**LPRHDYTMILLTYGRF**	50,36	1,77	**DWFLKLCVWWSVKKLG**	93,96	3,02	**GWWEKLCVWGRLIGDC**	134,85	5,14
12	**DFRRLPKAFWQLYHNT**	43,47	1,53	**LTRRNLTGILSSTYRF**	46,19	1,62	**TINKAWTLNFDFIGRK**	89,12	2,87	**AEVHRDSEVWAVSKLG**	133,99	5,11
13	**LWWYQGRARPVSAPHA**	42,21	1,48	**DPRHRNFGVQGLIFRF**	45,26	1,59	**SEVRWKGAFWQLRWDN**	87,58	2,82	**GWWEYLCSWGRLNMRK**	131,44	5,01
14	**GALSETYSLTWEYGNA**	41,54	1,46	**KLRVRDFFILDGYGRF**	43,72	1,53	**GWWHRTYGIYYVNVRF**	79,03	2,54	**AEVNWKGAWWWNIHGA**	125,98	4,80
15	**EKVEKLCVWGRLNMHM**	40,29	1,41	**RVRHAKTLILYSIGRF**	43,67	1,53	**LPKFYYTGFLCSIKLG**	78,33	2,52	RKFFYYTGFLGVKKLG	125,50	4,78
16	**GALSETYSFINRLLPA**	40,22	1,41	**KKFTRDTGILKSIGFF**	42,59	1,49	**GWWYKGSAFWQVIGRC**	76,42	2,46	DFQRWLCVWWWNIHGA	124,94	4,76
17	GALCVTYSFSMETHNT	39,37	1,38	**LPRHRSTLIQYGIRFF**	41,71	1,46	**DFERLKGAFWWMFWDN**	74,18	2,38	DFQRWKGAFWWMFWDN	122,98	4,69
18	VWRLLFPPFSNRLLSA	39,32	1,38	LPYHRDTKIAFFGGKF	39,23	1,38	**LPVNWKGAFWQLRQEA**	73,95	2,38	MPRHRDTEVWAVSKLG	115,57	4,40
19	YEVRWKTHEIKVKGRF	38,68	1,36	YEVNWKTHEIKVKGQN	37,41	1,31	**AFVMKLCVWTWEYGNA**	71,48	2,30	LDRHRDTEVWAVSKLG	114,21	4,35
20	GALSETYSFIKGYHNT	38,56	1,35	GALSETYSFIKEYHNT	36,93	1,30	**AKVEKACVWWSVKKLG**	71,16	2,29	GWKFYLCVWGRLNMLG	114,00	4,34
21	GWWNWMTHEIKVKGQN	38,54	1,35	LERHRMTGKLTFIVRF	36,92	1,30	***DFRRLKGAILFSIYRF***	69,56	2,24	GWWEKLCVWGRLNKLG	111,04	4,23
22	GALSETSAFWQLRQPA	38,30	1,34	LPRHRETGFYVSIGAF	36,41	1,28	***GDRAYGKWCLWMFWDN***	69,34	2,23	AKVEKLCVWWWNIHDC	110,05	4,19
23	LWRRLPDAFWQLRKPA	37,84	1,33	LAFFRDTGIRDSIGRR	36,08	1,27	***GWWYKATAFAYCNVAF***	69,18	2,22	GWWCKYCVWGWNIHGA	109,99	4,19
24	AEPQIAGAFWQLRQPA	37,66	1,32	LYRHVFTVALDAIGLF	35,91	1,26	***LWWYEGCAFWQLRQRA***	68,53	2,20	SEVRWKGAFWAVSKLG	108,73	4,14
25	FWRLLAPPFIKEYHNV	37,15	1,30	MARHRYAGLFDSIGMR	33,87	1,19	***LPTFRLEGILKSIFRF***	67,70	2,18	SERAKAKWCLWMFTDC	107,37	4,09
26	GWWSETCSFIKEYHNT	37,15	1,30	LPRHSDTGILSGILVF	30,41	1,07	***ERFARLCVWWSVKKLG***	67,64	2,17	DFQRWAKWCLWMFTDR	107,27	4,09
27	GDWMKGREVWHVKKLG	37,13	1,30	LPGMRLTKILYSAGRF	29,98	1,05	***GWQYKGTALLDYIGRY***	67,25	2,16	GWWEKLCVWGRMVGDC	107,24	4,09
28	GACSINKEVWHVKKLG	37,13	1,30	LTRHRDTGIYQTILSF	29,96	1,05	***LVFHRLRGILFFIYRF***	67,25	2,16	GWWEKLCVFWGSIGRF	104,88	4,00
29	AKVEKGCGILDSIGRF	36,33	1,28	CPRHEDGYILRSYGRF	29,23	1,03	***GKTHRACVWWSVKKLG***	66,35	2,13	GWQRWKGAFWQLRQPA	104,73	3,99
30	GALSETYSFIKSIGRF	36,04	1,27	FGSRRTTGILKSIGRM	29,01	1,02	***AYVNWNTGILAFIGRF***	64,57	2,08	AWDLLLKAFWQLRQPG	104,64	3,99
31	YELNWTYSFIKEYHNT	35,29	1,24	LRRKRSTGKTDSILRF	28,45	1,00	***GWWYKGTAFWQLTYYF***	62,00	1,99	REVHRDTEVWAVSKLG	103,73	3,95
32	YEVQWKTHEIKVKGQF	33,34	1,17	LVVSDCTAIRRSIGRF	28,42	1,00	***VWRLLGTAFWQDRQTG***	60,19	1,93	GWWEKLSAFWQVIGRC	102,63	3,91
33	GWWYKGRARMVSIGRK	33,00	1,16	LGRRFDTLLLESILRM	27,98	0,98	GWWYKGDGILVLIYRF	59,96	1,93	MPRHNWTLNFDFIGRK	99,66	3,80
34	GALSETYSFIRLNYGM	31,33	1,10	DFRRLPGAFWQLRQPA	27,94	0,98	YPRRNLTGILYATYRF	59,07	1,90	AEVNWKGAFWDFIERK	99,51	3,79
35	LALSETYSFIKEYHNT	31,15	1,09	FPRHRDYGILDSIGFR	27,49	0,96	LDRAYAKWCLWMEWRF	59,03	1,90	AKVEKLCVWWWNIHGA	98,99	3,77
36	CHCSILKEVSNRLKPA	30,87	1,08	LLRHRDTVILLKIDRF	26,64	0,93	MPRLRDFVIRFSIERY	57,94	1,86	GWWEKLCVWGATSKLG	98,67	3,76
37	DFRRLPGAFWYEYHNT	30,18	1,06	LFRRRDTKKLDSIGRK	25,82	0,91	RVRHATYGIYYCNVRF	57,93	1,86	LPKFYAKWCLWCFTCF	98,42	3,75
38	YEVNWNTHEIKVIGRF	30,09	1,06	LPRHTKTTILDSIGLF	25,53	0,90	GWWHRNFGYQGLIFRF	56,34	1,81	DFGRLKGAFWWMIERK	97,15	3,70
39	VWRLLFLPGARRGLPA	29,95	1,05	LLRHRRTGILKSIYMM	25,16	0,88	VWRLLLDGFLCSIGDC	55,75	1,79	DFWEKLCVWGFLNMHF	96,06	3,66
40	PQIAMFCGRLNMHNNG	29,49	1,04	LMAHRGQGIYDSIGRF	24,59	0,86	AKVEKLCVWLARIGRF	55,33	1,78	SERHRDTEVWAVSKLG	95,05	3,62
41	VWWYKGRARMVSAVAA	29,24	1,03	LVRDKKTGILDQIRRK	24,59	0,86	YDRAYAKWCFWMFWDN	55,21	1,77	VWWEKLCVAGRLFSHM	92,78	3,53
42	LPRHRDTGIVWNIHGA	29,08	1,02	SERHRDTGILFTILRQ	24,29	0,85	LVKHRLRGILFSIYRF	55,18	1,77	MPRHRTYGIYYVNVRF	92,76	3,53
43	AEPQIAMFILDSIGRF	28,94	1,02	LPRFTSTGFCKSNGKL	23,84	0,84	DFRRLLKEVWHLRQDA	54,97	1,77	MVVNWKGAFWDFIERK	91,49	3,49
44	GWWYKPGAFNQLRQPA	28,54	1,00	LPEHRVTLILDKVFRL	23,55	0,83	LGVEKLCAFWQLRQPA	54,93	1,77	DPRHRDTEVWAVSKLS	90,75	3,46
45	AALSETYSFIKEYHNT	28,14	0,99	KYSTRDTKILDRSGFF	23,00	0,81	LVRHRLFGFLCSIGDC	53,70	1,73	AEVNWKGWCNWMFTDF	89,56	3,41
46	GACSILKEVWHVKKLG	27,85	0,98	LPYHSQTGVLFKICQK	22,77	0,80	AKVFKLCAFWQLRQFF	53,52	1,72	DWFLRLCVWGRLNSHM	88,49	3,37
47	ASDWLPGAFWQLRQPA	27,64	0,97	LQRHRSCGILYSIGQM	22,71	0,80	GWWYMGTGILFSIMRF	53,29	1,71	GWWEKLCVWWQLRWVN	87,41	3,33
48	YFRRLPCAFWQLRQPA	27,50	0,97	LPRHRESGIQLTITRF	22,19	0,78	YPRHYAKWCLWMFWDE	52,49	1,69	LDRAGAKWCLWMFTDF	86,89	3,31
49	PQIAMFCGRFNMHMNA	27,30	0,96	NPCHRDLGILDTFGRR	20,93	0,73	YPRHRTYGIYYCNVRF	52,45	1,69	GGWLLLKAFWQLRQPG	86,53	3,30
50	ASCSILKEVWHVKKRG	27,23	0,96	DPVRRDYGILVQIGRF	20,42	0,72	ARFACRFYILDVINRC	51,21	1,65	GEVRWKGAFWQLRWDN	86,20	3,28
51	PQIAMFCGRWQLRQPD	27,19	0,95	LERQRDQGILDSIRRS	20,25	0,71	AKRRLTGAFWQSIGRF	50,23	1,61	SEVRWKGAFWQLKKLG	86,16	3,28
52	GALKILKEVWHVKKLG	26,94	0,95	LPRHSEMGILGSIGRY	20,24	0,71	SALSETYSFINRLMHM	49,84	1,60	DFQRWKGAFWQLQQPA	85,88	3,27
53	AEPQIAMFILDSIGRF	26,76	0,94	LFRHGDTGISDSKGRF	20,21	0,71	DPRHRQFGVQGYIFRF	49,06	1,58	VWRLLLKELWNYKKLG	85,77	3,27
54	GALSETYSFIKEIGRF	26,53	0,93	MPRHRDTGNLNRILRS	20,06	0,70	GWWYNGTAFWQLRQGG	47,54	1,53	MPRHRDTEVWAVSKLG	84,49	3,22
55	YSVNWKTHEIKVKGPA	25,96	0,91	LPRHRDTGILDSIGRF	19,82	0,70	MPRHRDTGYLVSIGRF	47,29	1,52	GWWEKAVVWWSVKKLG	84,28	3,21

56	DFRRLPGAFWVSAVAA	25,53	0,90	LPRQARTTIEDSIRYF	19,69	0,69	DWWYKGTARAWNIHGA	47,26	1,52	SWEEKLCVWGRLNMHM	83,93	3,20
57	AKVEKLCVWNNKTVHF	25,27	0,89	LLRRRDFAILQSIQCF	19,56	0,69	YPRHRTYGIYYCNVRF	47,07	1,51	DWFLKLCVWWSRKYLA	82,94	3,16
58	YEKNWKTHEIKVKLYK	24,95	0,88	LPEHTDTYIFNSIGRN	19,48	0,68	AKVEKLCAFWQFRVRF	46,77	1,50	LPVEKLTAFWGSIGRF	82,74	3,15
59	AKVECLCVWNDSIGRF	24,79	0,87	LPLVRDFGLVDSIQRF	19,39	0,68	GWRLLLKELCHVKKLG	46,46	1,49	AKWFKECVWGKLNMHM	82,09	3,13
60	PQIFMLCVWNNKTPHA	24,78	0,87	LKRHRDTAILMSKCRF	19,03	0,67	PQIAMFCAFWQQLLPA	45,94	1,48	LDREKLCVWWWNQHGA	81,87	3,12
61	HHCSIRKTVWHVKKLA	24,75	0,87	LPRHRDCKKVDRIGRL	19,01	0,67	GRTHRATRILFSIERY	45,92	1,48	AFVEKLGAFWGSIGRF	81,04	3,09
62	VWRLMPGAFWQLRQPA	24,70	0,87	LMGFRDTGILLSIMEV	18,01	0,63	GWWHRSTLCQYGIRFF	45,73	1,47	GWWEKLCVWNAVSTLG	80,88	3,08
63	EWWWYDEMLTWNIHGA	23,76	0,83	LKRHKDQGRLRAIGMF	17,09	0,60	PQMRLKGAFWQSIGRF	45,28	1,46	AEVNWKGAFWDFIERK	80,79	3,08
64	DFRRLPGAFWQLRQPA	23,49	0,82	LPRHDDTVFLDSTRFF	17,07	0,60	YPRHRTYGIYYCNVRF	45,10	1,45	LDRAYFKWCLWMFTMG	80,32	3,06
65	VWRLLAPPFSNRLLSA	23,45	0,82	LPAVRDVTILNSKGRF	16,98	0,60	DPMQRGTGILAFIGRF	44,81	1,44	AEVNWKGAFWSVKKLG	79,88	3,04
66	DFRRLPGAFWQLRQPA	22,94	0,81	LQLERDGGQLLSIGRF	16,81	0,59	LVRHRLRGILKSIFRF	44,02	1,42	LPKFYYTGFLCRYKLG	79,22	3,02
67	ASDWRDTGILLSIGRF	22,92	0,80	LSRHGNAGILDSIGKF	16,45	0,58	LPMQRGDGILFSIYRF	43,76	1,41	KKMFYYTGFLGSIKLG	78,60	2,99
68	VPRHRDTYILDSIGRF	22,92	0,80	ADFHRDTDILDYCYYF	14,98	0,53	YKSEKLCAFWQLRQFA	43,30	1,39	KKFFYYTGFLGSNHGA	77,83	2,97
69	GKWYKGRARPRLNMHM	22,70	0,80	KTRARDTSILDCIGYF	14,90	0,52	LVRHRLRGILDYIGRY	43,29	1,39	TMNKAWTLNFDFIGRF	76,98	2,93
70	GKRLKLCTWTNDGPHA	22,49	0,79	LPRRRDTGSLDNRGRM	14,89	0,52	TEVNWKGAFWELRQPA	42,56	1,37	KKFFYYTGFLGSIGPA	76,75	2,92
71	DFRRNPYSFIKEYHNT	22,45	0,79	LPEHSDGGIVFSFERM	14,88	0,52	KLRVDATRLLDYIGRY	42,44	1,36	GWWEKLSVWKRLNQEA	76,43	2,91
72	LPRHRFCGRLNMHMNS	22,40	0,79	LPRRRFDVTCDSIGRF	14,74	0,52	PQVEKLCAFWQLRCPA	42,26	1,36	AKQRWKAAFWLLRQPA	75,28	2,87
73	GWWYKAPPFSNRLLPG	22,30	0,78	LPLHMDFGQLTSLGRR	14,05	0,49	YPRHRTYGIWQLYHNT	42,21	1,36	LWRLLLKVWGRLSMHM	74,77	2,85
74	YEVNWKTHRLNMHMNV	22,29	0,78	LTRHRQEGILVSIDRL	13,80	0,48	LPRLYDFVILFSIERF	42,07	1,35	KKFFYYTGWLRENMHM	73,87	2,81
75	VHCDILKEVWHVKKLC	22,14	0,78	RVRHFDTGILQSIERL	13,72	0,48	GAMQRYTGIGFSIYRF	42,06	1,35	TINKAWTLNFDFIGRK	73,28	2,79
76	PQIALFCGRLNVIHGA	22,01	0,77	LERHRDTAILLSIGCF	13,66	0,48	DPMQRGTGILFSIYRF	41,92	1,35	MPRHRDTEFWAVSKLG	72,24	2,75
77	HHCSILKSFIEEYVNT	21,85	0,77	LPRHKVTRNLDSNGRG	13,58	0,48	LPRHDYTMILLTYFRF	41,92	1,35	MPRHRLGAFWGSIGRF	70,47	2,68
78	KWWYKGRARPVSAVAA	21,70	0,76	RPRGADTGIYDKIGRF	13,40	0,47	AKVTKLCAFWQLRQRA	41,77	1,34	LDRAKLCLWWSVKKLG	70,42	2,68
79	AKVEKLEMLTWNIHGA	21,64	0,76	EPRHRDFAILDVICAF	13,30	0,47	AEVNWCGLIQYGIRFF	41,14	1,32	GWWHRTYGIYYVNVLR	70,21	2,67
80	LPRHRAMFCGRLNYHM	21,46	0,75	LPLHRDVGYLLYNGSF	12,78	0,45	AEVEWKFMILFSIERY	41,11	1,32	VWRLLLKELGRLNMKM	69,95	2,66
81	LPRSETYSFIKEYHNT	21,45	0,75	LPDHTDTGILYLYGRE	12,75	0,45	MPRHRDTGYLVSILRF	41,01	1,32	VWRLLLKELWEVSKFG	69,61	2,65
82	AEPQIAMFCGRLNMRF	21,32	0,75	NPRVRDTGILSSIGLC	12,68	0,45	YPRHRTYGIYYCNVAF	40,98	1,32	DFQSWKGAFWQLRQGC	69,19	2,64
83	DFRRGPEAFWQLRPHA	21,19	0,74	LPGHRETGILMCINRK	12,51	0,44	LTRENLTGNLCFTYQF	40,71	1,31	MPRRLKQAFWWMFWDN	68,87	2,62
84	QFCRLPGAFWQLTPHA	20,86	0,73	SNRKRFTGINDSIGSF	12,49	0,44	LRVACRTGILAFIGRF	40,60	1,31	LPKFYKGAFWQLRQPA	68,73	2,62
85	SPRHRDTGILDSIGRF	20,77	0,73	LPRHRFTGNLDSDLRF	12,40	0,44	EPRHRDTGYLVSILRF	40,18	1,29	MPSHRDCQWWWNIHGA	68,63	2,61
86	LPRHRDTGILDSIGRF	20,76	0,73	PQIAMFCGRLNMHMNV	12,29	0,43	LVRHRLRGILKSIFRC	39,55	1,27	LDRAYAKWCLRLNLHM	67,83	2,58
87	DFRRWKTHERKVKGQN	20,46	0,72	LQSRRDTGILASERYF	12,22	0,43	LRRRLKRAFWQSIGRF	39,51	1,27	GWWYKGSAFWQVIGRF	67,79	2,58
88	GALSETYSFIKVKKLG	20,39	0,72	LYRHLQDQINDSIGRK	12,09	0,42	LTRMYATLILYSIGRF	38,46	1,24	GWWAYAKWCLWMFTDF	67,77	2,58
89	ASDRLPRVFWQLRQPA	20,38	0,72	LNRMRDLGCLEGRNRS	12,00	0,42	LKRAYAKVILFSIERY	38,37	1,23	LPKRYYTGFLCVSKLG	67,57	2,57
90	RKVEKLCVWNNSIGRF	20,28	0,71	LPRRRDMGLLDSIRVA	11,82	0,41	LRFACRTYILDVIGRG	38,37	1,23	VKVEKLCVWWWNFTDF	66,76	2,54
91	GYWYKGRARPVLAVAA	20,15	0,71	VPRHRQRFILDSQGNF	11,80	0,41	LGYHRATLNFAFIGRF	38,36	1,23	VWRLLLQELWAVSKLG	66,64	2,54
92	YHCSILKEVWHVKKLT	19,97	0,70	LPRHRVTGNTDNICRF	11,46	0,40	GWWYKQTARPWNIFRF	38,02	1,22	AEVEKACVWWSVKKLG	66,44	2,53
93	GALSEVYSFIKEKGSN	19,97	0,70	LPRHRMTGYYDSIGRM	10,86	0,38	DFRRTPKLFWWEYGNA	37,86	1,22	GWAYKGSAFWQVSKLG	66,10	2,52
94	LPRHRDTGILDSIGRF	19,72	0,69	FSRSRDDSKLDSIGRF	10,52	0,37	PQIAAFCAFTQLIYRQ	37,57	1,21	LPVNWKGAFLCSIKLG	66,07	2,52
95	AERHRTTDILDSIGRF	19,30	0,68	LPAGRAADILDSYGRF	10,40	0,37	DFRRLKTLILYSIGRF	36,39	1,17	AKVEKLCVWWTVKKLG	65,25	2,49
96	GALSETYSFIQLRQPA	19,06	0,67	VPVHYDTGILDFITRM	10,24	0,36	RLRVRDFFILKSIFRF	35,34	1,14	LPVNWKCVTWSVKKLG	64,27	2,45
97	DFRQIAMFCGRLNMHM	18,82	0,66	GRMHRDTGIMDVIGRF	10,23	0,36	DFRRLLKEILVLICMF	35,23	1,13	RKVERTYGIYYVMVRF	63,77	2,43
98	DFRRLPEAFWQLRQPQ	18,71	0,66	LPQHRDTGIQLSTARR	10,16	0,36	GKTHRATRVQGLIFRF	34,85	1,12	VWRLLLKAFVQLRGRF	63,63	2,42
99	LPRHRDTGIFNNHMNV	18,51	0,65	QPRHYVTCICDSIGRF	10,15	0,36	LVRHRLRGILKSIFRQ	34,79	1,12	GSWYKGSAFWQYIGRC	63,39	2,42
100	DFRREKYSFIKEKHNT	18,27	0,64	LPRLFDTGDLYEIGTF	9,86	0,35	LPTHGYTMILLTYGRF	34,07	1,10	VWRLLLEAKWAVSKLG	62,98	2,40
101	DQMAMFCGRRNMHMNV	18,17	0,64	LPRARDMGITKSDSRF	9,80	0,34	RWRHDYTMILLTYGRF	33,89	1,09	LPKFYYTGFGALNMHM	62,52	2,38
102	DFRRWKTHEIKVKGQN	18,17	0,64	RPRTLDTGGLVSIGVF	9,37	0,33	DPMQRGTGILFNIYRF	33,64	1,08	VWRLLLKAFWQLEWDN	61,86	2,36
103	DFTRLPGAFWQLRQPM	17,19	0,60	LNFHRDSEQLDSFGRF	9,25	0,32	VWRHRATLNFCFIGRK	33,30	1,07	KKFFYAKWFLWMFTDF	61,35	2,34
104	HHCSIAKEVWQLRQPA	16,55	0,58	LPRHRTTMLFDSIMSF	8,89	0,31	DTRRKLTGILLSTYRF	33,13	1,06	LPVNWLCVWWWEVHGA	61,28	2,33
105	PQIAMFCGRLNKAPHL	16,37	0,57	LSRHMVTGQLDSIGRF	8,69	0,30	LPTFYYTYILDVIGRR	33,09	1,06	GWNEKLCVWGRLNMHM	60,51	2,31
106	HHCHRDTGILDSIGRF	16,35	0,57	LPRVRDQGILESFGMR	8,23	0,29	GALHYDTGYLVSYGRF	32,54	1,05	EDRAYAKWCLWMSTDF	59,81	2,28
107	VWRLLAPPFSNKTPVA	16,29	0,57	VPCHRDTYILDKIRSD	8,18	0,29	DFTHRATVLLDYIGRY	32,46	1,04	AKVEKACVWWSVKKNA	59,67	2,27
108	HHCSILKEVWHVKKNV	16,25	0,57	VDQHSDTFGLDSIGRF	8,17	0,29	DFRRLPKAFWQLYHNE	31,95	1,03	LDRAYAYGIYYVNVTF	59,03	2,25
109	DFRQIAMFCGRLNMHM	16,13	0,57	LPFHRATRILDMIERA	8,16	0,29	GKRHDYTMILLTYGRF	31,79	1,02	MYRHRDTEIYYVNVRF	58,51	2,23
110	ASDWYDEMLTWNIHGS	16,12	0,57	LPRMRDTFIRDSIGKQ	8,11	0,28	LWWYQGRAQPVSAPHF	31,45	1,01	LDRAYAKWCLWVRQYA	58,02	2,21
111	LPRHMFCGRLNMHMNV	15,90	0,56	LDMHRDYGISDSIVRF	7,90	0,28	GWQYKGNAFWQLRQPA	31,44	1,01	DEVNWKGAFWMFIERK	56,96	2,17
112	PQIAMFCGRLNMHMNV	15,78	0,55	AEPQIAMFCGRLNMHM	7,69	0,27	YALSETYSFINRLLPF	31,38	1,01	EKFLKLCVWWSVKKLG	56,69	2,16
113	DFDRIAMFCGRLNMHM	15,66	0,55	LPGHADDKIGCSIGRF	7,67	0,27	LPRLRDFVNFDFIGRG	30,71	0,99	TINKAWTLWTWEYGNA	56,68	2,16
114	APRHRDTGILDSIGLF	15,53	0,55	VPGGEDTAILSSKGRF	7,50	0,26	YPRHRYTGFLCAIGDC	30,53	0,98	KKFFVYTLNFDFIGRK	55,92	2,13
115	HHCSILNEVWHVKKHM	15,06	0,53	LPRHTMTNALDAIGRF	7,26	0,25	DPRLRSTLIQYGIRFF	30,15	0,97	AFVMDLCVWWWNIHGA	55,87	2,13
116	ASDWYDEMLTWNIHGA	15,04	0,53	CMRHRDTGILMSMVRL	7,24	0,25	LPRQVDTGILVVIYRQ	29,94	0,96	VWRLLLKAFCQVKKLG	55,06	2,10
117	HMDSTNKEGWTVKKLG	14,88	0,52	LPRRRDTLVLDSRGAV	7,17	0,25	DPMQGGTLILYSIGRF	29,17	0,94	DKFLKLCVFWQLRWDN	54,34	2,07
118	LGRHRDTGILHVKKLG	14,68	0,52	LSRQADTGALCSIGRL	7,08	0,25	LWWYQGRARPVSAPRC	29,04	0,93	MPRSRDTEVWAVKKLG	54,21	2,07
119	ASDWYDCGRLNMHMNQ	14,64	0,51	LPRHRDTMMLDLIARL	7,01	0,25	LSYHRATLNFDMIFRF	28,70	0,92	GKWHRTYGIYYNIHFA	53,86	2,05
120	PQIACFCGRLNLNMHM	14,35	0,50	LPRHRDRGKLDYIGEF	6,88	0,24	LPRQVDTRILVVIVRF	28,31	0,91	KKFFEKGAFMWMFWDN	53,67	2,04
121	PFVCWKTHEIKVKGQN	14,31	0,50	LCRGRDEGIYDSAFRT	6,84	0,24	LGRHRTYGIYYCNARF	28,30	0,91	KKFFMYTAFWQLRWDN	53,26	2,03
122	LLRRLPGANWQLRQPA	14,26	0,50	MQRLRDTGILGSIGRE	6,78	0,24	DFRDLPKAFWQLKKLG	27,96	0,90	LVKFYYTGFLCSRWDN	53,04	2,02
123	YEVNWKTHEIKCKGHA	14,12	0,50	LMRHRDGGYLDERGRN	6,61	0,23	LSYHRATLNFDFIGGA	27,91	0,90	DEQRWKGAFWQLRQPA	52,90	2,02
124	EQIAMFCVWNNKTPHA	14,11	0,50	TPLHRSTTITDVIGSF	6,26	0,22	GWWYKQTARLKSIQRF	27,67	0,89	EKVEKTGAFWDFIARK	52,47	2,00
125	PQIAMFCERLNMHMSV	13,77	0,48	LPRNRMTGYTDSFERF	6,25	0,22	ELRVRQFFILDGYGRF	27,61	0,89	RWRLLLKELWNVKKLG	51,89	1,98
126	YEVNWKTHEICVKGQN	12,94	0,45	TPRHRYTGCLDSGDRL	6,23	0,22	LVRHVLRGILKVKKLG	27,48	0,88	DFQRAKGAFWAVSKLG	51,22	1,95
127	ASDWRDTEILDSIGRF	12,81	0,45	VNDHRDTGMAYVIGRG	5,46	0,19	DPMQRGTGILFVIGRF	26,97	0,87	EKVKKLGAFWGSIGDN	50,50	1,92
128	AEPQIAMFFIKEYHNT	12,81	0,45	LNRSRDTGILAANMRQ	5,15	0,18	LPRRLPKAFFQLYHNT	26,84	0,86	GWWYKGSAFWQVIGPT	50,17	1,91
129	CQNRMSCVRLNMHMVV	12,78	0,45	LRRHRETGFLDEAKQF	5,14	0,18	FPRHRNFAFWQLRQPA	26,71	0,86	VWVRKLCVWYWNIHGA	50,12	1,91
130	VWLSETPSFSKEYHNT	12,71	0,45	ACGHDDTGSLNSIGRY	5,00	0,18	GALRLLKEVWHVNKEG	25,32	0,81	DWFLKLCVRWSVKGDC	49,76	1,90
131	YPDHRDTGILDSIGRF	12,50	0,44	LPFTRDFGDDLSIGSF	4,91	0,17	YPRHRTYGIYVSIQSF	25,31	0,81	EKVEKCCVWGRLNMHM	49,17	1,87
132	AEPQIDEMLTWNIHVA	12,34	0,43	CPFECDTRALDSIGRF	4,90	0,17	MPMQRGTGILDSIYRF	24,42	0,79	TEVNWKFASYDFIAFK	49,01	1,87
133	PQIAWKTHEIKVKGQN	12,13	0,43	RPRCQDTGILKSICEF	4,78	0,17	GRTHRATRLLDYIGGK	24,33	0,78	DFQRLLKELWNVKKLG	48,60	1,85
134	DFRRQKDAFWQVRQCA	11,41	0,40	LMEVFDTGITDSIGRE	4,55	0,16	GKTHRATGILVVITRQ	24,26	0,78	VFVEKACVWWSVEKLG	46,96	1,79
135	GTWYKGRARPVSANAA	11,27	0,40	GPRHGDLGIKDSIGRV	4,45	0,16	LVRHAPKAFWQLYHNT	23,71	0,76	GWEVKLCLRGVLNDHM	46,85	1,78
136	LPRHRDCGRLNMHMNV	11,16	0,39	LPKHGQTGIVGSMGRM	4,45	0,16	LTRRNDTGYLVSIGRS	23,51	0,76	LPKFYYKGFLCSIKLK	46,56	1,77
137	CYVNLMTHMIYVKGFN	10,99	0,39	FPRHKDTGGLDYVGKS	4,45	0,16	YKRLLLKELWHVKKLG	23,43	0,75	LDRAYAKWWTWEYGNA	46,27	1,76
138	LPRHRDTTCGRLNMHM	10,77	0,38	YPQKRDTGGCDSISRF	4,41	0,15	LMRACRTYILDVIGRC	23,15	0,74	AKVEKLCVQWYVNVRF	46,01	1,75
139	ASDWYDEMLTVNIHLN	10,58	0,37	KDRHRDSVILDSGGRL	4,37	0,15	LPTFYYTGKLCSAPHA	22,84	0,73	GGWYKGSGFLCSIKLG	45,82	1,75
140	ASDWYDEMLDWNIHNV	10,50	0,37	LPNHRDKVRADSCGQF	4,23	0,15	MPRHVDTRLLDYIGRY	22,82	0,73	CINKAWTLNFDFRQPG	45,81	1,75
141	LPRHRDTGIIKEYHNT	10,35	0,36	LPRHRDMRILDNIERD	4,22	0,15	LDRAYAKELWHVKKLF	22,21	0,71	AKRLLLKELWEVKKLG	45,46	1,73
142	LPRHRDTGIIKVKGQN	10,29	0,36	LPRHRDTGDSLSIGKV	4,10	0,14	LPRHCATLNFDFIGRK	21,25	0,68	GWFFYYTGFLGSIGDC	45,29	1,73
143	GPRCRDTGRPVSAVRF	10,25	0,36	LELHRKTGINDSIGSF	4,02	0,14	PQIAMFCAFWQQDLPA	20,34	0,65	GWTYKSSAFWAVSKLG	45,11	1,72
144	AEPQIARFCGRLNGGF	9,76	0,34	LPRHAELGLLKSEGRV	3,97	0,14	LVRHRLRGILKSTKLG	20,19	0,65	VWRLLLKELCGSIGDC	44,37	1,69
145	AEPQIALFCGRMHTNV	9,66	0,34	LAVHRDTGALDSNCRF	3,91	0,14	DPRHRNYGVQGLIFRC	19,83	0,64	GWWYKGSAFWQVITDF	44,36	1,69
146	LPRHRDTGILNMKMKV	9,45	0,33	LPEHRVTGIQDNIYRS	3,91	0,14	LPTYKGTAFWKLRQPG	19,67	0,63	DFERLVGAFWDLRQPG	44,08	1,68
147	PQIAMFCPFSNRLLPA	9,36	0,33	VPRHSDTGFLDTQTRF	3,84	0,13	GALDRATLNFDFIGRK	19,47	0,63	AWWEKLCNRGRLKMCR	43,57	1,66
148	HHCSILPNFSNRLLPA	9,34	0,33	LPRHRMTGITDTEGRF	3,83	0,13	LPRVKCTGILANIGRF	19,21	0,62	AKVEKAAVWWSVKGRC	43,48	1,66
149	PQIAMMCGRLNMHQPA	9,30	0,33	LVFHRDMGTLDMIGAF	3,69	0,13	AKVEKKGATWQSIGRF	18,83	0,61	EKVEKLGAFWNSIGRM	43,46	1,66
150	AKDEKVCTWFCGTPSQ	8,76	0,31	LPRHRDTGECDRLCAF	3,63	0,13	LPRLRDKVILWMSWDN	18,58	0,60	MPRHRDTVWGRLNDHM	43,12	1,64
151	DVRRLPGAFWQSAVAA	8,50	0,30	LPRSLKTQILESIGEQ	3,60	0,13	LGYHRATLNFDVIGRK	18,44	0,59	DRRCLMYAVWWMFCDN	43,09	1,64

152	YFRRTPGASALCQQPA	8,45	0,30	RPTHFDTAGLDSTLRF	3,50	0,12	LPRLRDFVILKSIGVF	18,19	0,58	AWWHRTYGIYYVNDRF	42,54	1,62
153	GALWYDDMLTWNIAGA	8,42	0,30	LPRCRDQCFLDSSGGF	3,47	0,12	DFRRVLKEVWHVNERY	17,86	0,57	DFQRWKGAFRQLRQPF	41,78	1,59
154	GAKSETYSFIKEAVVA	8,41	0,30	LVDHSLTKKLDSECRF	3,31	0,12	KKFTRDTGILRLRQRA	17,64	0,57	TFQRWKGLNFDFIQML	39,34	1,50
155	AEPQTAMAFWQSRQPA	8,13	0,29	TPRHRDTMSLDSCYRQ	3,30	0,12	GGYHRATLNFQRLLPA	16,86	0,54	ALVNTYKAFLCFIRRK	39,25	1,50
156	YSDWYDEMLTWNIQGA	7,91	0,28	LARHLDTGILDKIERT	3,29	0,12	LPRHDYTMILLTIERY	16,66	0,54	GWYNKLCVWGRLNMNA	39,14	1,49
157	YEVNWPGAFTQLRQPA	7,89	0,28	QPKHRDTGCLDQLGRT	3,28	0,11	DPMQRGTLILFMFWDN	16,46	0,53	LQVMWKAAFGQLRQSA	38,74	1,48
158	LPRHRDTGILDLNMHN	7,65	0,27	LPRCLDTGMYDSIGCF	3,21	0,11	KDYVRKGAFWDGYGRA	16,38	0,53	DWFLAWTLNDDFIGRK	36,64	1,40
159	AEPQMFCGRLNMHMNV	7,27	0,26	YQRHQDDGILDSICAF	3,15	0,11	GWQYKGTACWQLRQRA	16,31	0,52	EKVEKLGAFWAVSGLG	36,35	1,38
160	SPRHRDTGILDSLLPA	7,23	0,25	LGMERDTGGLDAGGRF	3,05	0,11	KKFDRDTGILKSIGLG	16,23	0,52	LDWEKLCVWERDNMHM	35,44	1,35
161	DFRRLPTHEIKVKGQN	6,47	0,23	LPRGRATGALDENGKF	3,04	0,11	LTRRNLKEVWHVNKLG	15,97	0,51	GWWEELTLNFDFIGRK	34,95	1,33
162	LPRLSDTDIYTSGGRF	6,38	0,22	APRHFMTGNNDSEGRF	2,80	0,10	LGRHRTYGQYYCNVRF	15,73	0,51	LFVMKLCVWTGSIGRF	34,31	1,31
163	MKVMKLCERNNKTPEA	6,21	0,22	NGRCRCTEIDRDIGAF	2,78	0,10	MPREKLCKFWQLRQPA	15,47	0,50	GWWHRTTGIWQRRQPG	33,97	1,29
164	LPRHRDTGILDSIHGA	6,20	0,22	LKEHRDTGRLDLDGAF	2,70	0,09	LERLRDFVILFSIEDC	15,45	0,50	MPRHRGSAFWQVSGLC	33,37	1,27
165	DFRRNPGHEIKVKGQN	5,96	0,21	LRRRMDQGNLDSIEFA	2,58	0,09	DLRHRNCYVQGLIKLG	15,39	0,49	MPRLLLKAFWRLRQPG	32,91	1,25
166	PQIYKGRARPVSAVAM	5,95	0,21	LPYHRDVGFDDQTSRF	2,53	0,09	KLRFRDTGYLVSGGRF	15,10	0,49	MPRHRDTVWARLNMHM	31,78	1,21
167	PSKYYDEMLEWNIHGA	5,94	0,21	GPREFDTGLLDSNRKN	2,52	0,09	YPRHRGTGISVLIGMF	14,80	0,48	YKVEKLCVWGRENMHM	31,67	1,21
168	GWAEKKRARPVSAVNC	5,93	0,21	LPQHRDTEILESFEDF	2,51	0,09	LDRVRDFFILDGYGRT	14,59	0,47	DPQHKLCVWGRLNMHM	31,51	1,20
169	HFESILMEVRHEKELG	5,55	0,19	LPRQKDTGIYDSMQRF	2,47	0,09	LPRHDYTMILKTYGRF	14,11	0,45	TINKAWTLNFDFIGDN	31,41	1,20
170	LPRHADTGILDSIGLG	5,42	0,19	LPRHTDYGINDTIGRM	2,25	0,08	AKVEKLDAFWQLRQPY	14,02	0,45	VWRHRDTEVWAVSLLG	30,51	1,16
171	QFTRLPGAATATRCVA	5,31	0,19	LPRHTMCNIMDSIGSF	2,15	0,08	LTRRNLTGILSSTYRQ	13,35	0,43	VKVLLLKELWSVLKLG	30,49	1,16
172	LKNHRDAGINDSIGRG	5,23	0,18	LPRRRDTGILDSDQQQ	2,15	0,08	LWQYQGRARTVFAPRA	13,28	0,43	MPREKLCVWVRLNMHM	30,34	1,16
173	YEPQIAMFCGCLNMHM	4,77	0,17	VPRGRDAGILDSNGSF	2,14	0,07	DFRRLPGAFWQLRQPA	13,04	0,42	VRRLLLKAFWQLRQPG	29,72	1,13
174	DDRRLPGAFKVSAVAA	4,62	0,16	LTRARDDGDMDSAGRF	2,11	0,07	DFKRLSGAFQQSKGRF	12,15	0,39	GKWEKLGKWGSLCDQV	27,73	1,06
175	LPRHRQTGILDSIMNV	4,53	0,16	LPQETDKQYLDSIGQF	2,02	0,07	YPQHMTYGIYYCRQPG	11,90	0,38	LDRAYAKWCWANSKLG	27,63	1,05
176	LEPQIAMFCGRLQMHM	3,88	0,14	DRRHGDQGMLDMIGGF	1,96	0,07	GFRRLGRARPVSAVHA	11,56	0,37	EKVEKLGAFWGLNMKM	26,70	1,02
177	LPIHRDTGILNMHMNV	3,80	0,13	LCGVRDTGMNDSSGGF	1,91	0,07	LPRHRSTLIQMTYSRF	10,31	0,33	NKVEALYGFLGSIGDC	26,01	0,99
178	AEPLLAPPFMNRLLPA	3,70	0,13	LPFHEDTGIQDMITRF	1,82	0,06	LRFACGEARPVSAPHA	10,02	0,32	GEVNWRGAFEDFIERK	25,81	0,98
179	LPRHKDTGILDSDGRF	3,59	0,13	LPRHRDGEILESIGNM	1,74	0,06	DQRRLPTYILDVIGRC	9,92	0,32	AEVNWKGAFWDFNQEA	25,69	0,98
180	GALSETYSFPVSAVAA	2,93	0,10	LPDLRRTGIDDSNGMF	1,73	0,06	LPRHRSTLIQYGIPHS	9,77	0,31	DFLTYKSDSWQLRCYA	25,63	0,98
181	AEPQDDMFCGRLNMHM	2,78	0,10	LDRVRDTGILDSKGQS	1,72	0,06	KKFDRDTGILKSIGFQ	9,60	0,31	SEVRWKGGFLGSIGDC	23,89	0,91
182	ASDWKDTHEIKVRGQN	2,69	0,09	DPDHDDTGYLDEIGRL	1,72	0,06	RVRHADTGYLNSIGRF	9,25	0,30	AKVAKLCELWNVKKLG	22,98	0,88
183	HECSILKERPVSAYAC	2,54	0,09	TSQHRDTGQRDDTGRF	1,67	0,06	DFRRRDFNILDGYERF	9,24	0,30	DCWYKGSADWQVIGRC	21,22	0,81
184	HHCSRLKEVIDVKGQN	2,33	0,08	LERHRDTGLADSIGYD	1,66	0,06	LTRRNLTGIAYPWLTQ	9,05	0,29	MMRHRDTEVWAVSKRF	20,05	0,76
185	AEPQIAMFCGNMHMND	2,26	0,08	LPRQRDTQIQDCKGQA	1,65	0,06	AKVEALCAFSQLRQPA	8,97	0,29	MPNKAMTLNFDFIGRK	16,63	0,63
186	AERHRGTGICTSIGNQ	2,22	0,08	NGCHRDTGILDLQGSV	1,58	0,06	LLRVRDFNILDGYGRM	8,92	0,29	DFQRKLCVWGRLNMGM	15,41	0,59
187	LPVHRDTGEIKVKGQN	2,15	0,08	LDQVRDKGSEDSINRF	1,56	0,05	YPDHRTAVWGRLNMHM	8,62	0,28	TINKAWTNNFSVKKLG	14,22	0,54
188	AEPEIAMFCGDLNMHM	1,76	0,06	LPTHRDMGIACSEMMF	1,44	0,05	LPRLRATTNFDGIGRK	8,46	0,27	AEVNWKGAGWQLRQEA	11,41	0,43
189	AEPQIGRARPVSAVAA	1,74	0,06	LPRLVDTTALVSMGDF	1,40	0,05	LPRHRDTGILDSIGRF	8,13	0,26	AFVMKLCVMTWEYGNF	11,01	0,42
190	LPRHTDTGILDSIGQN	1,57	0,06	LPRHRDCNEQDSIAEF	1,20	0,04	LGYHRATLNTWEYGNA	8,05	0,26	TFQKAWTLNQCLIGAK	9,24	0,35
191	GALSETYARPVSAVAT	1,54	0,05	CPLHRDTRELDSVRDT	1,16	0,04	DPRHRDTGSLVSIGRF	7,73	0,25	GWMEKMCVWYSLTETM	9,17	0,35
192	ASDWYDEMLTNRKLPA	1,50	0,05	LPFDRDTGTLKDIGSD	1,08	0,04	DPMQRGTGFINRLLPA	7,33	0,24	TINHRATEVWAVSGLG	8,49	0,32
193	PQIAMFCGEPVSAVAA	1,38	0,05	LPKERNTGDLKSIVDM	1,01	0,04	GAKSETYARPVNIHGA	6,81	0,22	AELQQKGAVLDFVSRK	6,86	0,26
194	LPRHRDTGMLDSISQN	1,29	0,05	EPRHRDDMILESMTCF	0,93	0,03	TINAAFDNWARPWCEL	6,23	0,20	MPNHRDTAFWQMRQPG	5,53	0,21
195	PDKAMFCGQLNMSCNV	0,96	0,03	CPRDRCTGDLDSIDRM	0,91	0,03	TPRHRDTGYLQLRQPG	5,90	0,19	DFERLKCTRTWEYGNA	5,31	0,20
196	PQDAMFQNDLAMHKTV	0,68	0,02	LPEHQDTGEKESINRM	0,71	0,02	LPAQRDTGIAVVIYPG	5,89	0,19	MPRHGLCVGGAVSKLM	5,09	0,19
197							AANAMWDNWDYPWMMK	4,01	0,13	MPRHRDTEVWNVKQLG	2,27	0,09
198							LQRHAKTEELYLIGRM	2,69	0,09	MPRHSDTEVGRLNMHM	1,92	0,07
199							MPRHRDKGMDVSVGES	1,41	0,05	MPVHRDTELMNVKKLG	1,63	0,06
200							LPRQDDTGFLCSIGDC	0,73	0,02	MPRYRDTEVWAVSGDC	0,11	0,00
	**Lead Peptides**			**Lead Peptides**			**Lead from Gen1**			**Lead from Gen2**		
	HHCSILKEVWHVKKLG	31,19	1,09	YEVNWKTHEIKVKGQN	34,56	1,21	YPRHRTYGIYYCNVRF	71,89	2,31	GWWEKLCVWGRLNMHM	171,88	6,55
	PQIAMFCGRLNMHMNV	29,09	1,02	GALSETYSFIKEYHNT	33,69	1,18	DPMQRGTGILFSIYRF	67,81	2,18	AKVEKLCVWWWNIHGA	101,63	3,87
	DFRRLPGAFWQLRQPA	24,00	0,84	GWWYKGRARPVSAVAA	33,13	1,16	LVRHRLRGILKSIFRF	64,09	2,06	EKVEKLGAFWGSIGRF	96,78	3,69
	GWWYKGRARPVSAVAA	23,14	0,81	DFRRLPGAFWQLRQPA	27,23	0,96	MPRHRDTGYLVSIGRF	63,98	2,06	LDRAYAKWCLWMFTDF	94,03	3,58
	YEVNWKTHEIKVKGQN	21,60	0,76	HHCSILKEVWHVKKLG	25,91	0,91	LGYHRATLNFDFIGRK	63,02	2,03	SEVRWKGAFWQLRWDN	89,86	3,42
	LPRHRDTGILDSIGRF	19,28	0,68	VWRLLAPPFSNRLLPA	23,53	0,83	GWWYKGTAFWQLRQPG	61,99	1,99	TINKAWTLNFDFIGRK	81,48	3,10
	AEPQIAMFCGRLNMHM	19,21	0,67	AKVEKLCVWNNKTPHA	19,84	0,70	DFRRLKGAFWQSIGRF	59,08	1,90	DFQRWKGAFWQLRQPA	81,27	3,10
	AKVEKLCVWNNKTPHA	18,02	0,63	LPRHRDTGILDSIGRF	19,81	0,70	AEVNWKGAFWQLRQPA	55,82	1,79	DWFLKLCVWWSVKKLG	81,17	3,09
	GALSETYSFIKEYHNT	15,06	0,53	ASDWYDEMLTWNIHGA	15,10	0,53	GKTHRATRLLDYIGRY	54,91	1,77	MPRHRDTEVWAVSKLG	80,08	3,05
	ASDWYDEMLTWNIHGA	14,96	0,53	PQIAMFCGRLNMHMNV	11,55	0,41	LPTFYYTGFLCSIGDC	54,70	1,76	KKFFYYTGFLCSIGDC	77,72	2,96
	VWRLLAPPFSNRLLPA	9,85	0,35	AEPQIAMFCGRLNMHM	7,44	0,26	VWRLLLKELWHVKKLG	54,15	1,74	GDRAYGKWCLWMFWDN	76,78	2,93
							LPRLRDFVILFSIERY	53,94	1,73	VWRLLLKAFWQLRQPG	74,88	2,85
							LRFACRTYILDVIGRC	53,68	1,73	GWWHRTYGIYYVNVRF	72,14	2,75
							AKVEKLCVWWSVKKLG	52,95	1,70	AEVNWKGAFWDFIERK	72,08	2,75
							LPRQRDTGILVVIYRQ	51,40	1,65	DFERLKGAFWWMFWDN	71,37	2,72
							PQIAMFCAFWQLRQRA	51,37	1,65	VWRLLLKELWNVKKLG	67,24	2,56
							LPRHDYTMILLTYGRF	50,36	1,62	LPKFYYTGFLCSIKLG	67,17	2,56
							AKVEKLCAFWQLRQPA	50,26	1,62	LPVNWKGAFWQLRQEA	66,30	2,53
							DFRRLLKEVWHVNKLG	48,30	1,55	AKVEKACVWWSVKKLG	65,15	2,48
							GWWYKGTGILVLIGMF	47,66	1,53	DFRRLKGAILFSIYRF	64,85	2,47
							LPRVKNTGILAFIGRF	46,91	1,51	GWWYKGSAFWQVIGRC	54,79	2,09
							LTRRNLTGILSSTYRF	46,19	1,48	AFVMKLCVWTWEYGNA	53,52	2,04
							GWWYKGTARPWNIHGA	45,44	1,46			

	Generation 3b	x	c	Generation 4	x	c	Generation 5	x	c	Generation6	x	c
1	**GWWEKLCGWGRLNMHG**	341,01	12,99	**GWWEKLFNWGRQNQPA**	357,46	20,95	**GWWEKLFNWGRQNQPA**	290,53	23,02	**GWWEKLFNWGRQDQPK**	103,90	26,88
2	**GWFEKLCDWGRLNMHM**	313,01	11,93	**GWWEKLCGFWNFCERK**	175,29	10,27	**GWWEKLFNWGRQNGHG**	263,61	20,88	**GWWEKLFNLGCQNQPM**	99,93	25,86
3	**GWWEKLCVWGRLNMHM**	221,02	8,42	**GWWEKLCEWGRLNMHG**	165,94	9,72	**GWWEKLFNWGRSNMHM**	259,20	20,53	**GWWEKLFNWGRQQQTA**	94,28	24,39
4	**DFQVWAGAFWQLRQPG**	172,42	6,57	**GWWEKLCGWGRLNMHG**	152,39	8,93	**GWWEKLFNWGRQNQPA**	252,53	20,01	**GWWEKLFNWGRQDQPK**	91,01	23,55
5	**GWWEKLCVWGLLNMHM**	169,72	6,47	**CWRELLKAWWDFCARK**	151,59	8,88	**GWWEKLFNWGRQFQPA**	246,17	19,50	**QWWEKLFNWGRQNQPA**	90,70	23,47
6	**GWWEKLCTWWWQIHGA**	168,39	6,42	**GWWEKLSAWWWNIHGA**	149,39	8,75	**GWWEKLFNWGRQNQPA**	233,94	18,53	**CWWEKLFNWGRQNGCG**	89,95	23,27
7	**MPRHRDTEVWTVSKLG**	164,07	6,25	**GWWEKLCGWGALNMHM**	143,28	8,40	**GWWEKLFNWGRQNQPA**	224,81	17,81	**GWWEKLFNWGRQNQPA**	89,23	23,09
8	**GWWEKLGAFWDFICRK**	141,30	5,38	**MPRFRDQEVWTVSKLG**	143,16	8,39	**GWWEKLFNFWSNRQPA**	212,03	16,80	**GWWEKLFNWGMQNGHG**	88,10	22,79
9	**GWWEKLCVWGRLNMPA**	135,26	5,15	**GWWEKLCGWGRFNMHM**	140,64	8,24	**GWWEKLFNWGMLNMHG**	210,08	16,64	**GWWEKLFNWGRQEQPA**	88,02	22,77
10	**GWWEKLCVWWWNIHGC**	133,01	5,07	**GWWEKLCVWGRQNMHM**	139,72	8,19	**GWWEKLFNWGGNIHGA**	196,24	15,55	**GWWEKLFNWGRDNGHG**	86,26	22,32
11	**GWCEKLCVWGRLNMHF**	125,82	4,79	**GWWEKLCAWGRLNMHM**	139,40	8,17	**GWWEKLFNWGRQDQPK**	192,83	15,28	**GWWEKLFNWGMLNMHG**	85,95	22,24
12	AQSEKCCVWWQVKQLG	125,75	4,79	**GWWEKLCSWGRLNMHG**	136,64	8,01	**GWWEKLFNWGRQNQLG**	189,74	15,03	**GWWEKLFNWGMLNMGG**	85,74	22,19
13	GWWEKLCVWGDFIERK	111,89	4,26	**SWWEKLGAFWQLRQPG**	136,63	8,01	**CWRELLKAWWRDNCAM**	188,39	14,92	**GWWEKLFNWGRSNMHM**	82,14	21,25
14	DFQRWKCVWGRLNMHM	111,01	4,23	**GWWEKLCGWGRQNMHM**	135,71	7,95	**GWWEKLFNWGRQEQPA**	183,92	14,57	**GWWEKLFNWGRQFQPA**	82,10	21,24
15	TINKAWTEVWAVSKLG	109,80	4,18	**KWWGYLCFWCRLNYRQ**	132,83	7,78	**GWWEKLFNLGRQNQPM**	176,63	13,99	**GWWEKLFNWGNLNMHG**	81,93	21,20
16	GWWTWKGAFWDFIERK	107,97	4,11	**GWWEKLCAFWSNIHGC**	131,56	7,71	**SWWEKLANWGRNNQPA**	170,12	13,48	**SWWEKLFNWVGQNQPA**	81,14	20,99
17	EWWEKLCVCGRLNMDN	107,77	4,11	**GWWEKLCVWGRQNMHM**	131,23	7,69	**GWWEKLFNWVRQNQPA**	169,20	13,40	**GWWEKLFNWGREDQPK**	79,02	20,45
18	GWWEKLCVWWSTRKLG	107,29	4,09	**GWWEKLCVWGRQNCHM**	130,99	7,68	**GWWEKLFNWQRQNMHM**	168,84	13,38	**GWWEKLFNWGRQNGHG**	77,20	19,98

19	EKVEKLCVWWWNIGRC	106,32	4,05	**GWWHRDSEVWQVSKLG**	130,74	7,66	**GWWEKLFNWGRQNQPA**	166,10	13,16	**GWWEKFFNWGMLNMHG**	76,85	19,88
20	DWWHRTDGIYYVNVRF	105,59	4,02	**GWFEVSCNWCKLNMMM**	130,26	7,63	**GWWEKLFNWGRQNQGA**	163,74	12,97	**GWWEKLFNWGRQNGHG**	75,17	19,45
21	QWWEFLCVWGRLNMHM	104,94	4,00	**VWRELLKAWGRFNMHM**	130,17	7,63	**GWWEKLFNWLRQNQPA**	163,48	12,95	**SWWEKLFNWGRQNQPA**	74,66	19,32
22	KKFFYYTGFLRLNMHY	104,39	3,98	**GWWEKLCGWGRLNMHM**	129,50	7,59	**GWLEKLCGFWNFNMHM**	163,41	12,94	**GWWEKLFNWGCSNMHA**	73,71	19,07
23	GWWEKLCVWGRCNVAF	104,20	3,97	**KWWEKLCAWWWNIHGA**	128,32	7,52	**GWWEKLFNWGRQNMHG**	157,74	12,50	**GWWEKLFNWGRQEQPA**	73,34	18,98
24	SEVRWCGAFWGSIGRF	104,13	3,97	**CWWEKLCGWGRLNMHM**	127,90	7,49	**GWWEKLFGWGRQNMHF**	154,06	12,20	**GWWEKLFNWGRQNQPA**	73,17	18,93
25	KKFFYYCVWWSVKELG	101,62	3,87	**GWWEKLCTWWWQIHCA**	122,85	7,20	**GWWEKLFNAGRQNHCA**	151,50	12,00	**GWWEKLFNWGMLNNHG**	72,76	18,83
26	LDRAYATEVWAVSKLD	101,02	3,85	GWWEKLCDWGRLNMYM	121,81	7,14	GYWEKLFNWGRQNQPA	151,36	11,99	GWWEKLFNWGRQNQPA	72,31	18,71
27	GWWEKLCVWGSYIGRY	100,75	3,84	GWWEKLGAFGRLNMHM	121,61	7,13	GWWEKLASWGRQNCHM	150,58	11,93	GWWEKLFNWGRSNMHM	72,21	18,68
28	YPQRWKGAFWQLRQPA	100,24	3,82	GWWEKLGAFWDFTCRK	118,85	6,96	SWWEKLFNWGRQNQPA	147,41	11,68	GWWEKLFNWGMLNMHM	72,08	18,65
29	MPRHRDTEVWAVSKLG	99,39	3,79	GWWEKLCVWGRQNMHM	118,39	6,94	AWWEKYFNWGRQNQPM	141,14	11,18	SWWEKLFGWGRQNMHF	71,76	18,57
30	DFRARDTEVWAVSKLG	99,25	3,78	GWWEKLCGWGRLNAHA	118,34	6,93	GWWEKLKAWWDFCARK	139,80	11,07	GWWEKLFNWGRQNGHG	71,33	18,46
31	DFERLKGAFWWLRQPA	99,08	3,77	GWWEKLCVWWRQNQPR	116,80	6,84	GWWEKLMNWGRLNMHG	138,23	10,95	GWWEKLFNWGRQNQPA	71,03	18,38
32	AEVNWKGAFWDFIERK	98,36	3,75	GWWEKLCVWGRQNMHK	116,78	6,84	GWWEKLCEWGRLNMHG	137,03	10,86	GWWEKLFNWGRSNMHM	70,88	18,34
33	EKVEKLGAFWWNIHGA	97,28	3,71	EWWEKLCVWGRQNQPA	116,53	6,83	GWWEKLCEWGRQAMHM	132,76	10,52	CWWEKLFNWGMLNMHG	70,86	18,33
34	MPRHRDTEVWAVSKLG	96,45	3,67	GWWEKLCGWGRLNFHM	115,62	6,78	GWWEKLGNWGRFNMHG	128,49	10,18	GWWEKLFNWVRQNQPA	70,78	18,31
35	AKVEKLCVWWWNIHGA	96,37	3,67	GWWEKLCGWGRLNMHG	115,26	6,75	GWWHRDQEVWTVSKVG	127,71	10,12	GWWEKLFNWGRQFQPA	70,62	18,27
36	LRRAYDTEVWAVSKLG	95,13	3,62	GWWEKLCVWGRMNMHM	114,59	6,71	GWWEKYFNWGRQNQPA	127,64	10,11	GWWEKLFGWGRQNMHG	70,59	18,26
37	MPRHRDTEVWAVSKLG	95,08	3,62	GWWEKLCVWGRLNMHM	114,07	6,68	GWWEKLKNWGRQNQPA	127,20	10,08	GWWEKLFNWNRSNMHM	70,32	18,20
38	MNRHRDTEVWAVSKLG	94,54	3,60	GWWEKLCTWWWQIHGA	113,85	6,67	GWWEKLCEWGRLNMHG	126,34	10,01	GWWEKLFNFGRQNMHG	70,31	18,19
39	VWRLLDKAWGRLQMHM	93,74	3,57	GWWEKLCVWGRQNMHM	113,51	6,65	GWWEKLCEWGRLNMHG	125,26	9,92	GWWEKLFNWGRQNQGA	70,12	18,14
40	VFERLKGNFWWMFNDN	93,16	3,55	GWWEKLCVWGRQNQPA	113,22	6,63	GWWEKLCEWGRLNMHG	125,18	9,92	GWWEKLFNWGRQNGHG	69,57	18,00
41	GWWEKLCGWGFFIYRF	92,06	3,51	GWFEKLCDWGRLNMHM	112,49	6,59	MWWKLLKAWWDFCARK	125,13	9,91	GWWEKLFNWGRQNQPA	69,11	17,88
42	SEWEKLCVWGRMNMHM	91,45	3,48	GWWEYLCKWGRLNMHM	112,35	6,58	NPRFRDQEVWTVSKLG	124,73	9,88	GWWEKLFNWYMLNMHG	69,03	17,86
43	AEVTKLCVWDRFNMHM	89,61	3,41	GWWEKLCVWGRLFQPA	111,97	6,56	RWWEKLFNWMLQNQPC	124,08	9,83	GWWEKLFNWLRQFQPA	68,66	17,76
44	DKVEKQCVWWWNIHGA	88,59	3,38	FWWEKLGAFWQMRQPG	111,18	6,51	GWWEKLCEVWQVSKLS	123,07	9,75	GWWEKLFNWGRQNMHG	68,28	17,67
45	EKVEKLCVWKWNIHGA	88,43	3,37	GWWEKLCKWGRLNDPA	111,07	6,51	GGWEKLFNWGRQIHGC	122,62	9,71	GWWEKLFNWGRSQMHV	66,63	17,24
46	AEVNWKGAFWQLRCEA	88,25	3,36	GWCHRDSEVWAVSKLG	110,02	6,45	GWWEKLFNWARQNRGA	122,09	9,67	GWWEKLFNWGRQNQPA	66,51	17,21
47	KKGFYYTGFLGSIKLG	88,11	3,36	GWWEKLCVWGRLNMHM	109,30	6,40	GWWETLKAWWDFCARK	121,80	9,65	GWWEKLFNWGMLNCAM	64,21	16,61
48	GWWQLLKAGWQLRQPK	87,66	3,34	GWWEKLCVWGRLNMHM	108,99	6,39	GWWEKLCEWGRLNMHG	120,88	9,58	GWWEKLFNWGRQFQPA	64,17	16,60
49	LPVEKLCVWWWGIHQA	86,32	3,29	GWWEKLCVWGRQNQNA	108,93	6,38	GWWEKLCTWWNSCERK	120,75	9,57	GWWEKLFNWGRSNMHM	64,17	16,60
50	GWWEKGTMLLDYIGRY	86,30	3,29	VWRELLKAWGRLFMHM	107,25	6,28	GWWEKLFAWNRQNQPA	119,04	9,43	GWWEKLFNWGGNIHGA	62,39	16,14
51	GWWHKLCVWGVLNMHM	85,76	3,27	GWFEKLCGWTRNNMHG	104,98	6,15	DWWEKLSAWWWNIHGA	117,89	9,34	GWWEKLFNWGRQNVPA	62,21	16,10
52	VWRLLLKMFGRLNMDM	83,58	3,18	GWFEKLCDWMRLNMHG	104,90	6,15	CWRELLKAWWDFCARK	115,19	9,13	GWWEKLFAWWRDNCAM	62,03	16,05
53	AEVNWKGAFWWMFWDN	82,80	3,15	GQFGKLCEVWAVSKLG	104,52	6,12	GWWEKLCGFWNFCERK	114,95	9,11	GWWEKLFNWGRQNQPA	61,43	15,89
54	VWRLLFKAFWQLRWDN	82,59	3,15	GWWEKLCVWGRLNMHN	104,16	6,10	SWWEKLGAWGRQNTHM	114,84	9,10	GWWEKLFNWLRQNQPA	61,36	15,88
55	GWWHRTYRFWGSIGRY	81,81	3,12	GWWEKLCVWGDLNMHG	104,13	6,10	GWWEKLKAWGRQNMHM	114,12	9,04	GWWEKLFNFWSNRQPA	60,75	15,72
56	MPRKRDTEVWAVSKLG	81,81	3,12	GWWEKLCVWGQLRMHM	104,10	6,10	GWWEKLCVWGRQNMHM	113,58	9,00	AWWEKLFNWGRENMHM	60,37	15,62
57	LPKFYYTGVWAVSKLG	81,15	3,09	GWWEKLCVWGELNMHM	103,75	6,08	GWWEKLNAFWSNIHGC	111,98	8,87	GWWEKLFNWGRQDQPA	60,27	15,59
58	LWRLLLKELWNVKGLG	80,51	3,07	GWWEKLCVWFRLNMHG	102,74	6,02	GWWNKLSAWWWNNMHM	111,01	8,79	GWWEKLFNWGGNIHGA	58,87	15,23
59	VWRRWKLAFWQLRWDN	79,56	3,03	GWWEKLGVWGRLNMHM	102,53	6,01	CWRELLKLWWDFCAKA	110,90	8,79	GWWEKLFNWGRQEQPA	58,46	15,13
60	GDRAMGKWCLWMFWDF	79,46	3,03	GWWEKLCVWGRANMHG	101,36	5,94	GWWEKLCGWGRQNMHM	108,47	8,59	GWWEKLFNFWSNRQPA	58,37	15,10
61	LPVNWTGAFWDFIERK	78,33	2,98	GWWEKLCVWGRLNMHM	101,29	5,94	GWWEKLCVWGRQNMHM	106,98	8,47	AWWEKYFNWVRQDQPK	57,95	14,99
62	VWRLLLKFFWQLRQPG	78,04	2,97	GWWEFLCGWGQLRQSN	100,24	5,87	GWWEKLFNDGRQNQPA	106,93	8,47	GWWEKLFNWGRQDQNK	57,54	14,89
63	MWWHRTYGIYYVNVRF	77,62	2,96	RWWEKLCVWGLLNMQM	99,00	5,80	GWWEKLFNRGRQNQPA	106,43	8,43	GWWEKLFNWGRSEMHM	57,38	14,85
64	VWRLLLKAFWQLRQPG	76,86	2,93	GWWEKLCVWQRLNMHN	98,76	5,79	GWWEKLTNWGRQNQPA	104,59	8,29	GWLEKLFNWGRQNQPA	57,28	14,82
65	GDRAYAKWCLWMFTTF	76,81	2,93	VWRELLKAFWCSCKLM	95,80	5,61	GWWEKLCAFWSNIHGC	104,58	8,28	GWWEKLFNWGRQNQVA	56,71	14,67
66	KKFFYLCVWWSVKKLG	76,15	2,90	GWWEKLCVWGRLNMHM	95,02	5,57	GWWEKLCSWGRLNMHG	103,69	8,21	GWWEKLFGWGRSNMHM	56,01	14,49
67	ERFARAKWCLWMFRDF	75,75	2,89	GWWEKLCVWWDFCEKK	94,23	5,52	GWWEKLCTWWWFNMHM	103,35	8,19	GYWEKLFNWGRQDQPK	55,82	14,44
68	TINKAWCVWTWTYGNA	75,41	2,87	GWWEKLCAFWWQIHGA	93,92	5,50	GWWEKLCSWGRLNMHM	103,21	8,18	GWWEKLFNWGRQFQPA	55,78	14,43
69	DFQRLLKMFWQLRNPG	73,90	2,82	GWWELLCGWGQLRQTG	93,18	5,46	GWWEKLCFWGRLNMHG	102,34	8,11	GWWEKLFNAGRQNHCA	55,26	14,30
70	DFQRWKGAFWQLRQSM	73,38	2,80	GWWEKLCTWAWQIMRK	93,18	5,46	GWWEKLCVWGRQNMHM	102,07	8,09	GWWEKLFNWQRQNMHC	55,04	14,24
71	KINKAWTLNFDFIGRK	73,07	2,78	GWFEKFCDWGRLNMHM	92,86	5,44	GWWEKLLTWWGQIHCA	100,93	8,00	GWWEKLFNWGRQVQPA	54,92	14,21
72	GWWEKLCVVWAVSKLG	72,54	2,76	GWWEKLCVWARLNMHG	90,18	5,28	GWWEKLCGWGRLNMHM	99,83	7,91	GWWEKLFNAGRQNHCA	54,78	14,17
73	GDRAYGKWCLWMFWDG	71,92	2,74	GWWEKLCVWGRLIGDC	90,14	5,28	GWWEKLSAWWWNIHGA	99,74	7,90	GWWEKLFNWGRSNMHM	54,34	14,06
74	VWFARLCVWWSVKKLG	71,64	2,73	GWFEKLCGWNRLNMHG	89,74	5,26	NWTEKLCEVWQVSKLG	99,38	7,87	SWFEKLFNWGRQNQPA	54,17	14,02
75	GAWEKLCAFWQVIGRC	70,53	2,69	GWWEKLCVWGMLIGSN	89,39	5,24	GWWEKLCGWGRLNMHM	99,22	7,86	GWWEKLFSWGMQNQPM	54,16	14,01
76	AKVEKLAVWWWNIHLG	70,20	2,67	GWWEKLCVWGRLNMHG	88,66	5,20	GWWEKLCNWGRQNQMA	98,03	7,77	SWWEKLACWGRQNMHG	53,93	13,95
77	MPRHRDTEWWWNIHGF	70,18	2,67	GWWEMLCDWGRLNMHM	88,38	5,18	GWWEKLFRWGRQNQPA	97,93	7,76	GWWEKLFGTGRQNMHF	53,71	13,90
78	GWWHRTYGILFSIYRF	69,59	2,65	GWWECLCVWGRLNMHM	88,21	5,17	GWWEKLCGFWNFCERK	97,65	7,74	GWWEKLFNWVRQNEPA	53,43	13,83
79	GWWYKGTAFWDFKGRK	69,25	2,64	GWWEKLCVWGRLNQPA	87,43	5,12	VWRELLKEVWQVSKLG	96,36	7,63	GWWEKLFNWGVNIHGA	53,30	13,79
80	VWRLLLKWCLWAFTEF	67,82	2,58	GWWEKLCVWGRLEMHM	87,35	5,12	GWWEKLCSWGRLNMHG	95,43	7,56	GWWEMLFNWGGNMHGA	53,30	13,79
81	AEVNWKGAFLCSIKLG	67,36	2,57	GWWEKLCVCKRQNMHM	86,73	5,08	GWWEKLCAWGRLNMHM	95,24	7,54	GWWEKLFNAGRQEYPA	53,14	13,75
82	AEVNWKGAFWNVKKLG	67,07	2,56	GWWRKLCTWWWQIHHM	86,40	5,06	GWWEKLCSWDRLNSHG	94,78	7,51	GWWEKLFNWVLQNQHM	52,54	13,59
83	DFERLKGAFYYFNVRF	66,72	2,54	GWWEKLCGWWQLRQPG	86,27	5,06	SWWEKLCGWGRQNMHM	94,25	7,47	GWWEKLFNLGRQNMHG	52,46	13,57
84	GMTHRACVWWSVKKLG	66,29	2,53	GWFEKLMAFWDFCERK	85,08	4,99	GMWEKLFNFWSNIHGC	93,63	7,42	GWWEKLANWGRNNQLA	51,45	13,31
85	AKVCYGKWCLWMFWDN	66,17	2,52	GWWEKLCVWGRLNMHQ	84,53	4,95	GWWERDSEVWQVSKLG	92,70	7,34	GWWEKLFNWGRQNQPA	51,19	13,25
86	LPVEKLGAFWGSIGRF	65,86	2,51	GWWEKLCVWGLLNMPA	84,50	4,95	GWWEKLCSWGRLNNCA	92,51	7,33	GLWEKLFNWGNFRMHM	51,13	13,23
87	TWFLKLCVWWSVKKLG	65,50	2,50	GWWEKLCVWGRLNQPA	84,09	4,93	GWWEKLCGWRRFNMHM	91,62	7,26	GWWEKLYNFWSNRQPA	50,26	13,00
88	KKFFYYTGFWGLRQEA	65,43	2,49	VWRELLKKWGRLNMHM	84,00	4,92	GWWEKLCAFWSNIHGC	89,89	7,12	GWWTKLFNWGRQNQLG	49,81	12,89
89	GWTHRATVWWSVKKLG	65,09	2,48	GWWEKLCVKGRLNDHG	83,43	4,89	GSWEKLFNWGRQNQVA	88,77	7,03	GWWEKLFNWGRQNQPA	49,59	12,83
90	SEVRWKGAFWQVSKTL	64,78	2,47	GWWEKLCVWGRLNMPA	82,87	4,86	GWWEKLGAFWQLRQMG	88,37	7,00	GWWEKLFNWGGNIHGA	49,44	12,79
91	LPKFYYTGFLCSIALF	63,98	2,44	GWWEKLCVWGRQRLPG	82,62	4,84	VWRELLKNWGRQNQPA	88,29	6,99	GWWEKLFNWGRQFQPL	49,31	12,76
92	VWRLLGRAFWQDRQTF	63,59	2,42	MPKHRDTEVWTVSKLG	82,47	4,83	GWWEKLCGWGALNMHM	87,65	6,94	GWWEKLFNFWSNRQPA	48,91	12,65
93	AFRLLGFAFWQDRQTG	63,56	2,42	GWWEKLCVWMWNIHGC	80,64	4,73	GWWEKLCSWGRQNQPA	86,87	6,88	LWWEKLFNWGRCNQLG	48,75	12,61
94	GWWHRACVWWSVKKLG	63,13	2,41	GWWEVLCAWGRLCMHG	80,53	4,72	CWWEKLSAWWDFIHGA	86,77	6,87	AWWEKLFNAGRDNCAM	48,62	12,58
95	GATHRACVWWSVSKLG	62,08	2,37	GWWEKLCVWGRLNQPA	80,46	4,71	GWWEKLCGFWNFCERF	85,51	6,77	GWWEKLFNWGRQNLPA	48,05	12,43
96	GWWYKGEGALKSIFRF	61,60	2,35	GWFEKLCDWGRLKMHG	79,93	4,68	GWWKLLKAWGRFNMHM	85,32	6,76	YWWEKLFNWGRQNGNG	47,43	12,27
97	VWFLKLCVWWSVKKLF	61,37	2,34	GWWEKLCAFWDFCEAG	79,29	4,65	GWWEKLCVWGRQNCHM	85,07	6,74	GWWEKLFNWGRQRQPA	47,00	12,16
98	DWFLKLAVWWSLNMHN	60,99	2,32	GSWEKLGAFWDFICRQ	78,66	4,61	GWWEKLCAWGRLNCHM	84,64	6,71	GWWEKLFNFWSNRQPA	46,61	12,06
99	MPRAYLKWCLWMFTDF	60,81	2,32	GWWEKLCVFWDFCERK	77,44	4,54	GWWEKLCGWGRQNQPA	83,17	6,59	GWWEKLFNFWNFNMHM	46,19	11,95
100	VKRAYAKWCLWMFTDF	60,43	2,30	GWWEKLCVFWDFCERK	76,92	4,51	GWWEKLCVWGRQNQPA	82,60	6,54	GWWEKLFDWGRQFQPA	45,82	11,85
101	SWWHRTYGIYYVNVLG	59,88	2,28	GWWEKLCAFWDFCECA	75,33	4,41	RWWEKLSAWWWFCERK	82,05	6,50	GWWEKLFNFYSNRMHM	45,35	11,73
102	DWFLKLCVWWNLRQPG	59,43	2,26	GWWEKLCGWGRLRQSN	75,02	4,40	DWWEKLCGFWNYCERK	81,87	6,49	GSWEKLFNWGMLNMHG	45,31	11,72
103	EPRRLKGAILFSIYRF	58,91	2,24	GWWEKLCVWGRLNMRM	71,98	4,22	GWFEKLCEWGRLNMHM	80,45	6,37	LWWEKLFNWGRQNMHG	44,61	11,54
104	SKVEKLGAFWGSIWDN	58,87	2,24	GWFEKLCDVWTVSKLG	71,69	4,20	CWRELLKAWWRLNMHG	80,39	6,37	GWWTKLFGWGRQNGHG	44,57	11,53
105	MPRHWKGAFGQLRWDN	58,00	2,21	VWRELLCVWGRLNMHT	71,65	4,20	GWWEKDQEVWTVSKLG	79,80	6,32	GWWEKVFNWGRQNQPM	44,07	11,40
106	ERFAKLGVWGRLNMHM	56,44	2,15	DFQVWACGWRRLNMHG	70,47	4,13	GWWEKLCAWGRLNMHG	79,65	6,31	GWWEKLFNWGRQNKPA	43,54	11,27
107	DFERLKGAFWDFIERK	56,10	2,14	GWQVWAYAFAQLRQPG	69,25	4,06	GWWEKLCTWGRSNMHG	79,50	6,30	LWWEKLFNWGRQEQVA	43,44	11,24
108	AKKFYYTGFLCDIKLG	55,94	2,13	GWWEKLCVWFQLRRPG	68,84	4,03	GWFEKLCAFWSNIHGM	79,28	6,28	EWWEKLFNLGRDNCAM	43,10	11,15
109	GGWYKALAFAYCNVLG	55,66	2,12	GWWELLCVWGRLNMHM	68,67	4,02	GWWEKLCVWGRQNGHA	78,98	6,26	GWWQKLFNWGRQNQLG	43,05	11,14
110	AEVNWSGAFWQLRQRA	55,02	2,10	AQMHKLCVWWWNIHGC	67,24	3,94	GWWEYLCAWWDFCARK	78,28	6,20	GWWEKLQNWGRQNQGA	43,02	11,13
111	GWWYNGSAFWQVIGRC	54,84	2,09	GWMEKLCAFGRLLMHF	66,40	3,89	GWWEKLCAWGRLNMGD	77,48	6,14	GWRELLKAWWRDNCAM	42,99	11,12
112	DWFLKLCVWLWMFWDN	54,78	2,09	GLWEKLCVWGRLNMHM	66,30	3,88	GWWEKLCVWGRMNMHM	77,28	6,12	VWWEKLFNAGRQNHCA	42,83	11,08
113	VWRLLLKELKNVKQPA	53,71	2,05	VWFELLKAWGCLNMHM	65,07	3,81	VWRELLFNWGRQNQPA	75,55	5,99	GYWEKLFNWGRQNQGA	42,63	11,03
114	GWWYKGSAFWYCNVEF	52,89	2,01	GWFEKLCDWGRSCSLG	64,75	3,79	GWWEKLEGWGRSNMHM	73,85	5,85	GWWEKLFNAGRQNGHG	42,53	11,00

115	AKVVKLCEVWWNIHLG	52,81	2,01	MPRHRDTEVWTVMKLG	63,83	3,74	GWWEKLCGWGRQNQAA	73,31	5,81	GWWEKLFKWGGNIHPM	41,72	10,80
116	AYVFRLEQILKSIFRF	52,32	1,99	GWWEKLCGWGRLYQRK	62,91	3,69	YWWEKLCGWGRFNMHA	72,71	5,76	GWWEKLFNWVRQKQPA	41,49	10,73
117	GWWYKGSAFWQLRQRA	52,30	1,99	DFQYWACGWGRLNMHG	58,67	3,44	GWLEKLCGWGRLNMHG	72,02	5,71	GWWEKLFNWGRQNQPK	41,17	10,65
118	CWWEKLTLNFDFIGRK	51,61	1,97	GWWLKLCTWGRLNMHG	57,97	3,40	SWWEKLGAFWQLRYHM	71,75	5,68	GYWEKLFNWGRQFQPA	41,13	10,64
119	LDRAYAKWCLDFIERK	51,09	1,95	VWWERDSEVWAVSKLG	57,22	3,35	GWRELSKAWWDFCARK	69,38	5,50	GWWELLKAWWRDNCAR	40,45	10,47
120	LVFHRLRGIVFFIYRG	50,54	1,93	GWNEKLCTWWWQIHGA	56,69	3,32	GWWEKLCVWGWNIHEA	69,33	5,49	GWWEKLFNWVRQNDPA	40,11	10,38
121	LPKFLGTAFWQDRQTD	50,30	1,92	GWWEKLCVWGRLVQSN	56,40	3,30	GWWEKLCCWGRLYMHG	66,97	5,31	GWWCKLFNWGRQNGHG	39,35	10,18
122	DWFLKLCVWAESIFRF	50,17	1,91	GYWEKLCAFWFFCERK	56,26	3,30	GWWEKLCGWGRFNMSM	66,36	5,26	GWWEKLFNWQRQNMHM	39,19	10,14
123	VDRAYAKWCLWMFCDE	49,43	1,88	GEANWKGAWWWNIHGA	56,07	3,29	GDWEKLFNFGRQNQPA	65,58	5,19	SWWEMLANWGRNKQPM	38,97	10,08
124	GWWCKQCRWGVLAMHV	48,05	1,83	GWVEKLCVWGRLNMHM	56,00	3,28	GWWERLCGGWRLNMHM	64,77	5,13	GWWEELFNWGRSNMHM	38,58	9,98
125	AKQYSGTALLDYIGRY	47,96	1,83	GWCEYLCSWGRLNMRK	55,97	3,28	KWWELLFLEGRQNQPA	63,91	5,06	SWWNKLFNLGRQNQPM	38,50	9,96
126	DFRLLKGACLWMFTDF	47,86	1,82	AEVNWKGAWWWSTKLG	55,60	3,26	CWWEKLFNGGRQSKLG	63,56	5,03	GWWEKLMNWGGNIHGA	38,42	9,94
127	DFRRLKGAILFSIGRK	47,83	1,82	GWWEKGCVWGRQNQPA	55,24	3,24	GWWEKLCVWGWQIHCA	63,51	5,03	GWWCKLFNWGRQNHCA	37,69	9,75
128	VWRLLLTGILAFILRF	47,56	1,81	LWWEGLCVWGLLNMHM	54,70	3,21	GWWEKLKAWNRFNMHM	63,33	5,02	GWWEGLFNFWSNRQPA	37,68	9,75
129	ARVSWKGAFSDFIERK	47,54	1,81	GWAEKLGDWGRLNMHM	54,05	3,17	GWWEKLCVGGRLNMHG	62,51	4,95	GWWEKLCGWSNFNMHM	37,59	9,73
130	LWWYEGCAVRQLRGDC	47,17	1,80	GWVEKLCVWGRLNMHN	53,31	3,12	GWWEKLCEWGRLNMYG	61,29	4,85	GWWEKLFNWQRQGMHM	37,00	9,57
131	LWWYEVCYFWWMFWDN	47,04	1,79	VWWEKLCVWGELNMHM	51,86	3,04	GWWEKLCVWGALNMHM	59,90	4,74	GVWEKLFNWVRENQPA	36,58	9,47
132	EKVEKLGAFWTSIGRA	46,57	1,77	GWFEKLCVWGVQNQPS	51,70	3,03	GWWEKLEGFWNFCERD	59,09	4,68	GQWEKLFNWTRNNQPA	35,44	9,17
133	AEVNWKCVWSSVKKLG	46,24	1,76	GWWMKLCVWGRQNQPA	50,99	2,99	GWWEKLCAFWSRKMHM	58,82	4,66	GWWQKLFNAGRQNHCA	35,36	9,15
134	GWWYKATAFWQLRQEA	46,00	1,75	GWWEKLCDRGRLNMHM	50,75	2,97	GCWEKLCGFWNFCERK	54,23	4,30	GWWEKLFAWGRQFQPK	35,19	9,11
135	VWRLLLCVWTWEYLNA	44,86	1,71	GWWEKCCLWGRLNQPA	50,61	2,97	GWWEKLCVFWSNIYGC	54,13	4,29	CWRELLKAWWRDNQPA	35,04	9,07
136	AKVLKLCVWWSVKVLG	44,60	1,70	DFQVWLCVWGRLNGHF	49,60	2,91	GWFEKLCAWNRLNMHG	52,03	4,12	GWFEKYFNWGRQNGHA	34,20	8,85
137	AFVMKLCVWGRLYMHM	44,26	1,69	DFQVWACQWGRLNMHG	49,34	2,89	GWWEKLCGDGRLNMHG	51,97	4,12	GWWEKLCNWGRCNQPA	33,04	8,55
138	AKVEKLCGFLCSIKLG	44,09	1,68	GWWFKLCFSGQLFESN	49,25	2,89	FWWEKLCVWGRQNQPM	51,47	4,08	GWWEKLFNRGRQFMPA	32,90	8,51
139	KKFFYYTGFWQLRQPA	43,65	1,66	GWWEYLCCWGRLIGDC	48,78	2,86	GWWEKLGAFWELRQPG	51,42	4,07	GWWMKLFGWGRQNMHF	32,71	8,46
140	FFVMKLCVFTWEYYYF	43,40	1,65	MPRHRDAEVWTVSQSN	48,74	2,86	SWWEKLGAFTALNMHM	51,35	4,07	GWWEKLFRWGRSNMHM	32,58	8,43
141	LDRAYATGFFGSIRDC	42,83	1,63	GWFEKLCVWGRLVMHG	48,38	2,84	GWWDYLCGWGRQSKLG	51,35	4,07	GWLEKLFAWVRQNGHG	32,46	8,40
142	LPTFRLEGWGRLNMHM	42,42	1,62	GWWEYLCVWWWNIHGC	44,82	2,63	FWWEKLCGWGRLTMHK	50,45	4,00	KWWLNLFNWGRQKQPV	30,64	7,93
143	TMNKKLCVWGRLNMHM	41,98	1,60	GTWEKLCVWGRLNMHM	44,80	2,63	GFWEKVFTWMRKNQPM	49,88	3,95	RRWEELFNWGRGNQLG	30,57	7,91
144	AEVNWKGAGGRLNMHM	41,32	1,57	AEVNWKGAWWRNIHHG	44,60	2,61	GWWEYLCGFWNDCERK	49,14	3,89	GWWFKLFTWGRQNGHG	30,04	7,77
145	EKCEKLGAFWGSIMRC	40,67	1,55	GWREKLCAFWRLNMHM	44,32	2,60	GWWEKLGAVWQLRQPG	47,56	3,77	GWWLKLFNWGRQFQPL	29,37	7,60
146	MPREKLSVWGRLNMHM	40,16	1,53	AEVNWRSAFWRLNMHD	43,97	2,58	GWWEVLCGWGAANMHM	47,26	3,74	CWWEKLGNWGRQNQGA	28,96	7,49
147	DFDRLLKELWNVMKLG	40,06	1,53	DFYVWAGVFWQLNQPA	43,78	2,57	GWKEKLFDWLTQADEK	47,12	3,73	GWWEKLMNLGRSNMHM	28,84	7,46
148	DFEEKLCVWGRLNMHM	40,00	1,52	GWWEKLCVAGRLIGDC	42,24	2,47	GWWEKLCAWGRTNMRM	46,20	3,66	CWRELLKAWGRQEQPA	28,62	7,40
149	MPRHRDTEVWAFIGRK	39,76	1,51	GQWEKLCGWGRLNMHG	41,56	2,44	GWWEELCVWGKQNCHM	45,21	3,58	GWWEKLFNKGRSNQLG	28,49	7,37
150	VWSYLGKELENAKKLG	38,73	1,48	GWWEKLDAFWDFCEDC	41,45	2,43	GWWEKLGAFWELRQPG	45,08	3,57	GWWRKLFNWGRQNGHM	27,82	7,20
151	LVFHRLRVWWAVKKLG	38,69	1,47	GWLEKLCDWGTVSKLG	40,63	2,38	GTWEKLCSWGRLNFHG	44,70	3,54	GWWEKLFYWGRQNGPM	27,23	7,04
152	GKTHRACVWWSEKKLG	37,78	1,44	GWREKLCVWGLLNMHG	40,50	2,37	GWYEKLCGWGRLNMHM	42,43	3,36	AWWEKYFRKGRQNGHG	26,87	6,95
153	GWWYKATAFAQLRQPA	37,71	1,44	QWNEKLCYVWTVSKLG	38,97	2,28	VWWEKLCEWGKFCARK	42,32	3,35	GWWEKLFVWGRQNQLG	26,68	6,90
154	VWRRCKGAFWQLRQKA	37,56	1,43	GWWEDLCGFWDFCERK	38,47	2,25	GWWEKLCVWGRQYMPG	40,92	3,24	GWGEKLFNFWSNNMHM	26,50	6,86
155	GWMEKLCVWGRVSKLG	37,01	1,41	GWGEKLCGWGRLNMHG	38,31	2,24	GWEEKLFNWGRFCNRK	38,70	3,07	GWWEKLFNRGGNIHGR	26,41	6,83
156	GWWYKATAFANCNVAC	36,94	1,41	VWRVSLCAFWDFCERK	37,96	2,22	CWRELLLAWWDFTMHM	38,49	3,05	GWWNKLFNWQRQNMHM	26,12	6,76
157	SDRRLKGAILFSEYRF	36,58	1,39	GEWEKLCVWGRLNMHM	37,40	2,19	VWRELLYAWGRFNQPA	38,39	3,04	GWWENLMNWGRQEQPA	25,62	6,63
158	DWFLYYTGFLGSIGDC	36,38	1,39	TWFEALCDWGRLNTYM	36,77	2,15	SWWLKLGAMWQLRQPG	37,74	2,99	CWREMLKNFWSNYQPA	25,45	6,58
159	LVFHYAKRCQWMFTDF	34,16	1,30	GWFEKTCDWGRLNVHM	36,45	2,14	DWWEKLCVWGTVSKLG	36,87	2,92	GWEEKLFNWLRQNQPA	25,02	6,47
160	GWWEYKCVSVRTVMHE	33,31	1,27	GWWELKKAWGRLNMHM	36,06	2,11	SWWETLCAFWSEIHGC	36,76	2,91	GWWEQLFNWGENMHPA	23,93	6,19
161	TWWYKGSAGWQVIGGA	32,98	1,26	GEWEKLCVWGRLNMHM	33,61	1,97	GWWEKLCVEGRQNMHM	35,43	2,81	GWWEKLASWGEQNCHM	23,91	6,19
162	MPRLLLKAFWQLRQPG	32,59	1,24	MPLHRAGAFWDFICRK	33,11	1,94	GWWHRLCGWGRFNMHK	34,94	2,77	GRWEVLFNWGRQNQPA	23,52	6,08
163	DEVSWNTGILAFIGRF	32,00	1,22	VWRETLCVWGRLNMHM	32,38	1,90	GWWEKLCGWGRSNKLG	34,77	2,75	GWWEKAFNWGVQEQPA	22,11	5,72
164	AWRLLEKCLWNVKKLS	31,84	1,21	GGWEKLCVWGRLNMPA	32,20	1,89	GWEEKLGAFWQLRQPG	34,28	2,72	GKWEYLFNWGGNIHGA	19,66	5,09
165	AYVNYNTGILAFIGLG	30,71	1,17	GWDEKLCVWGRLICRK	31,78	1,86	CWAEKLCGWGRLNMHG	33,60	2,66	SWGEKLFNWGRSNMHM	17,49	4,52
166	LDRAAAKAEWQLTYYF	30,17	1,15	DWVMKLCVWFRLVMCM	30,12	1,76	CFRELLKAWGRQNQPA	32,54	2,58	GWWAKLAVWGRNNQPA	17,21	4,45
167	GKTGRDCVWWTLRQPA	30,14	1,15	VWFEKLCVWGRLNMHM	29,58	1,73	RWWEKYRNRGKQDAPA	32,52	2,58	GWYFKLFNWGRQNQPA	16,76	4,34
168	GWWEKYTGFGGSIGDC	30,08	1,15	GCFEKLCRWGRLNMHG	28,86	1,69	CWRLKLCVWGRQNRHM	32,47	2,57	CWQEKLFGWGRQNMHF	16,65	4,31
169	LPTFRLEGILYCNVAF	29,27	1,11	GWFFKLADSGRLNMHM	28,80	1,69	SWWEKYCGWGALNMHM	32,36	2,56	SWWEVRFNCGRALQPK	15,64	4,05
170	AYRHRDTEVWAVSLRG	28,64	1,09	AEVNWKGAWGRLNMHA	28,38	1,66	GWWEKAFVWGRQNGHM	31,04	2,46	GWWELAYNWGRNNQPA	15,34	3,97
171	MWQYKGTALLDYIGHM	28,39	1,08	GWCEKLCVWGRLNDLG	28,10	1,65	GYWEKLCGWGALNMHM	27,23	2,16	GWWEKTFNWGRNNQPA	14,67	3,79
172	LPKFYYTRFLCSIKDN	27,76	1,06	GWEEKLCVWGRQNMHM	27,96	1,64	VWRELLKADGRFNQPA	25,60	2,03	GWWEKTFNWGRQEQVA	14,60	3,78
173	LDRAYAKAFWQLRQPV	27,56	1,05	GYWEYLCSWGGLNMHG	27,02	1,58	GWWEELCEWGRQNQPA	23,28	1,84	GWQEKLFNWGRQNQLG	13,38	3,46
174	SEVNWKGAFGDFIERK	26,65	1,02	GWWEKDGAFWCSVKLG	26,65	1,56	GWWHKLCGWGRQNMHM	22,87	1,81	AWWEKCFTWGRQNQPA	12,23	3,16
175	AKFEKACVFWQLRQEA	25,95	0,99	VQTLLLKFWNQLNMHM	22,57	1,32	MPWELLSAWAWNIHGA	21,75	1,72	EWWELLTNWGEQNVVG	10,92	2,82
176	VWRLLLTGFCGSIGDT	24,51	0,93	GWWEKRCVWGRLSKFG	21,90	1,28	CWRNKLFNWGRQNQPA	20,75	1,64	GWTEKAFNFWSNRQPA	10,51	2,72
177	ERFARLCVWESMFWDN	24,22	0,92	GWYGLLEARRRQIHGA	21,60	1,27	GWWEKRCGWGRQNMHM	20,72	1,64	KRWFKLFNWGRVNVHF	10,30	2,67
178	EKVELLCVWWSVIGLV	24,08	0,92	VWWDKLCDQGRLNMHM	21,28	1,25	MPRFRDQTWWWQIHCA	19,08	1,51	GVLEKLFNWQRQNMDM	10,11	2,62
179	MPRHKLCVWGRGNMHM	23,95	0,91	MCFEKLCDWGRVRMHM	19,04	1,12	GFWRVCFNYGRMNGPF	18,37	1,46	GWWYKLGNWSGQNQGG	9,37	2,42
180	TINKANGAFWDFIEKK	23,24	0,89	GWREKLGAAWDLNMHM	17,12	1,00	GWWEKLCETGRQNQPA	17,87	1,42	GWWEKRFNWGRQFQPA	9,09	2,35
181	GWAYKGTALLDYNMHM	23,22	0,88	AEVNWLCDWGRLNAHM	16,10	0,94	GWCESLCEWGRLNMHG	16,04	1,27	GYGEKLFNWVRQNQPA	8,96	2,32
182	KKFFYQTGFKGSIGDK	22,76	0,87	GWREKLCGWGRLNMMG	15,78	0,92	GWDEGLFFWGLQNQPR	15,15	1,20	CWRELLKAAARQNQPM	8,64	2,23
183	MPRHRDTEVYYVNYRF	21,12	0,80	VWWEKLVVWKCQNMDT	13,69	0,80	GWWEKSCEWGRQNQPA	14,96	1,19	GWWEKTASWGRQNCHM	7,76	2,01
184	GWQYKGTALLAVSKLG	20,46	0,78	AEVHRDSEVWNVSKGA	13,26	0,78	MPRFRDKAWWDVCQRK	13,68	1,08	CWREKLFNLGRQNQPM	7,45	1,93
185	LDMEYAGWTLWEQTRF	19,47	0,74	GWWEKADDWGRLNMHM	12,90	0,76	MPRFRDCAWGRLNMHM	12,61	1,00	GCWEKLASWKRQNCHM	6,92	1,79
186	MPMHRDTEVWGSIGRF	19,47	0,74	GWLEKLCDWESLNGSM	12,38	0,73	RWWVDQAKWGRQNQCA	10,76	0,85	GWWEKCSNFWRQDQPK	6,88	1,78
187	GWWYKATAFDYCNVAF	18,95	0,72	GRFEKLCDWGRLNMHM	12,22	0,72	GWNEKLCVWGRQNMHM	10,73	0,85	GQLVKLCGFWNFNMHG	6,47	1,67
188	DFRRLPGAFWQLRQPA	18,12	0,69	GWDEKLCVWGRLNQPA	11,62	0,68	GWWEKQCEWGRLNMHM	10,37	0,82	GWNEKLFNWGGNIHGA	6,10	1,58
189	AVVEKLGAFMGSIGRF	17,71	0,67	GWWEKRCVWGRQNQPG	10,65	0,62	GWWEKMCSWGCLNMHC	9,26	0,73	GFWEKQFNWGRQNQLM	5,34	1,38
190	DFQRWKGEGWNVKKLG	16,45	0,63	AEVNYKGAWGRLNMHG	9,66	0,57	MPREKLCGWGRQNMHS	7,73	0,61	GWWAKERAWGGGIHQR	5,26	1,36
191	LPRHRDTGILDSIGRF	16,27	0,62	GWWEYNCSMGRLNMRK	8,97	0,53	GWWEKDSAWGRFNMMM	6,70	0,53	GWKEKLFMEGQKFGHG	5,20	1,35
192	MKVEKACVNWCVKKLG	13,74	0,52	DFVNMRGAFWCSCKLG	8,80	0,52	GNWHRDSEVWYLRQPG	5,69	0,45	GWWEKCFNNVRYNQPA	4,08	1,06
193	MPYHRDTEVWAVFTDF	10,64	0,41	GWDLRVCKEGFLNMHG	6,67	0,39	GPRFRDQEEWTVSKLG	5,00	0,40	CKRNSLDCWWFDNTAF	3,81	0,99
194	MPRDRDTEFWDFIERK	8,91	0,34	GAVAKDVTLGRQNKPA	4,42	0,26	GWWRMNNGWNDQNQPA	4,03	0,32	SWWEKVANNGRNNQPA	3,80	0,98
195	EKVEKLKDFWQLRQPG	8,25	0,31	GDWEKLCAFGDFCERK	3,72	0,22	MPRFRDCVWGRQNMDM	3,33	0,26	GEFQKLCNWVRQNMFM	3,23	0,84
196	MPRHRDTVWDWEYGNA	6,57	0,25	MPRHRDTVWGRQNQTA	3,19	0,19	GWWERDQEVWTVSGLG	2,61	0,21	GWWEKDFNWVKQNQPM	3,14	0,81
197	MPGHRDTEVWAVQHGA	1,41	0,05	AEVEKLCVRGRLNMPA	2,07	0,12	CAWERCFDTGRQCQPA	1,45	0,11	GWWEVTTNRGRQLLPA	3,13	0,81
198	MPQHRATEVWASIGDC	1,29	0,05	AEVHRDSDWGRLNMHM	0,86	0,05	MPRFRDQEVGRLNMQG	0,69	0,05	GWWEKDASWGRQNCHM	2,81	0,73
199	TINKAWTEFSGSIGDC	0,98	0,04	AEVHRDSEVWANIGDC	-0,31	-0,02	MPRTRDQEVWTNCERK	0,22	0,02	GWTGRSFNWGDSNMTF	2,28	0,59
200	DFQRQDTEVWACSKLG	0,35	0,01	AEVNKLCVWGRQNNPA	-0,99	-0,06	GEWTCLFNEGLQNQPN	0,14	0,01	NWWEQSCNTVRQNGHC	1,02	0,26
	**Lead from Gen2**	**x**	**c**	**Lead from Gen3**	**x**	**c**	**Lead from Gen4**	**x**	**c**	**Lead from Gen5**	**x**	**c**
	GWWEKLCVWGRLNMHM	201,03	7,66	GWWEKLCAFWDFCERK	147,01	8,61	GWWEKLFNWGRQNQPA	222,25	17,61	GWWEKLFNWGRQDQPK	108,41	28,05
	AKVEKLCVWWWNIHGA	106,92	4,07	GWWEKLCVWGRQNQPA	125,90	7,38	GWWEKLSAWWWNIHGA	134,67	10,67	GWWEKLFNWGRQFQPA	93,44	24,18
	AEVNWKGAFWDFIERK	92,87	3,54	GWFEKLCDWGRLNMHM	124,85	7,32	GWWEKLCEWGRLNMHG	130,64	10,35	GWWEKLFNWGMLNMHG	88,88	23,00
	MPRHRDTEVWAVSKLG	88,43	3,37	GWWEKLCGWGRLNMHG	122,62	7,19	GWWHRDSEVWQVSKLG	118,04	9,35	GWWEKLFNWGRQEQPA	85,82	22,21
	LDRAYAKWCLWMFTDF	83,17	3,17	GWWEKLCVWGRLNMHM	122,11	7,16	GWWEKLCGFWNFCERK	117,44	9,30	GWWEKLFNWGRQNGHG	85,53	22,13
	DFQRWKGAFWQLRQPA	81,28	3,10	MPRHRDTEVWTVSKLG	117,54	6,89	MPRFRDQEVWTVSKLG	113,40	8,98	GWWEKLFNWGGNIHGA	84,07	21,75
	SEVRWKGAFWQLRWDN	76,98	2,93	GWWEKLCTWWWQIHGA	115,37	6,76	GWFEVSCNWCKLNMMM	111,33	8,82	GWWEKLFNWGRSNMHM	82,02	21,22
	EKVEKLGAFWGSIGRF	76,69	2,92	GWWEKLCVWGRLNMHM	105,29	6,17	CWRELLKAWWDFCARK	108,08	8,56	GWWEKLFNWGRQNQPA	79,46	20,56
	LPVNWKGAFWQLRQEA	76,42	2,91	GWWEKLCVWGLLNMHM	99,76	5,85	VWRELLKAWGRFNMHM	105,40	8,35	GWWEKLFNWGRQNQLG	73,21	18,94

	VWRLLLKAFWQLRQPG	71,63	2,73	DFQVWAGAFWQLRQPG	98,27	5,76	GWWEKLCSWGRLNMHG	102,85	8,15	GWWEKLFNFWSNRQPA	68,68	17,77
	KKFFYYTGFLGSIGDC	71,41	2,72	VWRELLKAWGRLNMHM	96,53	5,66	CWWEKLCGWGRLNMHM	102,72	8,14	GWWEKLFNWGRQNMHG	62,49	16,17
	GDRAYGKWCLWMFWDN	71,15	2,71	GWWEKLCVWWWNIHGC	96,48	5,65	GWWEKLCGWGRQNMHM	102,29	8,10	GWWEKLFGWGRQNMHF	56,30	14,57
	LPKFYYTGFLCSIKLG	69,34	2,64	VWWEKLCVWGRQNMHM	93,82	5,50	GWWEKLCGWGRLNMHM	97,77	7,75	GWWEKLFNLGRQNQPM	54,33	14,06
	AKVEKACVWWSVKKLG	67,71	2,58	GWWEKLCVWGQLRQSN	92,17	5,40	GWWEKLCGWGRFNMHM	97,58	7,73	SWWEKLANWGRNNQPA	51,03	13,20
	DWFLKLCVWWSVKKLG	67,19	2,56	GWWEYLCSWGRLNMRK	91,29	5,35	GWWEKLCAFWSNIHGC	96,81	7,67	GWWEKLFNAGRQNHCA	49,49	12,81
	TINKAWTLNFDFIGRK	67,05	2,55	GWWEKLCVWGRLNQPA	90,83	5,32	GWWEKLCGWGRLNMHG	93,45	7,40	GWWEKLFNWGRQNQGA	47,98	12,41
	DFERLKGAFWWMFWDN	66,71	2,54	GWWEKLGAFWDFICRK	88,85	5,21	SWWEKLGAFWQLRQPG	91,28	7,23	CWRELLKAWWRDNCAM	47,30	12,24
	VWRLLLKELWNVKKLG	63,07	2,40	GWWEKLCVWGRLNMHM	85,58	5,01	GWWEKLCAWGRLNMHM	90,75	7,19	GWWEKLFNWVRQNQPA	45,85	11,86
	DFRRLKGAILFSIYRF	61,44	2,34	AEVHRDSEVWAVSKLG	83,73	4,91	GWWEKLCVWGRQNCHM	87,13	6,90	GWWEKLASWGRQNCHM	40,84	10,57
	GWWHRTYGIYYVNVRF	60,97	2,32	GWWEKLCVWGRLNMHG	78,55	4,60	GWWEKLCGWGALNMHM	86,99	6,89	GWWEKLFNWLRQNQPA	39,48	10,22
	AFVMKLCVWTWEYGNA	52,60	2,00	GWWEKLCVWGRLIGDC	66,35	3,89	GWWEKLCTWWWQIHCA	83,21	6,59	GYWEKLFNWGRQNQPA	37,01	9,58
	GWWYKGSAFWQVIGRC	52,22	1,99	AEVNWRGAFWCSCKLG	63,23	3,71	GWWEKLCVWGRQNMHM	81,97	6,49	GWWEKLFNWQRQNMHM	34,37	8,89
				GWWEKLCVWGRLNMPA	59,88	3,51	GWWEKLCVWGRQNMHM	72,21	5,72	GWLEKLCGFWNFNMHM	33,30	8,62

	Generation 7	x	c	Generation 8	x	c	Generation9	x	c	Generation10	x
1	**TWWEKLFNWGRQEQPA**	121,83	34,42	**SWWEKLFNWGMLNMPK**	72,31	46,38	**SWWEKLFNFGMQNCPA**	72,20	62,77	**SWWEKLFNWGCQNQHM**	81,11
2	**QWWEKLFNWGRQNQPM**	108,20	30,57	**KWWEKLFNWGMLNMHG**	68,43	43,89	**NWWEKLFNWGMNDQHM**	68,73	59,75	**SWWEKLFNFGMMDQPK**	79,08
3	**GWWEKLFNWGMQNGHG**	107,42	30,35	**GWWEKLFNFGCQNMTL**	66,38	42,58	**GWWEKLFNWGMQDQPK**	66,58	57,88	**SWWEKLFNWGMYDQLK**	75,49
4	**GWWEKLFNFGCQNQHM**	102,30	28,90	**GWWDKLFNFGCQNQHM**	64,40	41,31	**KWWEKLFNDGFLNMHG**	66,42	57,75	**NWWEKLFNWGMQDQPK**	73,76
5	**GWWEKLFNWGMLNMGG**	100,32	28,34	**NWWEKLFNWGMEDQLK**	59,83	38,38	**NWWEKLFNWGMENMTL**	65,09	56,59	**KWWEKFFNWGMQNCPA**	73,33
6	**GWWEKFFNWGMEDQPK**	99,27	28,04	**SWWEKLFNWGRDNGMG**	57,43	36,84	**GWWDKLFNWGMEDQLK**	64,21	55,82	**SWWEKLFNFGMQDQPK**	71,05
7	**GWWEKLFNWGCVNMHA**	98,45	27,81	**SWWEKLFNWGRQNQPM**	56,39	36,17	**KWWEKFFNWGMMDQPK**	59,69	51,89	**SWWEKLFNFGMQNCPA**	68,79
8	**EWWEKLFNWGRDNGMG**	96,55	27,28	**RWWEKLFNWGMQDQPK**	55,55	35,63	**GWWDKLFNWGCQNQHM**	57,08	49,63	**SWWEKLFNWGMQDQPQ**	66,55
9	**SWWEKLFNWVGQNQPA**	96,48	27,26	**SWWEKLFNFGMQNGHN**	55,44	35,56	**TWWEKLFNWGCQNQHM**	56,94	49,50	**TWWEKLFNWGMQDQPK**	66,22
10	**GWWEKLFNWGRQFQDA**	96,35	27,22	**TWWEKFFNWGMMDQPK**	54,69	35,08	**CWWEKLFNWGMLDQSK**	56,65	49,25	**KWWEKFFNWGMMDQNK**	65,61
11	**SWWEKLFNWGMLNMHG**	95,18	26,89	**CWWEKLFNWGCSCQPK**	54,66	35,06	**GWWEKLFNFGMQNGHN**	56,27	48,92	**TWWEKLFNWGCQNMHG**	63,73
12	**GWWEKLFNWGMLNMGG**	94,50	26,70	**SWWEKLFNQGMLNMHQ**	53,47	34,30	**GWWEKLFQWGMQDQPK**	56,19	48,85	**TWWEKLFNVGMQNGHN**	63,22
13	**EWWEKLFNWGMLNMHG**	94,33	26,65	**GWWEKLFGFGCQNQHM**	53,42	34,27	**TWWEKLTNWGCQNTHM**	55,12	47,92	**SWWEKLFNWGCQNQLK**	62,57
14	**SWWEKLFNWGRQDQPK**	93,31	26,36	**GWWEKLFNWGMQDQPK**	53,39	34,25	**SWWEKFFNWGCQNQHM**	55,04	47,85	**GWWDKLFNWGMLDQSK**	59,83
15	**GWWEKLFNWGCSNQPK**	92,78	26,21	**GWWEKLFNWGMLNMGG**	53,27	34,17	**SWWEKLFNFGMQNGHN**	54,62	47,48	**TWWEKLFNWQMQDQPK**	59,75
16	**GWWEKLFNWGRQQQTA**	92,32	26,08	**CWWEKLFNWGMLNKHD**	52,92	33,95	**GWWEKFFNWGMMDSPK**	54,34	47,24	**GWWDKLFNWGCQNQHM**	59,66
17	**CWWEKLFCWGRQNQPA**	92,11	26,02	**GWWEKLLNWGRQNQPA**	52,49	33,67	**SWWEKLFNFGMQNTHM**	54,20	47,12	**SWWEKLFNWGMYDKPK**	59,52
18	**GWWEKLFNWGRQNGCG**	91,74	25,92	**SWWEKLFNVVGQNQPA**	50,72	32,54	**GWWEKLFGYGCQNGQG**	54,17	47,10	**NWWEKLFNWGCQNQHM**	59,50
19	**GWWEKLFNLGCQNQPQ**	91,45	25,84	**TWWEKLFNWGRDNGMG**	50,55	32,43	**TWWEKLFNWGMQDQPK**	53,33	46,37	**CWWEKLFNWGMQNQPK**	58,00
20	**GWWEKLFNWGRQQQTA**	91,37	25,81	**TWWEKLFNSGRQDQPK**	50,41	32,34	**SWWEKLFNWGCQNLTL**	53,22	46,27	**GWWEKLFNWGMQDQPK**	57,64
21	**CWWEKLFNWGMQNGHG**	91,05	25,72	**DWWEKLFNWGRQDQPK**	50,39	32,32	**GWWDKLFNFGCQNQHM**	52,70	45,81	**KWWEKFFNWGCQNMHM**	56,84
22	**SWWEKLFNWGRQNQPA**	89,17	25,19	**GWWEKLLNWGRQNQPA**	49,93	32,03	**SWWEKLLNWFRQNQPA**	52,55	45,68	**SWWEKLFNCGQQNTTL**	56,25
23	**GWWEKLFNWGYQDQPK**	88,43	24,98	**SWWEKLVNWGYQDQPK**	49,59	31,81	**GWWEKLFNWVMQDQPK**	52,45	45,60	**NWWEKLFNWGMNDQPK**	56,15
24	**QWWEKLLNWGRQNQPA**	87,94	24,84	**GWWEKFFNWGCQNQHM**	49,45	31,72	**CWWEKLFNWGGQNQVA**	52,12	45,31	**NWWEKLFNWGMNNCPA**	55,92
25	**GWWEKLFNWGRDNGHM**	87,61	24,75	**GWWEKLFNWGRQEQQA**	49,44	31,71	**SWWEKLFNWGMQDQPK**	51,24	44,55	**TWWEKLFNWGMENTTL**	55,73
26	GWWEKLFNWGRQEQPA	87,45	24,71	YWWEKLLNWGRQQQDA	49,28	31,61	QWWEKLFNWGRQNQPM	51,22	44,53	SWWEKFFNWSCQNQHM	55,57
27	GWWEKLFNWGMQNGHN	86,85	24,53	GWWEKLFNVGRQNQPM	49,27	31,60	SWWEKLLNFGMQQGHN	50,90	44,25	GWWEKLFNWGMQDQPK	55,51
28	GWWEKLFNWGRDNGHM	86,31	24,38	GWWEKLFNWGMLNMPK	48,60	31,17	AWWEKLFNWGRDNGMG	50,21	43,65	SWWEKLFNFGMQNGHD	54,99
29	EWWEKLFNWGMLNMHG	86,11	24,33	QWWEKLFNWGNQFQDA	48,54	31,13	SWWEKLFNWGMQDQPK	50,15	43,60	TWWDKLFNWGCQNGHM	54,75
30	GWWEKLVNWTCQNQPM	84,97	24,00	QWWEKLFNLGCCRQPQ	48,42	31,06	GWWEKLFNWGCQNQHM	49,96	43,44	NWWEKLFNWGMNDQHM	54,69
31	CWWEKLFNWGAQNGHG	83,71	23,65	GWWEKLLNWGGQNQPA	48,20	30,92	SWWEKLFNFGCQNMTL	49,38	42,93	SWWEKLFNWGCCNKHM	54,05
32	GWWEKLFNWGRQEQPA	83,06	23,46	SWWEKLFNWGRDTGMG	48,10	30,85	NWWEKLFNWGMEDQLK	49,32	42,88	NWWEKLFNWGMEDQLK	53,82
33	GWWEKLFNLGMLNMHG	82,23	23,23	GWWEKLFNMGMQNGHG	48,06	30,83	GWWEKLFNWGMDNGMG	48,93	42,54	TWWEKLFNSFRQNQPA	53,67
34	GWWEKLFNWGRQDQPK	81,79	23,11	QWWEKLFNWGRQNQPM	47,17	30,26	SWWEKLFNWYMEDQLK	48,33	42,02	TWWEKLFNWGFCDQHM	52,92
35	GWWEKLFNWGRSNMHM	81,70	23,08	GWWEKLFNFGCQNAHM	46,96	30,12	NWWEKLFNFYCQNMTL	48,14	41,85	SWWEKLFNFGMMDSPK	52,82
36	QWWEKLFNWGRQNQPA	81,69	23,08	GWWEKLFNWGMQNGHN	46,76	29,99	SWWEKLSNWGRQEQQA	48,13	41,84	KWWEKFFNWGMLNMHG	52,71
37	GWWEKLFNWGCSNMHA	81,46	23,01	SWWEKLFNWGRQNGHY	46,61	29,90	SWWAKLFNWGDQDQPK	47,84	41,59	KWWEKLFNDGFLNMHG	51,48
38	GWWEKLFNWGREDGPK	80,98	22,88	SWWEKLFNWVGQNYPA	46,51	29,84	TWWEKLFNSGRQKQDA	47,84	41,59	GWWEKFFNWGMQDQPK	51,09
39	GWWEKLFNWGRQNQPA	80,81	22,83	GWWEKLFNWKNQQQTA	46,49	29,82	RWWEKLFNWLMQDQPK	47,79	41,55	SWWAKFFNWGCQNQHM	51,08
40	GWWEKLFNWGRQQQTA	79,88	22,57	GWWEKLFNWDCQNQHM	46,21	29,64	YWWEKLFNWTMQDQPK	47,57	41,35	NWWEKLFNFGMQNTHD	51,02
41	GWWEKLFNWGRQNGHA	79,83	22,55	CWWEKLFNLGCQNQPQ	45,88	29,43	SWWEKFFNWGMMDQPK	47,24	41,07	SWWEKLFNFGMQNTHV	50,86
42	GWWEKLFNWGMLLMHG	79,67	22,51	GWWEKLFNVGCSNGHG	45,86	29,42	TWWEKLFNVGGQNQPA	46,99	40,85	KWWEKLFNFGMQNGTN	50,39
43	GWWEKLFNWGRQNGCG	79,40	22,43	EWWEKLFNWGCVNMHA	45,68	29,30	GWWDKLFNFGCQMVHM	46,77	40,66	SWWDKLFNFGCQNQHM	50,24
44	GWWEKLFCWGRQNQPA	79,35	22,42	GWWEKLFNWGMRNGHG	45,65	29,28	TWWEKLFNWGMQDQPK	46,14	40,11	GWWEKLFNWGCQNDTL	50,22
45	GWWEKLFNWGRTNQPA	79,02	22,32	GWWEKLFNWGRQQVCG	45,64	29,28	SWWEKLFNFGCSCYPK	45,63	39,67	GWWEKLFNWVMLDSPK	50,18
46	GWWEKLFNWGRDNGPA	78,81	22,26	SWWEKLFCWGRQNEPA	45,58	29,24	GWWEKLFNFSCQNQHM	45,50	39,56	CWWEKLFNWLMLDQSK	49,58
47	QWWEMLFNWGRQNMGG	78,67	22,22	SWWEKLFNWGMLNMGG	45,53	29,20	GWWEKLFNWGMGNMPK	45,39	39,46	TWWEKLFNFGCQNQHM	49,55
48	GWWEKLFNWGRQEQHG	78,63	22,21	GWWSKLFNWGMQNGPQ	45,48	29,17	SWWEKLFNWGRQDQPT	45,18	39,28	SWWEKLFNWGCQNQTL	49,38
49	GWWEKLFNWGCSNMHA	78,09	22,06	EWWEKLFNWGRDDAPK	45,48	29,17	TWWEKFFNWGMMDQPV	45,09	39,20	TWWEKLFNDGSLNMHG	49,12
50	CWWEKLFMWGMLNMGG	77,46	21,88	LWWEKLFNWVGKNQPA	45,21	29,00	GWWEKLFNFGCQNMTL	43,95	38,21	GWWEKLFGYGCQNGQG	49,01
51	GWWEKLFNWGRQEQPA	77,32	21,84	GWWEKLFAFGCQNQHM	44,75	28,70	GWWEKLFNTGCQNMPK	43,67	37,97	GWWEKLFNFGMQNGHN	48,96
52	GWWEKLFNWGMQNQPA	77,23	21,82	GWWEKLFCWGRQNQPA	44,14	28,32	CWWEKLFNWGMLNMHG	42,96	37,35	KWWEKFFNWGMQNQHM	48,88
53	KWWEKFFNWGRQEQPA	77,09	21,78	GWWEKLFNWKRQDQPQ	43,12	27,66	SWWEKLFNVVNQNQPA	42,95	37,34	CWWQKLFNWGMENMTL	48,68
54	GWWEKLFNWGRQNQPA	76,93	21,73	SWWEKLFNWVGQNQPK	43,08	27,64	GWWEKLFNWNCQNQHM	42,72	37,14	GWWEKLFNTGMQDQHM	48,67
55	GWWEKLFNWGMLNNHG	76,85	21,71	TWWERLFNWGCLNMHG	42,99	27,58	SWWEKLFNWGMLNMHG	42,62	37,05	NWWEKLFNWLMQDQPK	48,60
56	GWWEKLFNWGRQNGCG	76,77	21,69	CWWEKLFNWGMLNMGG	42,89	27,51	NWWEKLVNWGYQDQPK	42,29	36,77	SWWEKLFNWVMQDQPK	48,51
57	GWWEKLFNWGRQNGHG	76,63	21,65	SWWEMLFNWGRQDQPK	42,85	27,48	GWWEKLFNWGDMDQPK	42,24	36,72	KWWEKLFNSGFLNMPA	47,27
58	GWWEKLFNWGRQNGHG	76,03	21,48	GWWEKLFNWGMYNMHG	42,70	27,39	TWWEKLFNWFMQDQPK	42,22	36,70	SWWEKLFNWGCQNQHM	47,23
59	GWWEKLFNWGRQNQPA	75,97	21,46	EWWEKLFNWGMLNMHG	42,64	27,35	RWWDKLFNFGCQNQHM	42,10	36,60	GWWDKLFNWGMLDDSK	47,04
60	GWWEKLFNWGRQNQPM	75,66	21,37	SWWEKLFNWGMQNKYN	42,62	27,34	SWWEKLFNFGMTNQPK	41,52	36,09	KWWEKFFNWGMEDQLK	46,60
61	GWWEKLFNWGCEDQPK	75,35	21,29	GWWEKLFNFGRQFQDA	42,39	27,19	SWWEKLFNWGMLNMPK	41,21	35,83	GWWDKLFNFGCQNQAM	46,23
62	GWWEKLFNWGMLNMHG	75,03	21,20	GWWEKLFCWGMQNQPA	42,36	27,17	RWWEKLFNWGRQNQPM	40,64	35,33	SWWEKLFNDGFLNMHG	45,91
63	GWWEKLFNWGGQNQPM	74,83	21,14	TWWEKLFNWGRQLQPK	42,31	27,14	SWWEKLFNWGRQNQPM	40,44	35,16	NWWEKLANWGMNDQHM	45,37
64	GWFEKLFNWGNLNMHA	74,73	21,11	QWWEKLLNWGRQNGCG	42,21	27,07	SWWEKLFNWGRQNQPM	40,44	35,15	SWWEKLFNWGMMDSPK	45,30
65	SWWEKLFNWGRQDQKK	74,69	21,10	GWWEKLFNVGMLNMAG	41,88	26,86	SWWEKLFNWNCQKQHM	40,43	35,15	KWWEKLFQWGMNDQPK	44,96
66	GWWEKLFNLGCQNQPM	74,67	21,10	CWWEKLFNWGRDNGMG	41,78	26,80	GWWEKLFNFGCQNMTK	39,71	34,52	GWWDKLFNWSMNDQHM	44,95
67	GWWEKLFNTGMLNMGG	74,49	21,04	GWWEKLFNWGMGNGHG	41,61	26,69	GWWEKLFNQGMLNMHQ	39,58	34,41	SWWEKLFNWGMENMTL	44,69
68	GWWEKLFNWGRQQQPM	74,45	21,03	TWWEKLFNWGRQEQPA	41,55	26,65	QWWEKLFNWGVLNKHD	39,36	34,22	TWYEKLFNWGMQDQPK	44,59
69	SWWEKLFNWGRQNQPA	74,11	20,94	EWWEKLFNWGRQEQPA	41,48	26,61	YWWEKLFNWGRDNGMG	39,30	34,17	GWWDKLFNWGFQNQHA	44,15
70	GWWEKLFGWGRDNMHQ	73,27	20,70	CWWEKLFCVGRQNQPA	41,23	26,45	CWWEKLFNSGRQDQPK	39,01	33,91	SWWEKLFNDGFLNRHG	44,15
71	GWWEKFFNLGCSAMHA	73,16	20,67	GWWDKLFNWGRQDQPK	41,19	26,42	GWWEKLFNWGQLNKHD	39,00	33,90	GWWDKLFNWGCQFTHM	44,11
72	GWWTKLFNWGMQNGHG	72,11	20,37	GWWEKLFNWGMQNQTA	41,18	26,41	CLWEKLFNWGCSDQPK	38,38	33,37	SWWEKLFNFGMQNCPA	43,93
73	CWWEKLFNWGRQNCHG	72,03	20,35	GWWEKLFNWGMQNGDG	41,17	26,41	GWWEKLFNWGRQQQDA	37,94	32,98	SWWNTLLNWFRQNQHM	43,88
74	GWWEKLFNAGRQEQPC	71,88	20,31	GWWEKFFCWGRQNQPM	41,14	26,39	DWWEKLFNGGRQNQPM	37,92	32,96	SWWESLFNFGCQNQHM	43,23
75	GWWEKLFNWGRSNMHM	71,30	20,14	DWWEKLFNWGRQFQPA	41,12	26,38	GWWEKLFNFLCQNQHM	37,79	32,85	SWWEKLFNFGMQNQHM	43,17
76	GWWEKLFNWGRQCQPA	71,29	20,14	GWWEKLFNWGGDNGHM	40,87	26,22	SWWEKLFNQGRLNMHD	37,44	32,55	GWWDKLFNFGCQNQHM	42,63

77	GWWEKLFNWGNNNMHY	71,17	20,11	SWWEKLFNWGCVLMHA	40,38	25,90	SWWEKLFNVVGNDQPK	37,40	32,51	GWWEKLFNWGMNDQHM	42,39
78	GWWEKLFNWGRENGCE	70,82	20,01	TWWSKLFNWVGQNQPK	40,29	25,85	SWWTKLFNWGCSCQPK	37,37	32,49	NWWEKLFNWGRTNQPA	42,07
79	YWWEKLFNWGRQEQPA	70,72	19,98	GWWEKLFNWGMLNMGG	39,93	25,61	NWWEKLFNWQRDNGMG	37,23	32,37	CWWEKLTNWGCQNTHC	41,39
80	GWWEKLFNWGRQDQRK	70,63	19,95	GWWEKLFNWGKEDQPK	39,93	25,61	GWWEKLLNWGRQNGMG	37,22	32,35	NWWEKLFNNGCQNTHM	41,36
81	GWWEKLFNWGRQNGHG	70,57	19,94	CWWEKLFNWGMVNMHA	39,90	25,59	GWWEKLFNWGMLNMAK	37,20	32,34	NWWEKLLNFGMQMTHM	41,32
82	SWWEKLFNWGRQQQTA	70,34	19,87	GWWEKLFNWVMQNGHN	39,81	25,53	YWWEKLLNWGRQNQHM	37,15	32,30	MWWEKFFNWVMQDQPK	40,73
83	GWWEKLFNLYCQNQPM	69,92	19,75	TWWTKLFNLGCQNQPE	39,78	25,52	SWWEKLANWCMLNMGG	36,98	32,15	GWWDKLFNFGCQNQPK	39,99
84	GWWEKLFNWGRQDQPK	69,91	19,75	GWWEKLFNLGCQNQPQ	39,63	25,42	GWWEKLFNSGRQDQPK	36,93	32,11	SWWEKLTNWGMQDQPK	39,85
85	GWWEKLFGWGRQNMHF	69,88	19,74	EWWEKLFNWGMQNGNG	39,56	25,38	SWWSKLFNDGCQNMTL	36,62	31,84	GWWEKLFQFGMQNTHM	39,44
86	GWWEKLFQWVGQNQPA	69,15	19,53	TVWEKLFNWGCSNQPK	39,55	25,37	TWWEKLFYSGRQNQHM	36,44	31,68	SWWEKLFQFGMENMTD	39,40
87	NWWEKLFNWGRQEQPA	69,00	19,49	GWWEKLFNWGRQQQTT	39,44	25,30	SWWEKLFLQGRDNGMG	36,09	31,38	KWWEKLLNSFRQNQPA	39,28
88	GWWEKLFNWGRQVQPA	68,92	19,47	SWWKKLFNWGDLNMHG	39,41	25,28	SFWEKLFNWGMLNMPK	35,94	31,25	GWWEKLFNDGFRNMHG	38,93
89	GWWEKLFNWGMLNDGG	68,58	19,37	TWWSKLFNWGSQEQPA	39,35	25,24	KWWEKLFNWGRLNMGV	35,87	31,18	GWWEKLFNDGFLNMHG	38,92
90	GWWEKLFNWGLRNGHG	68,02	19,22	GWWEKLFSWGMEDQPK	39,15	25,11	KWWEKLFNWGMLNMHG	35,77	31,09	GWWEKLFNWGMNDMHM	38,90
91	SWWEKLFNYGRQNQPA	67,36	19,03	QWWEKLFNWGRQEQPA	38,90	24,95	KWWETLMNWGMLNMHQ	35,54	30,90	GWWEKLFNWNMQDQPK	38,87
92	GWWEKLFNLTRDNGHG	67,26	19,00	GWWEKLLGWGRQNQPA	38,77	24,87	TWWEKLRNWGMLNMGG	35,53	30,89	KWWEKLFCDGFLNMHG	38,64
93	GWWEKLFNSGRQQQTA	67,08	18,95	GWWEKLFNWGMQNGHG	38,30	24,57	LWWEKLFNSGRQDQPK	35,45	30,82	LWWEKLFNWGGQNQVA	38,14
94	GWWEKLFNWGRQCQPA	66,90	18,90	GWWEKLFNFGCQNQHM	37,76	24,22	CWWEKLFNWGELNMHG	35,30	30,69	SWWEKLFNFGMQNGHN	37,54
95	GWWEKLFNWGRSNMHM	66,43	18,77	CWWEKLFCWGRQFQPM	37,64	24,15	CWWEKLMNWGMQDQPK	35,20	30,60	GWWEKLFNWVMQDQPK	36,96
96	GWWEKLFGWGRQNMHF	65,92	18,62	CWWECLFNWGMQNGHK	37,25	23,90	GWWEKLFGFGRQEQQA	35,07	30,49	GWWEKLFGYGCQNGLG	36,57
97	QWWSKLFNLGCQNQPM	65,61	18,54	GWWEKLFRWVGQNQPA	36,82	23,62	NWWEKLFDFECQNQHM	34,87	30,31	GWWEKLFRWGMNDQHM	36,26
98	GWWEKFFNWGNQNGHG	65,55	18,52	FWWEKLFNWGRQNGCG	36,55	23,45	YWWEKLFNQGMLNMHK	34,58	30,06	GWWEKLFGYGCQNGQG	35,71
99	SWWEKLFGWGMLNNHG	65,12	18,40	TWWEKLFRWGMQNGHG	36,36	23,32	SWWEKLFNWGMLNMPK	34,47	29,97	GWWTKLFNWGMEDTHM	35,38
100	GWWDKLFNWGRQEQPA	65,08	18,38	GSWEKLFNWGRDNGMG	36,31	23,29	GWWEKLFGFGCQNQPA	34,43	29,93	GWWGKLFNWVMQDQPL	35,32
101	GWWEKLFNWGRQFQPA	65,08	18,38	GWWENLFNWGRQNQPA	36,31	23,29	GWWEKLFNWDMEEQLK	34,41	29,92	GWWEKLFGYGCQDQHM	35,02
102	GWWEKLFNWGRQNQPA	63,92	18,06	GWWEKLKNWGRQEQPA	36,30	23,28	DWWEKLFNWGRGDQPG	34,08	29,62	GWWEKLFQWDCQNQHM	34,77
103	GWWEKLFNWGMLNYHG	63,68	17,99	SWWEKLFNWVSQNQPM	36,22	23,23	GWWEKVFNWQCQNQHM	34,06	29,61	GWWEKLFNWGMQDQCG	34,58
104	GWWEKLFNWVGQNQPA	63,41	17,91	EWWEKLSNWGRQEQPA	36,00	23,09	SWWEKLFNWGMLNFPK	33,82	29,40	NWWEKLFMWGGQNQVA	34,52
105	GWWEKLFNWGRQDQPK	63,01	17,80	QWWEKLFNWGRQNQPA	35,09	22,51	KWWEKLFNWGCQRQHM	33,03	28,72	GWWDKLFNWGCQGQHA	34,44
106	QWWQKLFNWGRSDQPK	62,72	17,72	GWWEKLFNWGRDNGHM	35,02	22,46	KWWEKFENWGRQEQQA	32,95	28,65	GWWEKLFQCGMQDQPK	34,21
107	GWWEKLFNWGRKQQTA	62,44	17,64	QWWEKLFNWGRKNGCG	34,82	22,33	GWWDKLFNWGMLNMHM	32,92	28,62	GWWKKLFGYGCQNGHN	33,81
108	GWWEKLFNWGRQNQNA	62,30	17,60	GWWEKLFNWGRQNCHG	34,71	22,27	GWWEKLFNWGRQNQPM	32,31	28,09	GWWEKLTNWGMQDQPK	33,66
109	GWWEKLFNLGCQNQAM	61,90	17,49	SWWEKLFNWVRQFQDA	34,27	21,98	TWWEKLFNWGMLNNPK	32,14	27,94	GWWEKLFNFGMQNGLK	33,53
110	GWWEKLFNKVGQNQPA	61,75	17,44	RWWEKLFNWGRQNGHG	34,24	21,96	SWWEKLFCWGMRNMPD	32,01	27,83	SWWEKFFNWGCQNQPA	33,49
111	GWWEKLFNWGCQFQPQ	61,24	17,30	GWWEKLFNWGRQKQPA	34,16	21,91	SWWEKLFNWGRLNRMG	31,89	27,73	SWWEKAFNFGMQDQPK	33,33
112	GWWEKLFNWGMLNMHY	61,22	17,29	QWWEKLANWGRQNQHM	34,07	21,86	GFWEKLFNWGMLNKHD	31,01	26,96	CWWGKLFNWGMLDQSK	33,32
113	DWWEKLFNWGMLNMGG	60,90	17,20	GWWEKLYNWGMLNMGG	33,96	21,79	NAWEKLFNWGMENMHG	30,91	26,87	DWWEKLFNWGMQDQPY	33,31
114	AWWEKLFNAGRSNMHG	60,79	17,17	GWWEKLFNWGRDNQPM	33,92	21,76	SWWEKLVNWGYQDQPL	30,81	26,79	DWWEKLLSFGMQNGHN	33,21
115	QWWEKLFNAGRSNMHM	60,38	17,06	GWWEKLFMWGCVNMHA	33,58	21,54	SWWEKLFNMGMLNMPR	30,80	26,77	EWWEKLFKWGGQNQPA	33,11
116	GWWEKLFNWGRYNGCG	60,11	16,98	CWWEKFFNWGRQFQDD	33,58	21,54	GWWEKLFNWGRQYQQA	30,56	26,57	KWWDKLFLWGMQDQPK	33,02
117	GWWEKLFNWGRYNQPA	59,94	16,93	SWWEKLCNWGMLNMGG	33,31	21,37	SWWEKLFNVVGKNQFA	30,47	26,49	LWWEKLFNMGMQDQPK	33,00
118	GWWEKLFNWNRQEQPA	59,88	16,92	GWWEKFFNWGRQEQPA	33,23	21,32	SSWEKLFNWGMEDQLK	30,32	26,36	KWWEKLFYFGKQNTHM	32,92
119	GWWEKLFCWGRSNMHG	59,84	16,91	GWWEKLFNWRRQQQDG	32,88	21,09	YWWEKLLNWRCQSTTL	30,27	26,32	GWWEKLFNWVMQDQHM	32,13
120	GWWEKLFNAGMLNNHG	59,63	16,85	GWWEKLFNWGRDNRPE	31,97	20,51	SWWEVLFNWGRDNQLK	30,25	26,30	GWWEKLFNFGMLDSPK	31,23
121	GWWEKLFNWGMEAQPK	59,01	16,67	GWWEKLFCWVGQNQPA	31,72	20,34	NWWEKLFNWGMLLMGG	30,22	26,27	SWWEKLFGYGCQNGQL	30,76
122	RWWEELFNWGRQQQTA	58,95	16,65	GWWEKLANFGRQEQPA	31,45	20,17	MLWEKFFNFGCQNMTL	30,14	26,20	GWWDKLRNWGMNDQHM	30,09
123	GWWEKLFNWGRDNGHG	58,72	16,59	SWWTKLFNWGRQLQPA	31,28	20,06	KCWESLFNFGCQNMTL	30,11	26,17	GWWDTLFNWGCQNQKK	30,05
124	GWWEKLFNWGVLNMGG	57,74	16,31	GWWEKLFNWARQNQPV	31,19	20,01	GWWDKLFNFGRQLQPK	30,08	26,15	CWWEKLFNWVMQDQPD	29,56
125	GWWEKLFNWKMQNGHC	57,13	16,14	GWWEKLRNWGMQNGHG	31,10	19,95	SWWEFLFNWGRQNQLK	29,98	26,06	KWWEKLTNWGVQNTHM	29,52
126	GWWEKLFNWGAEDQPK	57,01	16,10	TWWENLFNAGRQNGCG	31,01	19,89	KWWEKLFNWGMLNMPK	29,63	25,76	SWWDKLFNFGMQNFPL	29,17
127	CWWEKLFNWGRDNGVG	55,29	15,62	GWWNKLFNFGCQNQHA	30,99	19,88	GWWEKLFNWGRQEQQA	29,46	25,61	GWWERLTNWGCQNTHM	28,53
128	GWWEKLFNGGMLNMGM	54,76	15,47	QWVEKLLNWGRQNQPM	30,85	19,79	KWWEKLLNWGYLNMGG	29,05	25,26	GFWEKLFNWVMQDQPK	28,52
129	GWWDKLFNWGYLNMHG	54,64	15,43	QWWEKLENWGRQNQPA	30,57	19,61	YWWEKLLLSGRQDQPK	28,76	25,00	SWWETVFNWGCQDQPK	28,19
130	SWWEKLMNWGRQNQPA	54,14	15,30	EWWEKLFNWGRQEQDA	29,36	18,83	GWWEKYFNWGRQDQPK	28,01	24,35	GWWYKLFNWGMQDGQG	27,87
131	SWWEKLFNWVRQNQPA	54,01	15,26	CWWEKLFNWGRQNQPG	29,19	18,73	GWWEKLVDWGRQNQRM	27,24	23,68	SWWEKLCNWGCQNQHM	27,59
132	GWWEKLFTWGRQNMHF	53,57	15,13	TWLEKLFNFGCQNDHM	28,95	18,57	SEWEKLFNWGMLNMPK	26,88	23,37	SWWDKLFLWGMEDQLK	27,35
133	GWWEKLFNAGRFNQPA	51,93	14,67	GWWEKLFNDGYQDQPK	28,68	18,39	KWWEKLLNQGRQQQDA	26,31	22,87	KWWEKFFYWGMMDQPK	27,10
134	SWWEKLFGWGMLNMHG	51,09	14,43	GWWENLFNWGMEDQPK	28,08	18,01	GWWEKFFEWGRQQQDT	26,02	22,62	SWWEELFNFGMQNQVA	26,86
135	GWWEKLFGEGMQNQPE	50,69	14,32	QWWEELLNWGMQSGHG	27,92	17,91	GWWERLFNWQRQDQPK	26,00	22,60	NWWECLFNYGCQNQHM	26,75
136	GWWEKLFNLACQNQPM	50,66	14,31	GWVEKLFNWGGDNGHM	27,89	17,89	GWWEKLFEWGMEDQLK	25,62	22,27	TWWEKLTNWGMENMTL	26,40
137	GWWESLFNLGCQEQPK	50,54	14,28	EWWDKLSNWGRQNQPM	27,39	17,57	CWAEKLFGFGCQNQHM	25,56	22,22	CWWEKLCNWGMQNGHN	25,90
138	GWFEKLFNWGRLNMHG	49,95	14,11	SSWEKLFNWGRQNQPM	27,13	17,40	DWWVKLFNWGRDNGMG	24,44	21,24	YWWEKLYNFGMQNCPK	25,67
139	GWWEKLFNKGMQNGHG	49,81	14,07	DWWEKLFNWGLQFQDA	26,98	17,31	GAWEKLFNWGRQDQPK	24,42	21,23	KWWRKLFNWGMLDQSK	25,55
140	YWWEKLFNWYMLNMPK	49,60	14,01	SWWLKLFNWGRQDQTA	26,96	17,29	GAWEKLFNWGRQNQPM	23,92	20,80	GWWEKLTNWGCQKQHM	25,05
141	GWWEKLFNWGRQNQGG	49,10	13,87	EWWEKLFNWLRDNGMA	26,77	17,17	GWWEKLFNEGCQCMHQ	23,82	20,70	NWWRKLFNWGMQNGHN	24,56
142	GWWEKLFNWQRQNQPD	47,53	13,43	QWWSKLLNWGRQNQPA	25,25	16,20	RWWEKLFNSGRGDQPK	23,16	20,14	SWWEKLFNWGMMRSRK	23,97
143	GQWEKLFNWGRQQQTA	47,01	13,28	GWWKKLFNDGMQNGHG	25,18	16,15	NWWEYLFKWGRQDQPN	22,77	19,79	KWWEKLFNDKMQNCPA	23,76
144	GWWEKLFNWKRQEQHF	46,94	13,26	GDWEKLFNWGRGNGHM	24,80	15,91	GQWDKLFNWGMLNKDD	22,50	19,56	GWWKKLFGYGCTNGQG	23,15
145	SWWEKLFNWVDQNQPA	46,59	13,16	EWWEKLFAWGMLNMHG	23,11	14,82	NAWEKLFNWGMLQMPK	22,19	19,29	GWWRKLFNWGCQNLTL	22,66
146	GWWEKLFNQGRTQQTK	46,30	13,08	EWVEKLFNWGRQEQPA	22,62	14,51	GWWEKLLNQGMLNMQQ	21,43	18,63	GYWDKLFNFGCQNQHA	22,59
147	DWWEKLFRWGRQNQPN	45,83	12,95	SNWEKLFNWGYQEQPA	22,07	14,16	SWWEELFNWGMLNMHQ	21,12	18,36	SWWEKLRNFGMMDQPK	22,21
148	GVWEKLFNWGYQNQGA	44,30	12,52	GDWEKLFNWGRQEQPA	21,19	13,59	KTWEKLFSWGCQNQHM	20,84	18,11	SWWEKLFFFGMQDQGK	21,98
149	GWWSKLFNWGRQNMHA	44,17	12,48	EKWEKLFNWGRDNGMG	21,12	13,55	CRWEKLFNWGMLTMHG	20,24	17,60	SAWEKLFNFGMMNGHN	21,58
150	GWWEELFNWGMQNGHG	43,74	12,36	GMWEKLFNWGMLNMGG	20,61	13,22	SWWMKLVNWGRQNQPA	20,22	17,58	TEWEKLFNWRMQDQSK	21,31
151	GWWEALFNWGRQGQPA	43,32	12,24	GWWEKLFNMGRTNGMG	20,15	12,92	GWWKKLFNQGMLNMHQ	19,92	17,31	GWWEKLFFWGCQNQHM	21,21
152	GWWEMLFNLGCQNQPM	42,68	12,06	GWWEKLFYWGRQNQPA	19,84	12,73	RTWEKLFNWGMQNQPA	19,66	17,09	TWWDYLFNWGCQNQHM	20,97
153	SWWEKLKNWGRQYQTA	40,63	11,48	GWWYKLFNWGRQQGCG	19,59	12,57	TWWEYLFNWGRQEQQA	19,54	16,99	GNWEKLFNWGGQNQVA	20,93
154	GWWEKFFNWARQNGHG	40,48	11,44	GWWEKLFNWEDQFQPY	19,35	12,41	TWWEEFFNWGMMDGMG	19,31	16,79	GWWETLFNWGMEDQLK	20,68
155	QWWLKLFNYGRQQQTE	39,65	11,20	GWWRKLFNWGRQNQPA	19,12	12,26	GWAEKLFNWGRDNGMG	17,74	15,42	EWWELLFNWGMNDQHA	20,17
156	GSWEKLFNWGRQNGHG	39,45	11,15	EWWELLFNWGCSNQPK	17,85	11,45	SWAEKLFNWGCSCQPK	17,00	14,78	CWWEGLFNWGMENMTL	19,03
157	GWWEKLYNWGRLNMHG	39,34	11,11	GWWLKLFNWGRQEQGA	17,71	11,36	GWWETLFNVVGQNQPA	16,79	14,59	AWWNKYFNDGCQNTHM	18,06
158	SSWEKLFNWMRQDQPA	36,57	10,33	GWWEKLENWGYQDQPK	17,25	11,06	TWWELLFNYGMLNMHG	16,57	14,40	NWWEFLFNFGMQNTHM	16,78
159	GWWEGLFNWGRQNQPF	36,43	10,29	GWWEKAFNWGYVNMHA	16,99	10,90	YWWEGLLNWGRQQQDG	16,55	14,39	GWWTNLENWRFQFFPK	16,74
160	GWWEFLFNWGRSNMHM	35,55	10,04	GNWEKLFCWKCVNMHA	16,13	10,34	GWWEDLFGFGFQNQHM	16,35	14,21	VWWEKLTNWGMQCCPA	15,73
161	GWWEKFFGWGMLNMTA	35,08	9,91	SWWEVLFNWGYQDQPK	13,79	8,85	SEWEKLFNWGMLRMPK	16,11	14,01	NWWLKLFNWGCQNLFL	15,46
162	GWWRKLFNWGRQNGCG	29,30	8,28	TWWEYLFCWGRQNQPA	13,13	8,42	SWWEKLRNVVYVDQPK	15,99	13,90	GWWECLFNDGFLNMHG	15,22
163	GWWEKAFNWGRQNGCG	29,25	8,26	QWWYKLFAWGRQNQPA	13,09	8,39	RWFEKLLNWQRQNQPA	15,79	13,73	GWWDKLFNAGMEDQLA	14,62
164	GWWEKLRNWGRQFQPA	29,17	8,24	GWWELLFNLGCQNQPQ	13,05	8,37	SWWYKLFNVVCSCQPK	15,76	13,70	NWWETAFNWGVQDQPK	14,56
165	KGWEKLFNWGRQNQPA	28,91	8,17	QWWEKSFNWGRQNEPA	12,98	8,32	SWWEKNFNWGRQNQHM	15,18	13,20	CWWEKLFLWGMLDQPK	13,97
166	GWEEKLFNWSMLNNHG	27,42	7,75	GWWEKLENWGCVNMHA	12,86	8,25	YWWFKLFNWGCSCQPK	14,41	12,52	GDWEKLFNWGCQNQHM	13,49
167	GWYEKLFGWGYQNMHF	26,66	7,53	CKWEKLFCWGRQQQTA	12,66	8,12	SWAETLFNWGRDNGMN	13,60	11,82	GWWEKLFLFGVQNGHN	12,14
168	GWWEFLFMWGRDNGHG	23,83	6,73	CKWEKLFNWGMQKGHM	12,49	8,01	SAWEKLFAWAMLNMQA	13,52	11,75	GDWEKLFNWGMQDQPK	11,85
169	GWSEKLFNWGMQNGHG	22,28	6,29	GWWEKLFVWGMLNMRG	12,24	7,85	SWWEKLMNFGCQNMTL	12,34	10,72	CWAEKLFGYGCQNGQG	11,81
170	GWWVKLFNWGRLNMHG	22,18	6,27	GWGEKLFNWGRQEQPA	11,26	7,22	DWKEKLFNWGRQDRHN	11,55	10,04	RWLVKFFDVGMMESPK	10,56
171	SWWEKLYLLGCQNQPM	22,13	6,25	GWTEKLFNFGRQQQNA	11,21	7,19	SWWEKLDNWGCACQPK	10,76	9,35	GWWDGLFNWGMEDQPA	9,91
172	QEWEKLFMWVGQNQPA	22,13	6,25	GWWEKFDNWGMQNGHG	11,07	7,10	GWWVKLVNWGYQDQPK	9,81	8,52	GWWEKSFNWGCQNLTL	8,45

173	GRWEKLFNWGRQNGHG	19,98	5,64	SWWEKGFNWGCSNQPK	10,87	6,97	GWNEKLFNWGMLNMHG	8,32	7,23	GWWDKSFNWYCQNLTL	6,98
174	GWWEVLFNWSRQEQPA	18,58	5,25	GWYEKLFDWGMDNGMG	10,32	6,62	SWWEKCFNWRRGSVMG	7,29	6,33	CWWCKLRNWGMVAQST	6,19
175	GWWEKRFNWGMLNNHG	16,90	4,77	GWQEKLFNWGRQQQPK	10,25	6,58	CWWEKMFNWGCSEQPK	6,33	5,50	GWWEKAFFWGMNDQHM	5,70
176	GWWEVLFNWGRQEQPA	16,50	4,66	GWWEKGFNWGRDNGHM	10,14	6,50	TWWEKSFNWGYDNGMG	6,32	5,49	CMWEKLFNYGGQNGKR	4,96
177	GWWEKMFNWGNLNMHG	13,08	3,70	GWKEKLFNWGMLNMHG	9,65	6,19	GWQDKLFNWGCSCQPK	6,16	5,35	GWWEKNFNWGMQEQPK	4,89
178	SWKEKLFNWGRDNGHG	12,87	3,63	GWWFKRFNWGMLNMGG	9,52	6,11	SWGEKLFNVVKQNGHN	5,71	4,96	GWWEKFSNQGMMDSPK	4,56
179	SWNEKLFNWGRQFQCA	11,89	3,36	GWWYKMFNWGRQEQPA	8,10	5,20	CFSEKLFQWGMLNDHD	5,57	4,84	KWKEKFFNWGMQNGHN	4,56
180	GWKEKLFNWGRQFQPA	11,47	3,24	CWWEKMFCWGMLNMHG	6,39	4,10	RWWEKLSNEGRQNQPA	5,36	4,66	TWWYKLENFGSQNGHN	4,40
181	GWGEKLFNWGRQCQPA	10,86	3,07	GWWEVEFNWGMLNMHG	6,32	4,05	DWWEKRFSWGRLNMGG	5,18	4,50	YWWEKLANVGAQNCPF	4,32
182	SWWEKQFNWGRQNQPA	9,00	2,54	SWNEKLFNWVGQNQPA	6,14	3,94	TWWEKKFNWGRQGQPA	4,90	4,26	KWWEKAFNDVMQDQPK	4,21
183	GWEYDLFNWGALYVHQ	8,89	2,51	EWGEKLFRWGRQFQDA	5,89	3,78	CWWELEFNWGMLNMPK	4,67	4,06	NWGMRLFNWGTENMMG	4,08
184	GWWEKDFNWGRQNMHG	7,94	2,24	QNWEKLFLWVGQNQPA	5,60	3,59	SWTEKLVNWGYQDQPK	4,53	3,93	SWWQKRFNFGMQNQPK	4,02
185	GWWEKDFNWNMLNMHM	6,83	1,93	GWNEKLFNWGCSNQPK	4,87	3,12	DWWEKNLNWGRQQQDA	3,85	3,35	TWNEKLFNWGCQNQHM	3,25
186	GMWEKLGNWGRSNMHM	6,27	1,77	EWKEKLFNWGCVNMHA	4,66	2,99	GWWEKKFNWGCQNMTL	3,79	3,30	NWWDKKFNWGMEDQLK	3,03
187	CWWEKLCNKGMQNGHG	5,77	1,63	CLWEKRFNWGRQQQHA	4,26	2,73	TWNEKFFKWGRQQQDA	3,77	3,27	GWWEKFTNDGMMDSPK	3,02
188	GWWEKMFNWVGQNQPA	5,39	1,52	GWWEKLNNGGRQNGPA	4,11	2,63	KWWNKEEMWGMLKMVL	2,82	2,45	CWDEKLFNWGMLDQSK	2,62
189	CWNEKLFFWVGQNQPA	5,00	1,41	GTWRKLRNWTCQVQPK	3,01	1,93	RWCVKTFSLGRQDQPT	2,48	2,15	SWWEKDFNWGMMDQPK	1,96
190	RGSCKLFNWGREGGCG	3,17	0,89	EWNEKLFNWGRDNMKG	2,54	1,63	GWWETMFNWGMMDQPK	2,32	2,01	SWWEKMFNFMMQNGHM	1,88
191	GWWQFMFNMGMCFMEM	2,17	0,61	GFQEKLFNLGCVRMHA	2,48	1,59	GWWEKTFNDGRDNGMG	2,04	1,77	SWCEKLLGWGMMDQPK	1,88
192	GWQEKRFLWGNLNQPA	2,12	0,60	GWDEKLFNWGMQNGLG	1,65	1,06	TKWEKRFCTCMMDRAK	1,95	1,69	TWWKKNLNFGAQNCMA	1,49
193	GTCEKLLNVGVLYMGG	1,86	0,53	MQWEKVFRKGVQNGCG	1,52	0,97	GYTEKLFNWGMLNMGG	1,78	1,55	KWEEQLFQWGYQDQPK	1,48
194	GWWEKLENMGMLNMHM	1,64	0,46	TRWEKLMNWGMQNGHG	1,45	0,93	NKWEKLFNAGMEDQTL	1,21	1,05	GWWEKKCNWGCQNQRM	1,42
195	GWNEKLFNAGRQDQPK	1,09	0,31	GWGEKLFNMGMQNGHG	1,06	0,68	VDWDKSFNFQCQVQHM	1,11	0,96	GWWEKEFNWGMMDSHM	1,32
196	QWWEKKAMSGRQDQST	0,67	0,19	GWWEKEFNGGMQNGHN	0,87	0,56	TRWEKAANWGCLNMPF	0,86	0,74	TDWEKLRTWGCQNQPK	1,20
197	QWNEKTFNWGRQTVED	0,50	0,14	SWVEKSCNWGRQNQEA	0,26	0,16	KQEFFLMRDSMLNMHG	0,39	0,34	TWWEKDTNWGCQNGHN	0,76
198	CYWEKENNLGRMNGCN	0,45	0,13	EWDEKSFNWGRQEQPA	0,21	0,13	TWDETLFNSGRQDQPK	0,16	0,14	KWCEKLFNDGFLNCPA	0,55
199	GWEEKLKACGQAVQTC	0,26	0,07	CFWEKDGNWGMEEGKG	0,19	0,12	GEWDKSFCFVCQAMSM	0,14	0,12	NWEEKLESWGGNRQHM	0,35
200	QGTEKLSNMYRQNQPA	0,16	0,04	VECEKFEGWTRQEQNA	0,07	0,04	SLCEKFLNQGMLNDTQ	0,03	0,03	GQTDRLQAWGMESQTK	0,08
	**Lead from Gen6**	**x**	**c**	**Lead from Gen7**	**x**	**c**	**Lead from Gen8**	**x**	**c**	**Lead from Gen9**	**x**
	GWWEKLFNWGMLNMHG	120,47	34,03	TWWEKLFNWGRQEQPA	58,55	37,56	GWWDKLFNFGCQNQHM	52,83	45,93	SWWEKLLNWFRQNQPA	73,57
	GWWEKLFNWGMLNMGG	108,32	30,60	GWWEKLFNFGCQNQHM	52,98	33,99	NWWEKLFNWGMEDQLK	50,60	43,99	SWWEKLFNFGMQNCPA	66,76
	GWWEKLFNWGMQNGHG	102,86	29,06	SWWEKLFNWGMLNMHG	51,34	32,93	TWWEKFFNWGMMDQPK	49,71	43,22	NWWEKLFNWGMNDQHM	66,27
	GWWEKLFNWGRSNMHM	102,72	29,02	GWWEKLFNWGMLNMGG	50,88	32,64	KWWEKLFNWGMLNMHG	48,42	42,10	SWWEKLFNFGMQNTHM	64,80
	QWWEKLFNWGRQNQPA	96,41	27,24	GWWEKLFNLGCQNQPQ	46,66	29,93	GWWEKLFGFGCQNQHM	47,31	41,13	KWWEKLFNDGFLNMHG	62,89
	GWWEKLFNWGRDNGHG	93,71	26,47	QWWEKLFNWGRQNQPM	45,64	29,28	GWWEKLFNWGMQDQPK	47,23	41,06	GWWEKLFNWGMQDQPK	62,26
	GWWEKLFNWGRQQQTA	88,02	24,87	SWWEKLFNWGRQNQPA	45,57	29,23	GWWEKLFNWGMLNMGG	46,57	40,49	SWWEKLFNWGCQNLTL	59,69
	CWWEKLFNWGRQNGCG	86,07	24,32	SWWEKLFNWGRQDQPK	45,27	29,04	SWWEKLFNWGRQNQPM	42,75	37,17	GWWEKFFNWGMMDSPK	58,11
	GWWEKLFNWGRQNQPA	84,76	23,95	SWWEKLFNWVGQNQPA	43,46	27,88	GWWEKLFNFGCQNMTL	42,59	37,03	GWWDKLFNFGCQNQHM	54,15
	GWWEKLFNWGRQFQPA	84,50	23,87	CWWEKLFNWGMQNGHG	42,82	27,47	SWWEKLFNFGMQNGHN	42,43	36,89	KWWEKFFNWGMMDQPK	52,79
	GWWEKLFNWGNLNMHG	77,39	21,86	GWWEKLFNWGRQQQTA	42,39	27,19	SWWEKLFNWGRDNGMG	42,03	36,54	TWWEKLFNWGCQNQHM	52,74
	GWWEKLFNLGCQNQPM	69,17	19,54	GWWEKFFNWGMEDQPK	42,23	27,09	RWWEKLFNWGMQDQPK	41,84	36,38	GWWEKLFNWVMQDQPK	47,55
	GWWEKLFNWGCSNMHA	67,82	19,16	GWWEKLFNWGYQDQPK	41,43	26,58	CWWEKLFNWGMLNKHD	41,34	35,94	TWWEKLTNWGCQNTHM	45,33
	GWWEKLFNWGREDQPK	63,63	17,98	GWWEKLFNWGRQFQDA	40,58	26,03	GWWEKFFNWGCQNQHM	38,66	33,61	GWWEKLFGYGCQNGQG	44,99
	SWWEKLFNWVGQNQPA	62,84	17,75	EWWEKLFNWGRDNGMG	40,21	25,79	DWWEKLFNWGRQDQPK	38,62	33,58	TWWEKLFNWGMQDQPK	44,93
	GWWEKLFNWGRQEQPA	62,81	17,74	GWWEKLFNWGRQNGCG	40,16	25,76	SWWEKLFNVVGQNQPA	38,36	33,35	SWWEKFFNWGCQNQHM	43,66
	GWWEKLFNWGRQDQPK	62,51	17,66	GWWEKLFNWGCSNQPK	39,82	25,54	TWWEKLFNWGRDNGMG	38,18	33,19	GWWDKLFNWGCQNQHM	40,74
	GWWEKLFNWGRQEQPA	61,69	17,43	EWWEKLFNWGMLNMHG	38,90	24,95	CWWEKLFNWGCSCQPK	36,50	31,73	CWWEKLFNWGMLDQSK	39,11
	SWWEKLFNWGRQNQPA	53,62	15,15	GWWEKLFNWGRDNGHM	35,05	22,48	SWWEKLFNWGMLNMPK	35,58	30,93	SWWEKLFNFGMQNGHN	38,71
	GWWEKLFNWGRQNGHG	53,19	15,03	GWWEKLFNWGCVNMHA	34,89	22,38	TWWEKLFNSGRQDQPK	34,42	29,92	GWWEKLFQWGMQDQPK	37,85
	GWWEKFFNWGMLNMHG	49,06	13,86	QWWEKLLNWGRQNQPA	33,88	21,73	GWWEKLLNWGRQNQPA	33,74	29,33	NWWEKLFNWGMENMTL	36,77
				GWWEKLFNWGMQNGHG	33,81	21,69	SWWEKLVNWGYQDQPK	30,04	26,12	GWWEKLFNFGMQNGHN	35,55
				CWWEKLFCWGRQNQPA	33,31	21,37	SWWEKLFNQGMLNMHQ	29,14	25,33	GWWDKLFNWGMEDQLK	33,84

D-peptide sequences

	Generation 1	x	c	Generation 2	x	c	Generation 3	x	c	Generation 4	x	c
1	**tnhylqwfagparhfd**	83,25	0,60	tnhywkwfwgqlerkd	205,07	1,25	tnhyllwfagqlerkd	33,35	3,78	asqrlqwwagalrkfl	23,48	5,00
2	**epqrltwfwgqlerkd**	69,63	0,50	apqrwqwfagalrrfd	196,67	1,20	tnqrlqwfagalrhfy	31,88	3,61	tnkkewifygalrqfy	19,36	4,12
3	**apqrlqwfagalcrwv**	67,18	0,48	apqwlqwfagplrrfd	165,11	1,01	ecqrlqwfagalrhfy	29,77	3,37	tnkqwnwfagalrhfy	15,83	3,37
4	**apqrlqwfalfdhnah**	65,51	0,47	epqrltwfwgqlrhfd	157,06	0,96	asqrlqwfagalrhfy	28,45	3,22	tnqrldwfagalrhfy	15,58	3,32
5	**hkprlewfagplrqfy**	59,21	0,43	ltgrltcfwgqlerkd	133,46	0,81	vgqkwnfiylflcrwv	21,68	2,45	tnnrlqwfvgalwhfh	15,49	3,30
6	**ligtlqwflgplrrfd**	55,71	0,40	tnhyllwfagparhfd	127,89	0,78	tnkkwnfiycplrqfy	19,50	2,21	qnhylwifagalrhfd	15,36	3,27
7	**drhrlqwftgplrhfd**	48,88	0,35	tnhylqwfagparhfd	112,72	0,69	lnhylswfagparkfl	16,72	1,89	tnwylwwfvgalrhfh	15,27	3,25
8	**tnhyewifsytmaiqp**	46,96	0,34	anhylqwfagpwtkhd	110,86	0,68	tnhyllwfagpnrrfl	16,72	1,89	aqwwlqcfagalrhfy	14,83	3,16
9	**aplllqwfagplrrfd**	46,47	0,33	tnhylswfagparrfl	109,05	0,67	telyewifagalrqfy	15,94	1,80	vgskwnffagalrhfy	14,71	3,13
10	**apqrlqwfagplrhfq**	46,20	0,33	elvrwswfwcqlnrkt	108,87	0,66	tmhkllwfagllrrfd	15,68	1,77	thhyltwfagalrhfy	14,55	3,10
11	**dhqrlqwfanplrrfl**	45,91	0,33	epqrlqwfalfthnah	108,64	0,66	ppqrlqwfvgalrhfh	15,28	1,73	tnhywlwfagqlrhfy	14,39	3,06
12	**apkrlqwfagahdlts**	45,72	0,33	tnhylqwfagparhfh	103,16	0,63	apqrlqwfycplrqfy	15,09	1,71	tnhyllwfagalrhfs	14,20	3,02
13	**lrgrlqwfagealrfd**	45,60	0,33	apqrlqwfagalcrwv	102,27	0,62	cthylvwfagalrnfd	14,54	1,65	tnhylqwfagalrhfy	13,46	2,87
14	**plfglqwfagplrrfd**	44,15	0,32	ppqrlqwfagalrhfy	101,55	0,62	lttrltifagalcrwv	14,44	1,63	tnqtlqwfagalrhfy	13,25	2,82
15	**frgisdifsyleslag**	44,08	0,32	apqhnwlymalqlwfg	100,26	0,61	tnhrlqwfavfyhnah	13,92	1,58	lnhalswfagalrhfy	13,18	2,81
16	**apqrlqwfaytnslag**	43,94	0,32	ftgisewfagplrqfy	99,78	0,61	apqwlqcfalflcrwv	13,38	1,51	lnhylswfcgalrhfy	12,97	2,76
17	**tnhywkifsmaiqpea**	40,14	0,29	cnhyltwfwgqterkd	99,62	0,61	thhylswfagparrfl	12,96	1,47	tnhrlnwfalfycrfh	12,91	2,75
18	apqrlqwfsytcssag	38,84	0,28	dhqrlqwfagvltrwv	99,59	0,61	qnhylwifagalcrwv	12,73	1,44	tnkkwnwvagalrhfy	12,88	2,74
19	fikdwlthiqtnhyag	37,96	0,27	wgkkwnfiycplrqfy	99,02	0,60	apqrlqwfalflcrqv	12,59	1,42	tnkkewifagalrqfy	12,86	2,74
20	tnhyakilshytllwg	37,89	0,27	apqrlqwfalflcrwv	98,87	0,60	tnhrlnwfalflcrwv	11,95	1,35	tnyylvwfagalrnfd	12,72	2,71
21	apqrlqwfagpyrrfd	37,46	0,27	tqlyewifagalcrwv	98,43	0,60	lthylqwfagparhch	11,76	1,33	ecqrtqwfagalrqfy	12,43	2,65
22	apqrlqwfagplrtfh	36,83	0,27	ppqrlqwwcwwmfwdn	97,20	0,59	apqrlqwfalflnrkt	11,56	1,31	lnhylmwfagparhfy	12,43	2,65
23	apqrlqwrvlfghrah	33,87	0,24	apqrlqwfasawrrfd	96,56	0,59	apqrlkwfagplrqfy	11,33	1,28	lthylqwfagalrhfy	12,30	2,62
24	apqrlqwfagplrrfm	32,81	0,24	apmrlqwfwgqlerkd	96,50	0,59	tlhylqwfagplrrfd	10,89	1,23	vgqqlqwfagalrhfy	12,27	2,61
25	plfghrwflgpmrrfd	31,62	0,23	tnqrltwfwgqlerkd	96,12	0,59	anhyllwfagpanhfd	10,67	1,21	tnhyslwfagaqrhfy	12,26	2,61
26	arhrlqwfagplrrfd	31,62	0,23	ligtlqwftgplrhfd	94,79	0,58	tnhylqwfalfthnay	10,51	1,19	vnhymlwfagmlerkd	12,05	2,57
27	pigtercrplfevrwh	30,38	0,22	apqrlqwfagtlcrfd	94,63	0,58	egkwwnfiycplrqfy	10,50	1,19	apqrlqwfagalrnfy	12,03	2,56
28	tnhyekifsytlrrfd	30,11	0,22	tnhylqwfwgqlprkd	93,85	0,57	epqrlqwfalflcrwv	9,28	1,05	tnqrlqwfagalrnfd	11,96	2,55
29	apqtlqwfasprhrpl	29,97	0,22	ttqrltwfwgqlerkw	92,62	0,56	tnhylsdfagparrfl	8,82	1,00	anqrlqwfagalrhfy	11,63	2,48
30	drhpnlfghrallrwv	29,30	0,21	tnhylwwfpmpsahfy	91,66	0,56	ltgrltcfwgqlerkk	8,28	0,94	tthyllwfaglsrrfd	11,60	2,47
31	qpqrlqwfagplrrfd	27,96	0,20	apqrlqwfagalcrcd	89,46	0,55	apqrltwfagparrfl	7,99	0,90	lnqrlqwfagalrhfy	11,57	2,46
32	pefglewfagplrrfd	27,80	0,20	apqrlqwfagalrrfd	88,78	0,54	hpqrlqwfwlfthnnh	7,96	0,90	knhtlqwfagalrhfy	11,56	2,46
33	apqrlqwfagplrrfd	26,89	0,19	apqrlqwfagalrrfd	87,31	0,53	dlqrlqwfagalcrwv	7,45	0,84	tlhylqwfagalrhfw	11,55	2,46
34	fikdnnsfppallrwv	25,98	0,19	apqrlqwfagalrrfd	85,70	0,52	anhylqwfygpwrrfd	7,42	0,84	tnqrlqwfagalrhfy	11,52	2,45
35	epqrlqsfppallrwv	25,98	0,19	tnhylqwfagplrqfy	85,68	0,52	apqrlqwfasacrrfy	7,17	0,81	cthylvwfagalrhfl	11,46	2,44
36	apqrlqwfhglrahdm	25,72	0,19	tnhyewnfslfdhnah	84,87	0,52	tnhyllwfagpardkd	7,12	0,81	asqrlqwfagalrqfy	11,21	2,39

37	awcptqwfagplrrfm	25,67	0,18	apqrlqwfagalriqp	84,44	0,52	tnhryqwfagalcrwv	6,94	0,79	asqrlqwfagalrsfy	11,18	2,38
38	apqrlqwfagpsrral	25,67	0,18	lrqrlqwfanllrrfl	84,11	0,51	fqgisvwfagplrrfd	6,85	0,77	ecqrlqwfagalrqfy	11,18	2,38
39	apqhlqwmaytmrrfk	25,45	0,18	apqrlqwfalwmfwdn	83,49	0,51	ehqrlqwfagvltrwv	6,81	0,77	evdqsqwfaealrmfy	11,17	2,38
40	lrgckkilsyleslag	25,22	0,18	apqrlqwfalwmfwdn	83,44	0,51	dhqrlqwfagvltrwv	6,69	0,76	pnqrlqwfvgalrhfy	11,11	2,37
41	drqrlqwfagplrnfd	25,17	0,18	aphylqwfapkarhfd	82,83	0,51	tnhylqwfaclacrkd	6,47	0,73	apqrlqwfagalrhfy	11,09	2,36
42	tnhyekwfagplmrfd	25,09	0,18	ldlayikfygalrrfd	81,41	0,50	snhywkwfagvltrwv	6,34	0,72	tnhyllwfagqlerkd	10,88	2,32
43	eahrqvwfagplrrfd	25,06	0,18	apqrlqwfaytnswfy	81,29	0,50	anhylqcfagparhfd	6,24	0,71	tnqrlqwfagalrhty	10,75	2,29
44	gtdrhrwfagplrrfd	24,83	0,18	slfgeqwfagalcrwv	78,26	0,48	tnhylqwfaghltrwv	6,02	0,68	tnkkwntiagalrhfy	10,67	2,27
45	fikdylpfppallrwv	23,78	0,17	higvlqwflgplrrfd	78,04	0,48	tnhywkwfagalrrfd	5,97	0,68	qsqrlqefagalrhfy	10,62	2,26
46	apqyykifsyteslvg	23,77	0,17	lrqrhqwfagalcrwv	77,21	0,47	apqrwswfayqlrhfd	5,96	0,67	asqrlqwfagalrhfy	10,59	2,25
47	apqrlqwfagprahdm	23,69	0,17	lpqrlqwfagalcdwv	76,79	0,47	apqrwqwfalalrrkd	5,78	0,65	aserlwifagalcrwv	10,55	2,25
48	drhrplfrplfthrah	21,32	0,15	tnhylwifsytmaikp	76,52	0,47	vtgikewfagplrqfy	5,60	0,63	telyhwwfagqlerke	10,36	2,21
49	frgisdligtdrhrql	21,24	0,15	tnhywkifsmplrhfq	76,16	0,46	tnhywklfagptrrfd	5,52	0,62	apqrnqwfalflcrwn	10,33	2,20
50	fiknnlthilfghrah	21,20	0,15	epqrltwfwgqlerkd	75,98	0,46	apqllpwfagparrfl	5,37	0,61	tnhyslwfagqleykd	10,32	2,20
51	lrvcpqwfagplrrfd	20,44	0,15	apkrltwfwgqlerkk	74,54	0,45	apqrlqwfagparhfn	5,14	0,58	tcqrlqwfagalrhfy	10,31	2,20
52	tpqrlqwcagpywyfe	20,35	0,15	qnhyldwfsyleslhg	73,97	0,45	alvrwswfwchlnrkt	5,13	0,58	tnhyllyfagqlcrwv	10,29	2,19
53	kigtdrhrplfghral	20,01	0,14	apqrlqwfagaqrrld	73,81	0,45	tnvrwstfwcqynrkt	4,95	0,56	cthylwifagalcrwv	10,28	2,19
54	apqylqwfahdmtsat	19,34	0,14	hkprlewfagplrqfw	73,77	0,45	ttgdsewflgplrqfy	4,94	0,56	tnhyylwfalpnrrfl	10,27	2,19
55	apqrlqlrgyfmaiqp	19,16	0,14	wgkkwnfiycpcdlts	73,72	0,45	apqwlqwfaghlrrfd	4,71	0,53	anhysqwfasalrhfy	10,23	2,18
56	aaqrlqwfhrahdmts	18,95	0,14	ldkayikwcgalcrwv	72,14	0,44	apqhqwlymalqlwfh	4,65	0,53	tnqrlqwfagalrhft	10,21	2,17
57	tnhyekiffghrayda	18,69	0,13	tnhylqwfanplrrfl	71,79	0,44	tpqrlqmfalfphnah	4,60	0,52	mmhkllwfagllrrfd	10,20	2,17
58	mhmklkifsyteslan	18,57	0,13	hkprlewfalfdhnmh	71,44	0,44	epqrwqwfklalrrfd	4,60	0,52	ecqrlqwfagalrhfy	10,17	2,17
59	apqrtqwfagprhrpl	18,27	0,13	livylcwflwdlrrhd	69,77	0,43	dhqrlhwfagalcrwv	4,59	0,52	esvrlqwfagalrhfy	10,05	2,14
60	lrglfmaghkahdmts	18,21	0,13	plfgltwfagplrrld	68,32	0,42	llvrwswfwcqlnrkt	4,50	0,51	ppprlqwfvgalrhkd	10,01	2,13
61	drhrplffagplrrfd	17,56	0,13	aphylqwfagparhfd	67,92	0,41	lnhylstfwgqlerkd	4,41	0,50	tnhyllwfagplrrfd	10,01	2,13
62	drhrplfghrplrrfd	17,23	0,12	hnhylqwfagparhfd	67,80	0,41	tnhhnwlymalqlwfg	4,40	0,50	tlhylqwfagalrhfy	9,96	2,12
63	aplrlqwfagnmtsat	16,83	0,12	apqllqwfasplrrfd	67,77	0,41	elvrwqyfagplrrfd	4,26	0,48	lnhyllwfagparkfd	9,92	2,11
64	frgiseliggplrrfd	16,48	0,12	lrgrlqwfagparhfd	67,07	0,41	tqpyltwfwgqmrhfk	4,19	0,47	ecqrlqwfagalrhfy	9,89	2,11
65	apqrlqwfagtsatal	16,37	0,12	lpqrnwlymalqlafg	64,78	0,40	apqrlqwfagalchfd	4,15	0,47	lncrlqwfagalrhfy	9,87	2,10
66	plqrlqwqvgplvrfd	16,27	0,12	wgkkwnfiycpcypda	64,56	0,39	tnqrlqwfagalrtfd	4,02	0,45	tnsrlqwfagalrhfy	9,79	2,09
67	plfghrahdytlrrfd	16,17	0,12	lrgrlqwfngeaerkh	64,33	0,39	ltgrltwfasawrrqd	3,87	0,44	tnvyllwfagqlpkfl	9,77	2,08
68	frginlthimtyhyea	16,12	0,12	tnhyetwfwgqlerkd	64,29	0,39	epqrlqwfalfterkd	3,87	0,44	vthyllwfagpnrrfl	9,62	2,05
69	lpqrlqeflgplrvec	15,92	0,11	apqrlqwfvlfdhlah	64,22	0,39	dhqrlqwfagvlprwh	3,87	0,44	lttrltifagalrhft	9,60	2,04
70	vkmhmnwfagdvrrfd	15,69	0,11	lrgrlqwfapearhfd	63,71	0,39	apqrwswfwcqlnrkt	3,86	0,44	eyqrlqwfagalrhfy	9,57	2,04
71	drhrpqwfegplrrfd	15,37	0,11	frgrlqwfagealwdd	62,99	0,38	ktyvlqwtagllrhpw	3,72	0,42	tnqreqwfagalrhfy	9,54	2,03
72	gtdrhrhrplfghrah	15,17	0,11	hkprlewfagplkhfd	62,92	0,38	tnhylswfagparhfd	3,64	0,41	kpqrlqwfalflchfy	9,48	2,02
73	gtdqhrptfghrrrfd	14,73	0,11	apkhnwlymalqlafg	62,79	0,38	tnhnlqwfagalcrwv	3,62	0,41	qnhylvifagalrhfp	9,40	2,00
74	gtdrdrhrplfghrah	14,73	0,11	apqrlswfaytnslag	62,62	0,38	tnhywkwfwgqlrrfd	3,60	0,41	thhyllwfagqlqrkd	9,40	2,00
75	lpqrlqwfagplrefd	14,31	0,10	lskhnwwfvgplrrfd	61,96	0,38	apqwltpfyyplmrfm	3,60	0,41	vckrlqwfagalrhfy	9,34	1,99
76	prhrplfghrahtsat	14,21	0,10	tnqylqwfagparhfd	61,48	0,37	tnhyakwfwgqlrhfd	3,53	0,40	thhylswfagparhfy	9,29	1,98
77	drhrvlffagahdmtd	14,19	0,10	wgcknnfiycylcrwv	61,43	0,37	plvrwswfwcqlnrkt	3,50	0,40	hgnywnfiyqfkcrwv	9,24	1,97
78	apqrlqhrplfhhrah	14,12	0,10	lnlayikkclwmfrwv	60,69	0,37	tnhywkwymalqlwfg	3,43	0,39	qghyllwfagqlerkd	9,23	1,97
79	plfrplfghrahdmts	13,99	0,10	wgqrlqwfalqdhnah	60,67	0,37	elvrlqwfagparhft	3,43	0,39	tphyllyfygqnrrhl	9,18	1,96
80	gtdrhrplfghryhdm	13,96	0,10	aplllqwfagdlcrwv	60,09	0,37	elvrtswfwcqlnhfd	3,32	0,38	tmhyllwfagqlerkd	9,12	1,94
81	tigtdrhrplfghrah	13,92	0,10	apqrlqwfalfdhnad	59,72	0,36	epqrltwfwgqlrrkd	3,30	0,37	apqrsqwfalflcrwv	8,99	1,91
82	tnhyekifsyteslpl	13,83	0,10	epqrltwfwgqlests	58,28	0,36	apqrwlwfagplrrfd	3,30	0,37	atqrlqwfygalcrwv	8,96	1,91
83	apqrlqifsyteslal	13,67	0,10	dhqrnwlymalqlafg	58,07	0,35	epqrlcwfwgqlrhfm	3,29	0,37	ecqrlqwfagalrhfy	8,92	1,90
84	mhmnlrthiqtwhyea	13,12	0,09	adlllqwfagahdlts	58,01	0,35	mpqrwqwfagalrrfd	3,28	0,37	asqrlqwfagalrhfy	8,70	1,85
85	gtdrhrplfghrahdg	13,02	0,09	apkrlqwfagahdlfd	57,41	0,35	ppqrlqwhagslrrfd	3,25	0,37	tnhylswfagpanrql	8,63	1,84
86	tnhyekifsyteslal	12,81	0,09	nnprlewfagplrlfy	57,12	0,35	tnqwlqwfkgplrrfd	3,19	0,36	tnhrlqwfagalrhly	8,54	1,82
87	hrplfdligtdrhrpa	12,38	0,09	aplllqwfagplrrfd	56,37	0,34	anhylqwfagparhqd	3,19	0,36	tnqrlqwfavfyhnan	8,49	1,81
88	apllrnyfsyteslag	12,09	0,09	plfglqwyagparhfd	55,66	0,34	tnhylqwcagparhfh	3,09	0,35	vgqkwnfiylflcrwv	8,49	1,81
89	apqrlqwnagplhrpl	12,07	0,09	apqrlqwfaytnslag	54,86	0,33	ppqrlqwfagalrhpd	3,07	0,35	tnqrllwfagllrrfd	8,48	1,81
90	arhralfghrahdmts	12,06	0,09	hkprlewfagplrqah	54,59	0,33	tpqhnwlyealqlwfg	3,04	0,34	asqrlqwfvgalrhfh	8,45	1,80
91	lnhyeyefsyteslag	11,95	0,09	lpkrlqwfagahlrfm	54,55	0,33	apmrlqwfwgplrrfd	3,00	0,34	qnhylwifagasrqfy	8,43	1,80
92	mhmnlrplfghrahdm	11,89	0,09	ligylqwflgphdcts	53,56	0,33	ahqrltcfwgqlerkw	2,99	0,34	thhyllwfagldrrfd	8,28	1,76
93	mwmnlrgcfmaimpem	11,69	0,08	apqrlqwftgmlrhfd	52,55	0,32	apmwlqwfagalrrfd	2,94	0,33	tahyllwfagqhcrwv	8,25	1,76
94	shmnlrgfagplrrsd	10,34	0,07	lsprlewfagnlrqfy	52,50	0,32	apqrlqwftdparhfh	2,88	0,33	tnqrlqwfcvfyhtah	8,21	1,75
95	nrhrplfghrahdmss	10,05	0,07	tnhylqwyanplrrfl	52,24	0,32	epqrltwfwgqlrhfd	2,85	0,32	sgqkwnfiylqlerkd	8,16	1,74
96	apyrplfghrahdmts	9,92	0,07	apqylqwfttparhfd	52,11	0,32	apqwlqwfmklqlwfg	2,81	0,32	lthyllwflgqlerkd	8,14	1,73
97	pigtdrhrplmkhrah	9,68	0,07	trhrlqwftgplrhfd	52,00	0,32	apqrwqwfagalrrfd	2,79	0,32	mpqwlqcfalwlcrnv	8,11	1,73
98	drhtplffsyteslag	9,64	0,07	apqrlqwflgplrrfk	51,16	0,31	tnhewkwfwgqlrqfy	2,56	0,29	tnqrllwfagqlrhfy	8,04	1,71
99	tnhrplfghrahdmts	9,62	0,07	tnqywkifsmaiqpea	51,12	0,31	antylqwfagparhfd	2,53	0,29	telyewkvylflcrwv	8,04	1,71
100	gtdrhrslfghrqped	9,56	0,07	apallqwfaytmhiqp	51,11	0,31	apqrwqwfagalrrfd	2,52	0,29	lnhylswfagkarkfy	8,04	1,71
101	tnhymhlrgcfmaiqp	9,53	0,07	drerlqwftlfdhnah	50,97	0,31	ltgrltcfegqlehfd	2,46	0,28	ecqylvwfagalrnfd	7,98	1,70
102	fikdnlycielnhcnq	9,37	0,07	tnhylqwkatparhdd	50,71	0,31	tnhylqwfagplwrfd	2,45	0,28	tnhyllwiycplrqfy	7,81	1,66
103	tnhmekifsgplrrfd	9,20	0,07	tnhylqwfagpaslag	50,22	0,31	ppmrlqwfwgqlhrfd	2,38	0,27	cshylswfagparkfl	7,79	1,66
104	tnhyelfnhrahdnts	9,15	0,07	dkprlewfagplrqfy	48,83	0,30	ftgisewfagplrrwv	2,33	0,26	ecqrlqwfagalrvfy	7,74	1,65
105	drgtdrhrphfghrah	9,05	0,07	apqrlqwfapalcrwv	48,48	0,30	ftgislwfagpaehfd	2,32	0,26	vgqktnffagllrrfd	7,73	1,65
106	mpvtlqnfkaplrrfd	8,96	0,06	wgkkynfyycpcylfq	48,21	0,29	apqwlqwflgqlerkd	2,29	0,26	tnqrlqwfvgalrafh	7,66	1,63
107	drhrplfghrarahdm	8,64	0,06	tnqrlqwfagplcrwv	48,15	0,29	lpqrwqwfagqlrrfd	2,27	0,26	asqrsnfiylflcrwv	7,60	1,62
108	drhrplfghrahdmts	8,56	0,06	hpqrlqwsklfphnah	48,14	0,29	apmrlqwfdgalrrfd	2,15	0,24	asqrllsfagqlerkd	7,58	1,61
109	fikdlqwmagpnhyfd	8,46	0,06	frgisdifsyleslwv	48,00	0,29	apqrwqwfagalrhfd	2,13	0,24	thhyaswfagparrfl	7,56	1,61
110	tnhyekifsyteslap	8,33	0,06	liqrlqwfalfdhnph	45,77	0,28	apqrwqwfvgqlrhfd	2,07	0,23	lttrlqwfagalrhly	7,46	1,59
111	apdrhrplfghrahtm	8,24	0,06	frlisqwfagplrrfd	45,76	0,28	ftgisewfagplrrfd	2,06	0,23	apqrtqwfagalrhfy	7,37	1,57
112	apqrlqpfagplrrag	8,20	0,06	tnhyewifsytmaiqp	45,39	0,28	cnhyltwfwgqlerkd	1,97	0,22	ncqrlqwfhgalrhfy	7,35	1,56
113	hrplfghrahdmtrfc	8,03	0,06	apqrlqwfagplrhfq	45,20	0,28	dhtylqwfagparhfd	1,91	0,22	snkkwnfiycplrhfy	7,33	1,56
114	tkhyekifsyteqpea	7,95	0,06	vskkwnfiycpcylmf	45,03	0,27	cnhyltwfwgqherfd	1,91	0,22	tpkkwnfiycplrqfy	7,33	1,56
115	apqrlqwfagteslsg	7,40	0,05	apcrlqwfagealrfd	44,02	0,27	tnhylqwfagancrwv	1,83	0,21	tnhycewfalflnrkt	7,25	1,54
116	plfghrahdmtcrrfd	7,15	0,05	ephylqwfagparhfd	43,24	0,26	wgkkwefiasnwrrfd	1,81	0,20	vgqkwnfiylflnrkt	7,24	1,54
117	tnhyekifsyteslag	7,06	0,05	tnhywdicsyleslkg	43,13	0,26	tqlyewifagpwtkhd	1,77	0,20	tmhklywfagllrrwv	7,16	1,52
118	tnhheaifsyterpfd	7,02	0,05	apqhnwaymalqlafg	41,85	0,26	anwylqwfagpwtkhd	1,61	0,18	lthylqwfagqlerkd	7,13	1,52
119	gtdrhrplfghhvmts	6,93	0,05	dhllrnsfcpallrwv	41,84	0,26	apqhnwlyagparhfd	1,55	0,18	ekqrlqwfalflcrqv	7,09	1,51
120	apqlfmaiqleallga	6,77	0,05	ligtlqwfycpcclmf	41,16	0,25	ppqrlqwfawqlerkd	1,46	0,17	asqrlqwfkgalrhfy	7,03	1,50
121	drhrplfghrahdmtv	6,70	0,05	drhrnqwfagahvlts	41,04	0,25	tnkywcwfwgslerkd	1,44	0,16	anhrlqwfycplrqfy	6,98	1,49
122	hrplsdliltdrhrpl	6,69	0,05	apqrlqwfaytnslad	41,03	0,25	apqrwqsfagplrqfm	1,43	0,16	lterltifagalcrwv	6,98	1,49
123	apdrhrplfghrahdm	6,49	0,05	lnsyewifsytmaiqp	40,87	0,25	ppqrlqdfwgqlrhfd	1,41	0,16	lltrltvfalflcrqv	6,94	1,48
124	drhrplfghrahdmtd	6,47	0,05	apqrlqwfaytnslal	40,82	0,25	apqwlqwfhgplrhfd	1,37	0,15	tsqrcewfagalrvfy	6,94	1,48
125	apqrekifsytnslag	6,35	0,05	lskhnwlwycpcylmf	40,72	0,25	apwwlqwfagvvtrwv	1,35	0,15	tttrltifagalcrwv	6,93	1,48
126	tnhyelfghrahdmts	6,27	0,05	tthyewifsytmaiea	40,40	0,25	epqrltwfwgqarhfd	1,32	0,15	asqrlqwfagalrrmd	6,79	1,45
127	drhrplfghyneslcg	6,21	0,04	hkprlewfagplrrfd	40,07	0,24	wklkwwfiycpcnqay	1,27	0,14	vgqkqnfiylflcrwv	6,66	1,42
128	tnhyekiiqyeallga	6,20	0,04	dhqrlqwflgplrrfd	39,98	0,24	apqhnwlymgalcrcv	1,23	0,14	tmhkllwfagslrkfl	6,59	1,40
129	apmhmnlrgcfmaiqp	6,19	0,04	epqyltwvwqqlerkv	38,19	0,23	apqwlqwfagplrrfd	1,20	0,14	tnhyllwfagqletkd	6,56	1,40
130	alfghrahdmtsatal	6,18	0,04	wgkyvnfiycpcylmd	38,03	0,23	tnhywkwswgqlerkv	1,11	0,13	apqkwnfiylflcrav	6,53	1,39
131	tnhyekifsytetsat	6,07	0,04	lrqrlqwfanpllrfk	37,34	0,23	apgrltcfwgqlerkd	1,10	0,12	tnqrllwfaypnrrfl	6,49	1,38
132	mhmnlrgfagplrrdd	6,06	0,04	apqrlqwfapalrrfd	36,80	0,22	elvrwswfwcqlnnah	1,10	0,12	tnhrlqwfaganrhfy	6,45	1,37

133	fikdnlthiadrhrpl	5,96	0,04	apqrlqwfqgalrrfd	36,52	0,22	cnhylqwlcwwmfwdn	1,06	0,12	ecqrlnwfklplcrwv	6,45	1,37
134	vnmhmnlrgcfmtsat	5,90	0,04	apllpnsfapallrwv	36,44	0,22	apqylqwfmgpwtkhp	1,02	0,12	pnhykswfagparkfl	6,44	1,37
135	hrplfghrahnmtyea	5,82	0,04	tnhylqlymalqlafg	35,51	0,22	apqrwkqfwgqlerkd	0,98	0,11	ahqkwqfiycplrqfy	6,34	1,35
136	apqrfghrahdmtsat	5,75	0,04	ldlayiweagalrrfd	35,39	0,22	ppqrlqwwcwwmrrfd	0,96	0,11	tgqkvnfiylflcrwv	6,25	1,33
137	apqrvpwghrahdmts	5,75	0,04	epqrlqwfagmlrrfd	35,34	0,22	apqrlqwfasalerkd	0,96	0,11	tnhrlqwfdgalcrwv	6,19	1,32
138	apnrlqwhahpdrwmq	5,48	0,04	tnhymqwfagkarhfd	33,95	0,21	avqwlqnlhdpllrfd	0,94	0,11	tnqrlqwfagalerkd	6,16	1,31
139	plfghrahagplrdfd	5,42	0,04	lintlqwflgpaylmf	33,80	0,21	tnhywkwewlfthnah	0,93	0,10	lttrlpifygalrrfd	6,14	1,31
140	gtdrhrplqghrahfd	5,26	0,04	plfglnfiycpcwlls	33,72	0,21	apqwlqwfwgparhfd	0,93	0,10	tnayllwfagqlenah	6,04	1,29
141	fikdplfghrahdmts	5,16	0,04	epqrmwlymalqlavg	32,84	0,20	epqrnwlymalqlwcg	0,90	0,10	epqrlqwfagparrfl	6,02	1,28
142	fikdnlthiqtmrrfd	5,10	0,04	apqrlqwfagleslag	31,98	0,20	apqwlqwfagpanhfd	0,88	0,10	ecqelqwfagalrqfy	6,01	1,28
143	ligtdrhrplflpafd	5,06	0,04	wgkklqnfagparhfd	31,96	0,19	epqrlswfagparefl	0,88	0,10	vgnkwnffagpnrrfl	5,91	1,26
144	apqdlqwfagprahdm	5,00	0,04	apllrnsfppallrwv	31,92	0,19	npqrlqwwcwalcrwv	0,88	0,10	asqrlqwfalflcewv	5,87	1,25
145	gtdyekifsyteslaq	4,94	0,04	tnhyldwfdgplrrfd	31,84	0,19	apyhwqwfasawrrfd	0,88	0,10	tvhyllwfavqlerkd	5,82	1,24
146	tnhtdrhrpefghrah	4,77	0,03	tnhylqwfaptnslag	31,44	0,19	ltgclpcfwgqlerkd	0,84	0,10	alhyyqwfsvpardnm	5,78	1,23
147	tnhyekifsydeslad	4,77	0,03	apqrlqwfaghkcrwv	31,04	0,19	apqrltcfwgqlerkd	0,82	0,09	asqrlqniylflcrwv	5,78	1,23
148	ppkempwyagpyrafd	4,76	0,03	frgyewdfsytmaiqp	30,55	0,19	tnhyaqwfagpwtkhd	0,81	0,09	ecqrlqwvagalcrwv	5,76	1,23
149	qlfghrahdmtsataa	4,40	0,03	tkprlewfatplrqmy	30,23	0,18	elvrwswfwmqlcrwv	0,80	0,09	apqrwnfimlflcrwv	5,73	1,22
150	plfgtrahdmtsaiqp	4,28	0,03	epqrlqwfldplrrfd	29,95	0,18	apqrlqwfagwwtkhd	0,79	0,09	ecqrlmwfagvcrhfy	5,61	1,19
151	ligtdrhrpgplrtfd	4,19	0,03	apaisdifsyleslak	29,03	0,18	anhywkwfwgqlerkd	0,77	0,09	aaqwlqcfalflcrqv	5,58	1,19
152	hrnkhglpphdmtqat	4,17	0,03	apqrcqwfagplrhfd	28,75	0,18	tnhynkwfwgqlerkd	0,76	0,09	aelyewifagalcqfy	5,50	1,17
153	plfdnlthiqtnhyea	4,07	0,03	hkprlwifsqvmaiqp	28,75	0,18	anpylqwfagpqlwfg	0,75	0,08	elqrllwfagqlerkd	5,44	1,16
154	fikdnlthiqtnhyea	4,06	0,03	hkmrlewfagpdrhfd	28,57	0,17	tnhywkwfwpqlerkd	0,74	0,08	vnhhllwfagqlcrwd	5,41	1,15
155	mpqrtqwsampytrfd	4,06	0,03	apqrlqwfagplrrfd	27,42	0,17	epqrltwfwdqlrhdd	0,71	0,08	tehyllhfagqlerkd	5,28	1,12
156	apqrplfghrasdmts	4,06	0,03	lllglqwfegplrrfd	27,27	0,17	apmylqwfwgqlerkd	0,68	0,08	asqrlqwfagpllqfy	5,26	1,12
157	ayprlqvfqdelrvfm	4,04	0,03	hkpslqwfagplrrfd	27,21	0,17	tnhyltwfwgqterkd	0,67	0,08	tnkkwnfiycplrqfq	5,11	1,09
158	apycdqafwgpdrrfd	4,02	0,03	apkrlqwkagplrhfq	27,04	0,16	lthywmwfwgqlerkd	0,66	0,07	apqwlqmfalflcrwe	5,08	1,08
159	crkyfhhtaldmdsat	3,80	0,03	drhrlqeftnplrrfl	26,95	0,16	tnwywkyfwgqwlawd	0,64	0,07	apqrlkwfagplrnfd	4,98	1,06
160	apllwnsfppalahdm	3,69	0,03	apllrnsfwgqlerkd	26,58	0,16	nqhywkwfwgqlerfd	0,64	0,07	vgqewnfinlfnrrql	4,97	1,06
161	gtechvplfqhrthpm	3,57	0,03	apqrlqwfagplrrfd	26,56	0,16	tnhywkwfwgqlerkd	0,64	0,07	tnqrlqwfavdyhnah	4,91	1,04
162	plfghrahdyteslag	3,53	0,03	ligtlwlymnqqlefg	26,04	0,16	epqdltwswgqlrhfd	0,59	0,07	anqklqwkagalrhfy	4,83	1,03
163	frgisdlielteslag	3,52	0,03	lskhewliycpcylmf	25,79	0,16	dpqwlqwfagplrrnd	0,58	0,07	ecqrlqvfagalrhfh	4,76	1,01
164	ligtdrthiqtnhsea	3,43	0,02	hkprsewfagplrnah	25,57	0,16	adhywkwfwgqlerkd	0,57	0,06	ecqsynlfahalrhft	4,71	1,00
165	drhrplfghvahdmea	3,37	0,02	apnrvqwfagparhfd	24,24	0,15	epqrwqwfagalrrpd	0,53	0,06	tthrlqwfagaarhch	4,67	0,99
166	plfghrahdmtsattl	3,23	0,02	epqklewhwgpleakd	23,89	0,15	apqrwewfagvltrwv	0,51	0,06	apqrlqfiycplrqfy	4,63	0,99
167	plfghrahdmtsayep	3,10	0,02	drhrlqwftgtnslsg	23,25	0,14	ppqrlqwwcwwmfwdh	0,51	0,06	ecqrlqwfycplrqly	4,62	0,98
168	gtdlhrplfgeallga	3,06	0,02	epqrltwyycpcylmf	23,20	0,14	tntylhwfwgqterkd	0,47	0,05	ecqrlqwfavfyhnap	4,55	0,97
169	dyhrplfghmtsatnl	2,97	0,02	qanrltafagahrrfn	22,75	0,14	vnkywkwnwgntersd	0,46	0,05	tnqrlqwfagplyqfy	4,54	0,97
170	plfghrahdmtsatal	2,85	0,02	cpqamtkfahalrrwd	22,09	0,13	ppqryqwwcgqlerkd	0,45	0,05	ppqrlqfiyqplrqfy	4,53	0,96
171	fhgisghrahdmtsat	2,81	0,02	hskhnwlysaplrrfd	21,67	0,13	tnqyatwfwgqlehfd	0,44	0,05	tnqrhqwfagalrrks	4,52	0,96
172	tagtnlqrptfcavah	2,75	0,02	ppqrlqwfayteslag	21,51	0,13	apqrlqwfwgqlerkd	0,44	0,05	apqkwafhycplrqfy	4,52	0,96
173	vnmhmnnrggplrpfd	2,67	0,02	epqvlqwftgpdrhfd	21,22	0,13	epqrltwfwsqlerkd	0,42	0,05	alqyllwfadqlerkd	4,50	0,96
174	hrplfgaiqpeallga	2,60	0,02	frprlewmagplrqfy	19,97	0,12	tnyywkwwcwwmfwdn	0,42	0,05	tnqrlqfiycplkqfy	4,42	0,94
175	plfgtrahdptsahdm	2,52	0,02	drhryikmclwmfwdn	19,94	0,12	apqrwqwfagalrdfd	0,40	0,04	tmhkllwfagnlerkd	4,37	0,93
176	prgysdligtdsqrsh	2,51	0,02	drhrlqwfthpdhnah	19,82	0,12	tnhywawfwvqwnrtd	0,39	0,04	tnhylpwfagplrhmt	4,33	0,92
177	fikdnlttiqtsytal	2,49	0,02	epqrlqwfagplrrfd	19,06	0,12	epqrltwfwgqlerkd	0,39	0,04	apqwlqcfhlflcrkd	4,23	0,90
178	pafghhahhrahdmts	2,40	0,02	apqrwkifsmapqpea	18,65	0,11	atqplqwfagparmfd	0,36	0,04	tnqrllwaagpnrrfl	4,13	0,88
179	klfehrahdmtsatal	2,32	0,02	tnhylqwnagparrfa	18,26	0,11	apqrvywwcwwmfwdn	0,36	0,04	tnqelqwfagparrfl	4,12	0,88
180	gtdrhcahmmtsathl	2,24	0,02	apqqlqwfagplrrfd	17,78	0,11	tnqrltwfwgqterkd	0,36	0,04	ltqrqqwpagalrhfy	3,99	0,85
181	ldgtdrfghrahdmts	2,20	0,02	epqrdtwflgplrrfd	16,37	0,10	tmqrltwfwgqnerkd	0,36	0,04	tnhrlnwfagparhch	3,96	0,84
182	gtdclrgcfmaiqpea	2,16	0,02	aellrnsfapalcrwn	16,31	0,10	tnhylswtwgqlerkd	0,35	0,04	ecqrlqkfqgalrhfy	3,87	0,82
183	qimdnltsiktdhyma	2,08	0,01	apqrlknfagparhfd	14,65	0,09	elvrwqwfasawvrfd	0,34	0,04	tqtrlqkfagklrhky	3,76	0,80
184	lpgtdrhnpyfgldaq	2,04	0,01	hkpelewfagplrhfq	14,28	0,09	wgpkanwfwgqlerkd	0,31	0,04	apqrlqkfalflcrqv	3,75	0,80
185	vnmhmlfghdahdmts	1,82	0,01	lpgtlqqflnperrfa	14,18	0,09	tnqrltwfwgymawdn	0,31	0,03	asqrlvqfagalrnfd	3,61	0,77
186	frgisdgafmaiqpea	1,75	0,01	lrgrlqtfagparhfd	14,15	0,09	apeyqqwfagalsafd	0,30	0,03	asqrlqcnalflcrwv	3,53	0,75
187	fiktnlthdmtlatal	1,66	0,01	arhrlqwqtnplrhfd	13,35	0,08	apqwwkwfwgqlerkd	0,30	0,03	tyqrlqwfagparhch	3,34	0,71
188	vnmhmnlrgcteslag	1,47	0,01	epqpltwiycpcylmf	12,23	0,07	tqlcewcfwgqlerkd	0,29	0,03	asqrlqwfagpardfl	3,29	0,70
189	plfghrapdmtsatad	1,40	0,01	apllresfagplrhfq	12,07	0,07	apqrwkwpwgqleskd	0,28	0,03	asqrlqwfagqaerkd	3,06	0,65
190	apqyekifsdteslag	1,39	0,01	apqrlqwyagahdlts	11,38	0,07	apqrwqwfagqterkd	0,26	0,03	qnhllwefagapmrwv	2,82	0,60
191	lhgtdrhrpmaiqpea	1,33	0,01	nkllknpfapallrtk	11,34	0,07	tnhyskwvwqqleqkd	0,25	0,03	tnhsvlwfsnqlprtd	2,76	0,59
192	arqcfmaiqeeallga	1,32	0,01	agkkwnyidcpcylmf	10,38	0,06	npqwlqwfagplerkd	0,25	0,03	ewqrlqwyamamkhvm	2,55	0,54
193	apqghrahdmtsatal	1,30	0,01	apqdlqwfagplrrfd	7,71	0,05	lpqrwqweagalrndk	0,25	0,03	teqrlhweaghlkhfy	2,49	0,53
194	apllrnsfppaeslag	1,28	0,01	apqrlqqfsyleslag	5,69	0,03	epqwlqwfagplrdfd	0,22	0,02	pphylpwfagparhch	2,46	0,52
195	qigtdrhrphdmtsat	1,24	0,01	adsrlqsfagalmrye	5,41	0,03	wdwrvtwfwgqlpcfd	0,18	0,02	tmhkllmfagelerkd	0,94	0,20
196	fikdnlthiqtnpyea	1,08	0,01	aphywkissmaiqpea	4,24	0,03	apeewmefagnerrfd	0,17	0,02	tnhylchfagqeerkw	0,74	0,16
197	plfghdahdmtsatal	0,98	0,01	dhqrlqdftgplrhfd	3,50	0,02	tvhtekwpwgqpewkp	0,14	0,02	tnvylcnfdgslerdl	0,46	0,10
198	gldrhrplegdeawkm	0,95	0,01	tnhylqtfagparwdn	2,78	0,02	ppqrlqwwcwwmeekd	0,13	0,01	snnrdevfagtsrhqy	0,45	0,10
199	ldgtdrhrpmtsatal	0,74	0,01	apqrlqkdagpdhnah	1,51	0,01	tnqrltwvwgqleskd	0,12	0,01	tnqhllpfagpanhfd	0,36	0,08
200	apqdlrahdmtsatal	0,62	0,00	drhrlqsealfdhsah	-0,17	0,00	ftgidewfagplrqnd	0,10	0,01	telyeqifagpancfd	0,33	0,07
	**Lead Peptides**	**x**	**c**	**Lead from Gen1**	**x**	**c**	**Lead from Gen2**	**x**	**c**	**Lead from Gen3**	**x**	**c**
	apqrlqwfagplrrfd	26,89	0,19				tnhywkwfwgqlerkd	0,73	0,08	tnhyllwfagqlerkd	11,00	2,34
	tnhyekifsyteslag	7,06	0,05				apqrwqwfagalrrfd	2,81	0,32	tnqrlqwfagalrhfy	9,72	2,07
	drhrplfghrahdmts	8,56	0,06				apqwlqwfagplrrfd	1,07	0,12	ecqrlqwfagalrhfy	9,22	1,96
	plfghrahdmtsatal	2,85	0,02				epqrltwfwgqlrhfd	4,08	0,46	asqrlqwfagalrhfy	10,45	2,23
	mhmnlrgcfmaiqpea	4,37	0,03				ltgrltcfwgqlerkd	3,33	0,38	vgqkwnfiylflcrwv	10,15	2,16
	vnmhmnlrgcfmaiqp	6,22	0,04	tnhylqwfagparhfd	107,48	0,66	tnhyllwfagparhfd	13,49	1,53	tnkkwnfiycplrqfy	7,08	1,51
	apllrnsfppallrwv	27,09	0,20	epqrltwfwgqlerkd	78,41	0,48	tnhylqwfagparhfd	3,27	0,37	lnhylswfagparkfl	13,33	2,84
	hrplfghrahdmtsat	6,29	0,05	apqrlqwfagalcrwv	113,79	0,69	anhylqwfagpwtkhd	1,92	0,22	tnhyllwfagpnrrfl	10,78	2,30
	gtdrhrplfghrahdm	9,14	0,07	apqrlqwfalfdhnah	72,93	0,44	tnhylswfagparrfl	10,19	1,15	telyewifagalrqfy	8,89	1,89
	ligtdrhrplfghrah	14,97	0,11	hkprlewfagplrqfy	68,31	0,42	elvrwswfwcqlnrkt	2,86	0,32	tmhkllwfagllrrfd	9,61	2,05
	frgisdligtdrhrpl	12,83	0,09	ligtlqwflgplrrfd	79,12	0,48	epqrlqwfalfthnah	7,12	0,81	ppqrlqwfvgalrhfh	8,97	1,91
	fikdnlthiqtnhyea	4,06	0,03	drhrlqwftgplrhfd	56,34	0,34	tnhylqwfagparhfh	5,67	0,64	apqrlqwfycplrqfy	6,88	1,47
				tnhyewifsytmaiqp	51,16	0,31	apqrlqwfagalcrwv	6,63	0,75	cthylvwfagalrnfd	11,36	2,42
				aplllqwfagplrrfd	54,72	0,33	ppqrlqwfagalrhfy	25,18	2,85	lttrltifagalcrwv	6,60	1,41
				apqrlqwfagplrhfq	46,35	0,28	apqhnwlymalqlwfg	3,44	0,39	tnhrlqwfavfyhnah	8,08	1,72
				dhqrlqwfanplrrfl	43,40	0,26	ftgisewfagplrqfy	5,57	0,63	apqwlqcfalflcrwv	7,48	1,59
				apkrlqwfagahdlts	43,52	0,27	cnhyltwfwgqterkd	0,95	0,11	thhylswfagparrfl	8,11	1,73
				lrgrlqwfagealrfd	57,64	0,35	dhqrlqwfagvltrwv	6,55	0,74	qnhylwifagalcrwv	13,34	2,84
				plfglqwfagplrrfd	52,76	0,32	wgkkwnfiycplrqfy	16,86	1,91	apqrlqwfalflcrqv	6,82	1,45
				frgisdifsyleslag	38,49	0,23	apqrlqwfalflcrwv	12,26	1,39	tnhrlnwfalflcrwv	8,33	1,77
				apqrlqwfaytnslag	48,87	0,30	tqlyewifagalcrwv	10,11	1,14	lthylqwfagparhch	7,36	1,57
				tnhywkifsmaiqpea	44,63	0,27	ppqrlqwwcwwmfwdn	0,23	0,03	apqrlqwfalflnrkt	5,80	1,24
							apqrlqwfasawrrfd	0,41	0,05	apqrlkwfagplrqfy	7,37	1,57

	Generation 5	x	c	Generation 6	x	c	Generation 7	x	c	Generation 8	x	c
1	tnkewnwfagalrhfy	35,06	6,03	pnkevhwwdgalrmwl	65,96	9,05	nwknwnwwagalrmfl	128,99	15,05	nwknwewwagalrmwl	73,49	20,01
2	vaskwqwwagalrkfl	33,28	5,72	tsqevqswagalrmwl	55,24	7,58	tnkdwnwwaqalrkfl	99,14	11,57	nwknwnwwagaltqfl	67,37	18,34
3	tnkkewifygalrqfs	31,54	5,42	tnkewnwfngalrmfl	53,52	7,34	tqkewqswagalrmwl	90,20	10,53	nwknwawwaqalkkfl	64,93	17,68
4	vgskewifygtlrqfy	29,21	5,02	tnkelnwwaqalrkfl	52,40	7,19	qnkevhwwdgalrmwl	76,97	8,98	dwknwnwwanalrmfn	64,06	17,44
5	wqwwltwfagalrhay	29,07	5,00	wkwwltwfagalrkfh	47,00	6,45	ymskwqwwaqalrmfl	69,13	8,07	nwknwnwvwgalrhfl	55,08	15,00
6	ttqwldwfagalrhfl	28,87	4,96	ttqwldwaagalrhws	43,61	5,98	pnkevhwwdgalrmwl	68,58	8,00	nwknwnwwagalrmfl	51,97	14,15
7	mqwwhwifagalrhfd	27,34	4,70	asqwlqwwagalrqfy	43,32	5,94	wqwwlnwfngalrmfl	68,23	7,96	nwknwnwwdgalrmwl	50,03	13,62
8	tnkvwnwvagalrcfy	27,08	4,66	vakkwqwwagllshfy	43,10	5,91	tnqrlqwwygllrqfl	66,86	7,80	nwknwlwwagqlsktl	48,08	13,09
9	tnnrlqwwagalrkfl	26,71	4,59	vmskwqwwagalrkfl	42,86	5,88	vaskwnwwagqlrkwl	66,80	7,79	nwknwywwaqalrmfl	47,99	13,07
10	tnkkewifygalrqfy	26,38	4,54	takvwnwwagalrkfl	42,32	5,81	tqkewnwfngalrhwl	66,09	7,71	wkktwwwwllalrhfl	47,72	12,99
11	lsqrlqwwagalrkfl	25,62	4,41	wqwwlmwfagalrhfl	42,29	5,80	wkwwlhwfdgalrkfl	64,29	7,50	wnsswnwwagqlrkwl	46,08	12,55
12	asmevqwwagalrmwl	25,43	4,37	wqwwlwwfvgalrkml	41,99	5,76	pnkevhwwdgalrmwl	63,77	7,44	tnkdwnwwaqalrkfl	44,32	12,07
13	asqdlqwwagalrhfy	24,67	4,24	thpevqwwagalrmwl	40,54	5,56	tsqevqswagalrmwl	62,93	7,34	qykkwqwwaglllhfl	41,90	11,41
14	tnhywlwwagalrkfl	24,41	4,20	vaskwqwwagalrkfl	39,61	5,43	qakkwqwwagllshfl	62,39	7,28	nwknwvwwygllrqfy	41,37	11,27
15	tnknwnwvagalrhfy	24,28	4,18	nwknwnwvvgalakfc	39,16	5,37	pnkkwqwwagwlshwy	61,00	7,12	pnkhwqwwagwlrmwl	41,25	11,23
16	tsqrlqwwagalrkfl	23,31	4,01	tnkewnwfagalrhfy	38,94	5,34	tnqetqswagalrmwl	60,36	7,04	tmsnwnwwakalrmfl	41,09	11,19
17	asprlqwwagalwhfh	23,10	3,97	tnkkewifygalrqfs	38,70	5,31	takvwnwwlgalrhfl	59,66	6,96	wqwwwnwaagalrmfv	40,58	11,05
18	tnnrlqwwagalrkfl	23,08	3,97	tnwnlwwfagalrhfy	36,44	5,00	nwknwnwvdtalakwl	58,29	6,80	dwwnwnwwagalrmal	38,87	10,58
19	tnwylwwfvgalrkfl	22,98	3,95	asmevqwwagalrmfl	35,75	4,90	tnkawqwwagalthfl	57,02	6,65	tnkdwnwwhqllshfv	38,69	10,54
20	tnykwnwfagalmhfy	22,95	3,95	tnqrlqwwygalrqfl	35,51	4,87	wqwwlmwfagalrhfl	55,63	6,49	pwknwnwwagalamsl	38,47	10,48
21	tnnalswfagalrhfy	22,95	3,95	vgskewifygtlrqfy	35,04	4,81	wqwwlwwfvgayrkmd	55,57	6,49	tvqkwqwwagllssfl	38,44	10,47
22	wnkqpnwfagalwhfh	22,90	3,94	wqywltwfadalrhfl	34,75	4,77	vmskwwwwagalrmfl	55,11	6,43	qakkwmwwagllshwl	37,49	10,21
23	tnhyslwfagalrhfm	22,89	3,94	tnkkewifygalrqms	34,65	4,75	hqywqtwfadalrqfy	53,68	6,26	wqwwlnwfngalrmfl	37,37	10,17
24	tnkkewifygalrkss	22,64	3,89	nsnrlqwwagalwhfh	34,60	4,75	tnkewnwfngalrmfl	53,57	6,25	nwenwnwwlqalrhfl	36,58	9,96
25	tnhyllwfagalrhfs	22,39	3,85	wnkkewifygalrqfs	34,41	4,72	wqwwlhwwaealrmwl	53,28	6,22	wqwwlwwfvaklrphl	36,27	9,87
26	tnkqwnwfagalrhfy	22,01	3,79	agskcwifygalwhfh	34,37	4,71	wqwwlwwfvaalrkfh	52,29	6,10	wkdwdhwfagwlshwy	36,12	9,83
27	lthalswfyqalrqfy	21,83	3,75	tnkewnwfegalrhfy	34,34	4,71	wkwwltwfasalrsfl	51,42	6,00	tnkdwnwwaaalrmwl	35,85	9,76
28	tnkknwifygalrqfy	21,81	3,75	tskkewifygalrqfs	34,04	4,67	akwwltwfagalrmwl	51,34	5,99	tnhawqwwaqaldhfl	35,42	9,64
29	tnhywlwfagqlrhfp	20,93	3,60	lsmkwwwwqgalrkfl	33,24	4,56	wqwwlewfvgalrkfs	50,83	5,93	nwmnynwwagalrmfl	35,37	9,63
30	tnhylwwfvgalrhfh	20,92	3,60	tnktewwaagalrkfl	33,09	4,54	wqewlhwfadalrhfl	50,66	5,91	wqwwlmwfagalrdfh	34,82	9,48
31	lnkkewifygalrqfy	20,69	3,56	wnkqenwfahwlrhfy	32,69	4,48	wqqwlqwwagalrqsy	50,59	5,90	nwkewnwfagalrmfl	34,37	9,36
32	tnhyllwfagalrhfy	20,62	3,55	tnkewnwfagalrhay	32,65	4,48	vashwqwwmgalrmwl	50,23	5,86	wqwwlhwwemalrmfl	34,01	9,26
33	vgskwwiaygalrqwy	20,20	3,47	vgsvwnwvagalrcfy	32,62	4,48	wqkvwmwwagawrkfv	49,55	5,78	tqswwqswagalamwl	33,50	9,12
34	tgsnwnffagalrhfy	20,15	3,47	tnsrwpwwagalrkfl	32,17	4,41	vhpevqqwagalrmwl	49,23	5,74	tnkdwdwwaqalrewl	33,40	9,09
35	tnqylqpfagalrkfl	20,12	3,46	tnkkewifygalrqfy	31,87	4,37	pkwwltwfagalrmwl	48,64	5,68	tqkewqswaeklrmwl	33,26	9,06
36	tnkylwwfvgalrhfh	19,98	3,44	wqqkewifygtlrqfy	31,62	4,34	wqwwldwwagalrkfl	48,38	5,65	wnwwlhwwkealrmwl	33,06	9,00
37	qnhylwifagalrhfs	19,93	3,43	tnpkwwwfygalrhfy	31,62	4,34	kqwwvqswagalrmwl	47,60	5,55	tnkvwnwwaqalrkfl	32,91	8,96
38	asqrlqwwagalwhfh	19,81	3,41	mqhwhwifagalwyfh	31,51	4,32	tqkelnwwaqalrkfl	46,69	5,45	nwenwqywagalamwl	32,08	8,74
39	tnkslwifagalrhfq	19,80	3,41	wnwylwwfvgalrhfy	31,41	4,31	wqwwlwwfvaalrkml	46,36	5,41	wqwwlwwfvglyqkmd	31,78	8,65
40	aqwwlqlfegalrhfy	19,69	3,39	vnkhwdwfagalthfy	31,09	4,26	tnkelnwwaqalrkfl	45,33	5,29	nwkawwwfadalrqfy	31,62	8,61
41	dnhrlqwfvgalwhfh	19,52	3,36	ssprlqwwagalwhfh	30,50	4,18	takvwnwwagalrcfl	44,28	5,17	wnwwlnwfngalrpfh	31,52	8,58
42	tnhywlwfagqlrhfy	19,28	3,32	tnkswnwvagalmhfy	30,48	4,18	vmskwqlwvgalakfc	42,81	5,00	pnkkwqwwawwlrhal	31,43	8,56
43	tnkkewifagalrqfy	19,21	3,30	wnkkwwifygalakds	30,44	4,18	vgskwqywagalrkfl	42,80	4,99	kmskwqwwaqalrkww	31,28	8,52
44	ynwylqwfagalrhfy	19,00	3,27	mqwvhwifawalrhfy	30,03	4,12	tnwnlwwfngalrhfl	42,79	4,99	pnkdwnwwaealrksl	31,22	8,50
45	qnhylweflgalrhfy	18,96	3,26	lnkewewvagalrhhy	29,84	4,09	tqwwlmwfagalrhfl	42,78	4,99	tqkmwqawagalrmwl	30,52	8,31
46	lnhalswfagalwhfy	18,92	3,25	tnwhlwwnagalrhty	29,57	4,06	wqwwlmwfagaqnkml	42,58	4,97	wkwwlhwfpgalrmfh	30,50	8,30
47	anhylwifagalrhfd	18,85	3,24	tmykwnwfawalshfm	29,29	4,02	tnkewnwfagalrqfy	42,39	4,95	tnkdwnkwyqalrhfl	30,42	8,28
48	aqwylwwfaqparhfy	18,55	3,19	wqwwltwfagclrhfy	29,03	3,98	cnkeaqwwapllshfy	42,38	4,95	ywknwnwwamalrmkl	30,40	8,28
49	tnqtlqwfagalrhfy	18,50	3,18	tnhywlwftgalrhfy	28,70	3,94	wkwwltqfpgalrkfl	41,65	4,86	nklnwnwfsgalrhwl	30,33	8,26
50	tnhywqwwagalrkps	18,48	3,18	cqwwhwifagalrhql	28,59	3,92	wqywltwfagalrhfy	41,14	4,80	wqwwlwwfvgayrmfl	30,29	8,25
51	lnqaldwfagalphfy	18,45	3,17	nakvwcwfagalrhfy	28,29	3,88	vmskwqwwagalsqfk	40,68	4,75	tqkewqswagalrmwl	29,79	8,11
52	tnwylwcfvgayrhfy	18,38	3,16	wqwwltwwagalyhfh	28,13	3,86	pnkewnwfagalrhfa	40,22	4,69	qmknwnwwagalrmfl	29,69	8,08
53	alqrwnwfagalrhfy	18,34	3,15	wwkqplwfagalwhfs	28,03	3,85	wkwwltwqamalrnws	40,00	4,67	nqwwlwwfvgayrkfv	29,69	8,08
54	asqrlqwwagalrkfl	18,00	3,10	askennwfadalrhfy	28,00	3,84	asqwlqwwagalshny	39,67	4,63	wqwwlhwwasalrmwl	29,40	8,00
55	vwqrlqwwagalrtfl	17,47	3,00	vgsdlqwwagalrhfm	27,79	3,81	wqdwlmwfakklrvfs	39,25	4,58	tnkdwnpwwqalrmwl	29,14	7,93
56	tnkkewwfalfycrfh	17,42	3,00	tnkmewtfygalrqfy	27,76	3,81	wkkvvnawdgalrkfl	38,76	4,52	vhskwwwwagalskfl	29,12	7,93
57	lthylqwfagalrafy	17,29	2,97	lsqrlqwwagalakel	27,55	3,78	tswwhtwfvgalrkfh	38,62	4,51	nwkqwnmwagalrtfl	28,95	7,88
58	tnkqwnwfagalrlfy	17,25	2,97	tnykwnwfakalmhfy	27,47	3,77	pnkevmwwdgalrhfl	38,17	4,45	pwknwvwwagqlrhfl	28,94	7,88
59	qnhylwifagalrnfy	17,17	2,95	dqwwnwifagalrqfy	27,39	3,76	ttqwlnwaagalrhwv	37,70	4,40	wqwwcmwfagalrmwl	28,82	7,85
60	asqrlqwwagalvkfl	17,10	2,94	ttqwldwfygallkss	27,37	3,75	kgskewifygtlrhfc	37,56	4,38	mnkdwnewyallrqfl	28,56	7,78
61	tnwvlwwfqgalrhfy	17,08	2,94	ttqwlywfagalrkfd	27,13	3,72	nskvlnwwaqalrkfl	37,12	4,33	qnkevhwwagwlshwy	28,55	7,77
62	tnkkldwfagalrhfy	16,87	2,90	tnnavswwagclrmwl	26,67	3,66	tnqymqwwagalrqfy	37,11	4,33	nwkewmwfngalrhwl	28,53	7,77
63	thkkewifagalrqfy	16,87	2,90	lsqrlqwwagalrdfd	26,66	3,66	tnwnlwwfasalrhfl	36,52	4,26	nwdnwnwwagalrmwl	28,31	7,71
64	tnskewsfagalrhfy	16,67	2,87	tnkkewifygelrqfy	26,66	3,66	vmskwqwwhkallmfl	35,50	4,14	tqkewaswagalrwwl	28,21	7,68
65	qnhylwwfagahrhfv	16,62	2,86	tnlalswwagallhfh	25,92	3,56	asqhlawwagalakfc	35,49	4,14	wqwwlhwwlqalrhfl	28,07	7,64
66	lnhylswfcgayrhfs	16,62	2,86	ttqwlawfagalrkfl	25,72	3,53	tnsneqwwagllshfy	35,27	4,12	wnkdwnwaagalrhfl	27,67	7,53
67	tnhylqwfagalrhfy	16,61	2,86	asprlqwwtgalrkfl	25,68	3,52	ekwwltwfagalpmwl	35,11	4,10	wqwwlcwwagalrmpl	27,64	7,53
68	lnhalswfagalrpfy	16,59	2,85	ntqwllwwdgalrkfh	25,65	3,52	twqwllwfagalrhfl	34,60	4,04	lwknmnwwagalrmfl	26,96	7,34
69	tnhylqwfagalckfl	16,58	2,85	vgskewifygllshfy	25,21	3,46	ptqwldwaagalrhws	34,17	3,99	nvknwnwvdtllshfl	26,29	7,16
70	tgskwnffagalrhfy	16,55	2,85	asqhlqkwagalrhwl	25,10	3,44	vakkeqnwagllshfl	33,69	3,93	tnkdwnswtqalshfl	25,97	7,07
71	asqrlqwwagalrhfy	16,40	2,82	wqwwlewfagalrcfl	25,08	3,44	tswewnwfagllrhft	33,66	3,93	wqcwlwwwaaalrmwl	25,58	6,97
72	tnhywlwfhlfycrfh	16,40	2,82	tnnalswfagaqrhfy	24,48	3,36	wqywswifygtlrqfk	32,98	3,85	nwknwnwwagasrqfl	25,00	6,81
73	cnwylwwfvgalrhfh	16,34	2,81	asprlqwwvgalrhfy	24,44	3,35	tsqeltwfagahrkfh	32,91	3,84	wqwwknsfagalrmwl	24,99	6,80
74	vwstlqwfagalrhfy	16,34	2,81	tnhywlwwagklmkfq	24,21	3,32	qnmkewifygalnqfs	32,65	3,81	tnwwlhwfygdlrkfl	23,61	6,43
75	tnyytvwfagalrkfl	16,28	2,80	tnhynlwfagqlehfy	24,13	3,31	takvpnwwagalrcfl	32,06	3,74	wqwwlwwwagllehfl	23,03	6,27
76	anhylswfcgalrhfl	16,27	2,80	tnkvwnwvagalrsfy	23,90	3,28	wqywltwwagyqwhfh	31,99	3,73	hssdwnewaqalrkfl	22,99	6,26
77	lneyllwfagalrhks	16,24	2,79	twhywlwwagalrhfm	23,75	3,26	pqkevhwwdyalrmws	31,48	3,67	vvskwnwwatalakwl	22,95	6,25
78	tnhylswfcgalrhfy	16,13	2,77	tkhywlwwaealrhay	23,64	3,24	pnkevhwwagalrhfl	31,39	3,66	nwknwnwwagadryfl	22,84	6,22
79	thhyltwfagalrhfy	16,08	2,77	wqwwltwdagalrhav	23,37	3,21	tqmevqwwagalrmtn	31,37	3,66	wqwhlwwfvaalrkfh	22,53	6,13
80	tnhrlqwfvgalwhfh	16,03	2,76	dtqdwnwfagalmhfy	23,04	3,16	tnwcwwiwygclrafs	31,06	3,62	yqwwwqwwaqalrmnd	22,23	6,05
81	vgpkwnffagalrlfd	16,02	2,75	tnkqtnwwagalrlfl	22,96	3,15	vakkewifsgalrqms	30,85	3,60	pnkkwqwwagalehal	22,23	6,05
82	tnqrldwfagalrhnt	15,88	2,73	vaskvqwwagalrkfl	22,83	3,13	tsnelqwwagalwhfp	30,68	3,58	qnwwlhhwaealrmwl	22,07	6,01
83	asyylvwfagalrnwd	15,73	2,71	vaakwqwfagalrhey	22,71	3,12	tsqevqswcgalrmwl	30,62	3,57	wqkewqswayalymwl	21,85	5,95
84	lnhylswfcgalrqfy	15,73	2,70	tnkvwnwfygalrqqq	22,66	3,11	tqwcnmwfagalrhfl	30,59	3,57	tqkewnwwngylrmwh	21,73	5,92
85	tnhylqwfagalrmfy	15,45	2,66	mqwwhwifavalavfs	22,56	3,10	tmmevcwwagalrmfl	29,74	3,47	takvdnwwqgalrhfl	21,71	5,91
86	tnerlnwfalfycrfh	15,44	2,66	lmqyltwfagalrhay	22,50	3,09	wnpevvwwagalrmwl	29,62	3,46	wkwwlhpwagalrmwl	21,70	5,91
87	tnqtywwfvghlwhfh	15,38	2,64	tnndlqwwagalrvfn	22,39	3,07	nwkecnwwaqklrkfl	29,53	3,45	wqwwlhwsagwcshwy	21,60	5,88
88	aqwwlqctagvlrhfy	15,35	2,64	tmyrlqwwagalrwfs	22,35	3,07	nsqnwnwvvgalakfc	29,02	3,39	tnwslnwfngalrmfl	20,97	5,71
89	adwwlqcfagalrhfy	15,16	2,61	tntallwfagalrhfd	22,34	3,07	tnkkwtwfagalrlfh	29,01	3,38	vmsywnwfngalrmfl	20,81	5,67
90	vgskwnffagalrhfy	15,13	2,60	tnkvwnwvacalrhfh	22,24	3,05	thpevtwfagalrtfh	28,49	3,32	vasywnwfmgalrmfl	20,80	5,66
91	tnqrldwfagalrhfy	15,11	2,60	tnhyalwwagalrkll	22,01	3,02	dyqwldwaagalrhwl	28,49	3,32	vmskwkwwagalrmfl	20,06	5,46
92	tnkkewifygavrhfy	15,11	2,60	tsqrdqwwapalrhfy	21,99	3,02	nsnrlvwwaglashfy	28,38	3,31	qakkwqdwagqlrkwl	19,59	5,33
93	lthylqnfagalrhfy	15,04	2,59	cgskewifagalrhfy	21,91	3,01	nssrlqwwcgalwhfh	28,23	3,29	tqkwlwwfqatlrkfh	18,87	5,14
94	tnhslqwfagalrhfy	15,02	2,58	mqwwhnwfagaledfy	21,87	3,00	tnkcwnsfngalrmwl	27,66	3,23	wlsktwwwagalrmfl	18,82	5,12

95	tnhylqwfagalrqfy	14,87	2,56	vaskewifygtsrqpy	21,78	2,99	wnwnlwdfegalrhfy	27,40	3,20	vaykwnwwagalrmfl	18,82	5,12
96	dsqrlqwwagalrhwh	14,72	2,53	tnhymllfagalwhfh	21,64	2,97	vpkelewwagalrhfl	27,17	3,17	tqhelhwwaealrmwl	18,67	5,08
97	tnyylvwfagvqrhfy	14,69	2,53	vnknwkwvagalrhfy	21,19	2,91	vgskewifygalvmfl	26,85	3,13	vaskwnwwagalrkwh	17,72	4,82
98	tnkkwnwvagylrhfy	14,25	2,45	tnykslwfagalrhfl	21,16	2,90	tnpevqwwsgalrtwl	26,83	3,13	tqkewnwfngalrqfl	17,62	4,80
99	vgskwnwfagqlrhfy	14,08	2,42	tnyvwnwfagalmhay	21,02	2,88	tqywlmwfavalrhfl	26,76	3,12	wqwwlpdfagalrhfk	17,44	4,75
100	lnhylswfcgalrqfy	13,80	2,37	tnyywlifygalrkss	20,88	2,86	awpevqwwagllshfy	26,69	3,11	tdavwnwwlgalrmfl	17,34	4,72
101	aqwylswkcgalrhfy	13,79	2,37	tnkkewifylalrhfd	20,80	2,85	ttkvwnwwagallqpy	26,61	3,11	vmykwwwwngalrefl	17,32	4,72
102	lehrlqwfvgalwhfh	13,70	2,36	tnkqwnwfkghlwhfl	20,47	2,81	tnkkewwfavalrhfy	26,48	3,09	wkwhlhwfdgalrkfl	17,13	4,66
103	yhhylkwfagalrqfy	13,65	2,35	tntylwwfvgalrvfl	20,42	2,80	wnpelwwfvgalrkml	26,33	3,07	tqkewqswalvlakwl	17,11	4,66
104	tnhylwwfvghlrhfh	13,61	2,34	takkewifcgalrqfs	20,38	2,80	asqwlqwaaqelwhfh	26,03	3,04	tnkawqkwagalrmfl	16,47	4,48
105	lnsylmwfsgparhfy	13,57	2,33	tnykwnwfagtlrhfp	20,25	2,78	asvwlqlwagalrmwl	25,75	3,01	qkwqlhwfdgalrkfl	16,36	4,45
106	ecskwnffagalrhfy	13,52	2,32	vaskwqwwagclrkfl	20,03	2,75	pnwevhwwlgalrmfs	25,71	3,00	ymskwqwwaaalrkeh	16,33	4,45
107	tqwwlpcfagalrhfy	13,44	2,31	veskhwifagalrsfm	19,79	2,72	tacvwnywagalrhfy	25,40	2,96	tnkdwnswegalrmwl	16,23	4,42
108	asqrlqwwagslqhfy	13,21	2,27	vykkwqnwagalrhfy	19,57	2,69	wqywltwqwdalrmwl	25,31	2,95	nwknvhwwdgslrmwl	15,84	4,31
109	tnskwnffagalrtfy	13,00	2,24	vgskewifygtlayfy	19,50	2,67	kkkewnwfagalrhfy	25,08	2,93	tnkdwnwwsqslrkfs	15,54	4,23
110	tnkqwnlfagalrsfy	12,99	2,23	tnhyslwfagtlvhfm	19,46	2,67	tnkkewiflgalrqfs	24,83	2,90	qahkyqwwngwlshwy	15,52	4,22
111	tthylqwfakalrhfy	12,98	2,23	wnhylywfagtlrhfs	19,32	2,65	nwkswpwtvgalrhws	24,12	2,81	tnkevhwwtaalrcwl	14,27	3,88
112	tnnelqwfvgalrqfy	12,78	2,20	tqyklqwwagalrhfy	19,21	2,64	tnkkewifdgalrswl	24,03	2,80	twkkwnwfngalrmfl	14,05	3,83
113	ldhaewiftgalrqfy	12,67	2,18	vadaskwfagalrhfm	19,13	2,62	thnwlwwfvtatrkml	23,89	2,79	vaskwnywagaltkfl	13,59	3,70
114	vgqqlqwfagtlrhfd	12,58	2,16	tewwldwfvwalrhel	19,13	2,62	tykkvwifygalqqfl	23,87	2,79	tnqrlqcwygllrqfl	13,54	3,69
115	tchylwifagalrhfd	12,53	2,15	tnwevqwwagalqmwl	19,03	2,61	wnkevswwdgalymwl	23,02	2,69	nwyevkswahalrmwl	13,33	3,63
116	asskwnfyagalrhfy	12,34	2,12	mtywhwifagalnhfd	18,91	2,59	wpqwldwnagalrhws	21,93	2,56	tsqewnwwdqalrkfa	13,22	3,60
117	tnmylwhfvgalrhfd	12,33	2,12	asadlqwwtgalrkft	18,58	2,55	asmevqwwagacrkfl	21,23	2,48	pskevhwwdgalrmwl	12,82	3,49
118	lnhywldfagqlrhfy	12,32	2,12	tnwwhwifagaeehfd	18,50	2,54	asmkwqwwagavrkfy	20,89	2,44	tsqelqwwagllshcl	12,67	3,45
119	tnhyltwfkgalrhfy	12,30	2,11	tnknwnwvkgalrhfq	18,20	2,50	nsnrlqwfagalrnfy	20,80	2,43	paskwnwwagqyrkwl	12,62	3,44
120	tnqtlqwfacalrhfy	12,27	2,11	tnkqwnwfayalrhay	18,19	2,49	vgskewifdgalwmwl	19,34	2,26	takkwnwwhgalrkfl	12,43	3,38
121	tnhylwifagalrhdd	12,24	2,11	asmevqwwagalrmyd	18,05	2,48	nskevcwwdgalrmkl	19,12	2,23	cnkavdwfvgayrkmd	12,35	3,36
122	eqwwlqcaagalrhfy	12,06	2,07	vgskewifwgplrhfl	17,90	2,46	wpwwpwwfqgalrkml	18,96	2,21	tqtwlwwnvaalrkwy	12,30	3,35
123	vqqqlqwfagalrhfy	11,83	2,03	wqwwltmfygthrqfy	17,79	2,44	tvqnvqswagalrmvl	18,77	2,19	tykdwnpwadalrmwl	12,22	3,33
124	ynnrlqwfvgalrlfy	11,79	2,03	mqqhhwifagalrhfy	17,26	2,37	asqnldwfadalrhfy	18,69	2,18	hqyawqwwagamshfl	12,10	3,29
125	tnnrlqwfagalrhfy	11,78	2,03	vashwqwwvgasrkfl	17,05	2,34	tnqkewifygtlvqfy	18,69	2,18	qnkevhwwdgalrmwl	11,97	3,26
126	asqrlqwfagalrnfy	11,77	2,02	atqmldwfagalrhfl	16,44	2,25	vmskwqwwcganrqfs	18,58	2,17	tnkawqwwegalrmml	11,74	3,20
127	lqwwlqckagallhfy	11,71	2,01	asqnlqwfygalrlfy	16,39	2,25	tnkewqwcagalrhfl	18,42	2,15	hsywqtyfhdalrmwl	11,47	3,12
128	tnkkwnwvasalrhyy	11,66	2,01	mqkvwnwvagaarcfy	16,35	2,24	psqwldwaagplrhws	18,12	2,11	hnkevhwwdgalrmwl	11,18	3,04
129	tnhydlwfagaqrhfy	11,62	2,00	tnhyslwfagplrhnk	16,34	2,24	npknwnwvvgalslfl	17,83	2,08	tnkewewfmgalrkfl	10,99	2,99
130	tnhyllwfapalrhfs	11,46	1,97	tnkqwnwfagacrhfy	16,08	2,21	tnqrqkwwygalrqfm	17,76	2,07	ymskwqwwaqtvrmfl	10,85	2,95
131	tnhylqwflgelrhfh	11,45	1,97	tnhrlyqwagalrkfl	15,97	2,19	hsqwlqwwtgasrmml	17,54	2,05	nwkyenwvdtalakwl	10,78	2,93
132	tnkketwfagalrhfy	11,43	1,97	lnwypwwfagalrmay	15,82	2,17	stqwldifygalrqms	17,26	2,01	tnsdlqwwawalhksl	10,71	2,92
133	vgqwlqcfagtlrhfy	11,17	1,92	tnwytdwfagacmhfl	15,65	2,15	pnkyvcwwslalrmne	17,16	2,00	wqwewhwfngaerhwl	10,70	2,91
134	qnhylyifagalrhfh	10,94	1,88	aakkmwifygaercfs	15,41	2,11	vakkwqewdgalrvwl	17,11	2,00	tqkewnwfnqqlrkfl	10,55	2,87
135	thhrlhwfalfycrfh	10,91	1,88	tqwnltlfagalrhay	15,37	2,11	tsqevqswygalrqws	17,04	1,99	nwknwnwmagalkhfl	10,53	2,87
136	thhyltwaagalrqfy	10,79	1,86	lsqrldwfagalrhfn	15,31	2,10	tnhkewiaygalrhfy	16,81	1,96	tqknwnwwayakrvfl	10,42	2,84
137	tnqeldwfagalrhfy	10,75	1,85	wnkqpdwtagalrhfl	15,26	2,09	vakkwqwwsgltrqfy	16,78	1,96	thqetqtwagalrmwl	9,66	2,63
138	tnhrlkwfalfycqfl	10,70	1,84	tnnrlqkwagalrkfy	15,12	2,07	ttkellwwaqalcdfl	16,42	1,92	tsqlhqwwagalrmfm	9,27	2,52
139	wnqmlqwvagalrhfy	10,69	1,84	tnhyslwfpmalrhfl	14,73	2,02	pnkevhwwagalskel	16,39	1,91	takvmnwfngalrhwl	9,26	2,52
140	tnkkewivygalrhyy	10,53	1,81	tnykwnwfagaqrwmy	14,73	2,02	wqwkewifygnprqfs	16,14	1,88	tnqrkqwwagalrmfl	9,20	2,50
141	asqrlqwwagalphfy	10,39	1,79	tnhyllwfagacwdfq	14,70	2,02	qsqewanckgvlrnwl	15,98	1,86	aqkewqwwagaldhql	9,13	2,48
142	tnqhlqwfasllshkh	10,37	1,78	tnmewwifygtlsqfy	14,52	1,99	vmvnwmavvgalakfc	15,95	1,86	wlwplnwfngelrmfl	8,90	2,42
143	tnhylqwfagklrqfy	10,31	1,77	asmewnwfagaqrhfy	14,46	1,98	thpevqwlagalrqfl	15,27	1,78	qtkkwnwwaqalrvfp	8,50	2,31
144	tnhyslwfagaqrpfy	10,07	1,73	kqwwhwicagtlrqfy	14,36	1,97	asywlqwmagalrkfl	15,22	1,78	ymskvvswagalrmwl	8,40	2,29
145	ecqrtqwfagalrhfy	10,00	1,72	tnkkewiwagalrkfl	14,15	1,94	vashwqwhygalyqfl	15,12	1,76	tqkewnwvdgalrmwl	8,07	2,20
146	vgqqlqwfagalrhfk	9,80	1,69	vaskwvwcagwlrknm	14,08	1,93	tnqrtqwfagalrkeh	14,58	1,70	tnqetqswagalrmwl	7,94	2,16
147	tynrlqwfvgalkhfy	9,76	1,68	ttqwldwfalalrvfs	13,82	1,90	nwlhwnnvvgaldkhd	14,49	1,69	taqnwnpwagalrmfl	7,92	2,16
148	astrlqwwaghlrhfy	9,60	1,65	tkkkewifygalrkfl	13,73	1,88	asmevqwwaganrmwl	14,39	1,68	ymskwqwvaqalrmfn	7,80	2,12
149	tnkqslwfagaqthfy	9,52	1,64	asprlqwwagalrhmy	13,62	1,87	vmskhtwwdgalrkfh	13,99	1,63	qnkevhwwdgtlrmwl	7,62	2,07
150	nshylmwfatparhfy	9,32	1,60	tnknwnwvagalrvfl	13,41	1,84	tnkellawaqalrhty	13,82	1,61	qnhevhwwdgalrhwy	7,61	2,07
151	vgqrtkwfagalrqfy	9,31	1,60	tnkeewifygalrefy	13,25	1,82	lakvwnwkaglashfy	13,52	1,58	wpwclewfdgalrmfl	7,52	2,05
152	vgqnwnffagalnhey	8,95	1,54	tnkkewwwagaqrswl	13,20	1,81	thpevdwyygalrqfl	13,49	1,57	hqkewnwfngnlrmwl	7,48	2,04
153	tnkklqwfagalrhfy	8,90	1,53	tnkaewifygqlrkfl	13,10	1,80	lknrlqwwngalrmhl	13,46	1,57	mnkethwwdgalhhfl	7,36	2,00
154	tnkkewwvadalhhfy	8,76	1,51	tnyylwqfvgalrkfl	12,92	1,77	vakkwqwkagllssfy	13,33	1,56	nwknwnwvvnaprkmd	7,17	1,95
155	asqrlqifygalrqfy	8,66	1,49	tnnaysafagalrhfy	12,53	1,72	tnprlqwwmgalrqsl	12,42	1,45	vsskknwwagalrqfy	7,02	1,91
156	avewlqcfagalrhfy	8,60	1,48	tnkkwwwfkgklmhfy	12,46	1,71	tsqevqswagalrmwn	12,15	1,42	pnkwlhwdvgalrkfh	6,85	1,86
157	cqkkwnwvagalrhfh	8,52	1,46	tnnrlqwcagvlwkfl	12,16	1,67	vasqvhwvdgalrmwl	12,14	1,42	pnkevhwwlgatrkwl	6,83	1,86
158	lnhylswdagalrhfy	8,23	1,41	tnknwnwvaganrlfs	11,73	1,61	pnkevtwfadalrnfh	12,04	1,40	ymskwnwwaqaqrkfl	6,43	1,75
159	tnnsldwfvgalrnfd	8,18	1,41	tnkkenwfagalehfh	11,69	1,60	ynkewnwfagnleqfy	12,01	1,40	pnqrlqwwyglcrqtd	6,43	1,75
160	tnkketifygalrqfy	8,14	1,40	tnkvllwfmgalrhfs	11,46	1,57	tkwvlqswhgalrmfh	11,65	1,36	qakewnwfngmlrmfl	6,21	1,69
161	tnhylmwfagparcfy	8,10	1,39	tnkeeqwwavacrmwl	11,40	1,56	tnkkehifygalrmwl	11,40	1,33	qnkevhwwhgawtmfl	5,83	1,59
162	qnhplwifagtqrhfy	7,96	1,37	tnkrlhwwagakwhfh	11,35	1,56	vmskwqwcagalrkhh	11,24	1,31	vaskwnwdagqlkkwl	5,74	1,56
163	asqrlnyfagalrhfy	7,90	1,36	kdkkewipygalrkfl	11,34	1,56	vakkaqwpagllshfl	10,98	1,28	hqywqtewagalqmfl	5,70	1,55
164	tnhrldyfcgalrhfy	7,85	1,35	tnpewnwfagmlmhfy	11,10	1,52	thpevqkwavalrkfl	10,50	1,23	vseevqmwaqalrkfl	5,61	1,53
165	tnhhlswfcgqlrhfy	7,84	1,35	tnkewnwfagalrktk	10,96	1,50	pnkevnwwagakrmwt	10,29	1,20	pnkeqnwfngalrmfl	5,45	1,48
166	tnqtlqwtagalrhfd	7,18	1,23	tnkvmwitygalrhfl	10,13	1,39	tnmednwfygalrqfs	9,95	1,16	hqycqtwfasalrqfy	5,26	1,43
167	lnhylmyfagparnfd	7,03	1,21	tnnalkqfagalrcfy	9,42	1,29	tnkelpwfnghlrmfl	9,52	1,11	tnqeteswagalrmwl	4,72	1,28
168	ltqrldwfagamrhfy	7,00	1,20	asqrlqwwagamrkcl	8,90	1,22	tnketnwcagalrkfl	9,09	1,06	qnkevlwwdgalrmfl	4,70	1,28
169	tnkketifagalrqfy	6,90	1,19	tnkyewwcagacrkfl	8,54	1,17	tnkelnwyagavakfc	8,09	0,94	teeswpwwavalkkel	4,57	1,24
170	anhrlqwhvgalwhfh	6,76	1,16	aamekqwwagalrkss	8,26	1,13	tpknwnhvvgalakfc	7,41	0,86	nwkncswwhghlqmal	4,51	1,23
171	aaqrllwaagslrkfl	6,71	1,15	tsqrlwwfveanrkfl	7,96	1,09	pnkevhwmdgalykfl	7,35	0,86	nwkrlqwkygllrqfl	3,69	1,00
172	knhranhfasfycrfw	6,63	1,14	vaskwqmfygtlrqfy	7,90	1,08	asmeeqwwagahrvfl	7,34	0,86	qnkvvhwyagmlrmwl	3,25	0,88
173	asqpcqwwayslrksl	6,25	1,07	lsqrlqwwagplrhqy	7,35	1,01	pnkehhwcagalrkfl	6,82	0,80	takvwnphlgalrhfl	2,57	0,70
174	lqnylqcwaghvrhpm	5,87	1,01	hnhyslefagalrkfl	7,30	1,00	ttqweddaagwlnhwk	6,28	0,73	nvknwnwnagalrhkl	2,52	0,69
175	tnhrlnwfalkycrfy	5,73	0,99	anhywycwsgaqrtfl	6,86	0,94	wnkkedifymtlrqfy	6,11	0,71	tnkewnwpaqalrmfl	2,48	0,67
176	thhrlnwfakpycrfh	5,66	0,97	tnkkepifygalrqfl	6,75	0,93	tnkewnwytgalrmpy	5,94	0,69	tqkewmwfngaprhwl	2,06	0,56
177	tnkkewifagaqrhfy	5,58	0,96	wnkqpnwfagvnrkfl	6,61	0,91	tnskeeifygtlrqfy	5,42	0,63	tnqrthwsaqalrmfl	2,02	0,55
178	tcnrlqifdgalrqfy	5,53	0,95	tlkkepifygalrqfy	6,39	0,88	snkewdefngalrhws	5,28	0,62	tnkawqslagaylkmd	1,97	0,54
179	dlktekifdgalrqfy	5,50	0,95	tsqrwqsmagalrkfl	6,19	0,85	tnkkewikygalrmfl	5,21	0,61	mnkevtwvdgalrmwl	1,90	0,52
180	tnhylswpcgalrhfs	5,15	0,89	tnhyslweagalrhfy	6,19	0,85	ynkhsnwfngalawdd	5,17	0,60	tnkdvnwwyqahrmsl	1,84	0,50
181	tnhyltwmhgalrhfy	5,12	0,88	tnkrlqwwagaqrkfl	6,18	0,85	tnvenhwndgalrmwl	5,13	0,60	hhywqthvdtalakwl	1,53	0,42
182	tnkkwnwpagalrhfd	5,04	0,87	tnktwnwvagamrqfy	6,02	0,83	pnkevhcwdgalwhfh	5,13	0,60	pnkkwqswagalvmep	1,52	0,41
183	tnnyewieygalrqfy	4,88	0,84	tnnrnqwwagahrhfe	5,34	0,73	asmevqwwagdlrmtl	4,63	0,54	tnqrlqswagalrmvn	1,52	0,41
184	ecqrtqcfagalrhfy	4,86	0,84	eskvwnwhagalrcdy	4,73	0,65	tnkellyqagalrhws	3,80	0,44	pnkkmqwwagaqrmfl	1,43	0,39
185	tnnrlqwcagalrhfs	4,83	0,83	tnnrlqifygamrwss	4,65	0,64	vaskwqyyngalrmfq	3,72	0,43	tykevhwndgalrtwl	0,77	0,21
186	tnhrlqcfsqalrhfy	4,56	0,78	tnhylqqwahacrkfl	4,54	0,62	tnqkewifyealrtms	3,60	0,42	tnqrlnwqadayrqfy	0,56	0,15
187	qdaylwysawedrhld	4,48	0,77	tnkennsfagqlrqfy	4,05	0,56	vnkkewpfygklrqms	3,42	0,40	qnkevhwkpgalrmql	0,52	0,14
188	lnhrtqefagalrqfy	4,23	0,73	wnkqpmifygalrqts	4,01	0,55	tnkelnmwpqalrkal	3,40	0,40	qnkevvcfngalrmfl	0,43	0,12
189	enwylcefvmalqhfh	4,05	0,70	tnyalsafagaprhfy	3,75	0,51	ttqwldwnataerhfy	3,27	0,38	tqketqscagalrvwl	0,37	0,10
190	tnhyslwnamaqrhfy	3,80	0,65	tlkkeaifygklrqfs	3,64	0,50	ankcvhwtdgalqewl	3,15	0,37	mqkdwqqckgacrmwl	0,36	0,10

191	tnhrlmqfagparhfy	3,49	0,60	tnckpwidygylrkmv	3,38	0,46	tnqrlqwwagqdtkfl	2,55	0,30	nckaknwwagdermfe	0,24	0,06
192	tnkkemifagslrqfy	3,47	0,60	tnkkewcdygalrqfh	3,16	0,43	pnkeehwwdgahrmfl	1,78	0,21	vasklnwpngaqrmfl	0,24	0,06
193	tnyelvimygalrqdy	2,98	0,51	tsqrlqwmaganrqfy	1,95	0,27	tsqevqscagalrkfc	1,44	0,17	tqkecqschdalrmwl	0,22	0,06
194	tnhyltwpagaqrhfs	2,87	0,49	asqdlnycagalrcfy	1,56	0,21	plkcvhwcdgwvdmtl	0,94	0,11	tqqevqspaqalrawl	0,09	0,02
195	tnkkeqwfagqlrhey	2,68	0,46	tnktwnwnagadrqfs	1,29	0,18	wnkkeqifygalpqms	0,90	0,11	tnqetqswagacrmfl	0,07	0,02
196	tnsrlmwkatelrany	2,32	0,40	asqnwnqvanaqrhfy	1,06	0,15	tspecywkagatrmnl	0,87	0,10	tnkevewwdgaermwl	0,03	0,01
197	nnktensfalalrhew	2,22	0,38	vaskeqiwaganrqly	0,89	0,12	vaskwqwpagadrmwm	0,82	0,10	vaekwemtagqlpewy	0,02	0,00
198	qnhsqwikhkalskfd	1,70	0,29	tnnascwpagalrpfm	0,47	0,06	pnkelndwaqaspkfl	0,24	0,03	tdqevqswagalpmwl	0,00	0,00
199	tnqtlqhfagyhrtqy	1,62	0,28	tnkepnefagalrhfd	0,31	0,04	tnqelnqsanahqksl	0,07	0,01	tqktdqcdagalrmvl	-0,05	-0,01
200	tcskeeifagahrmfy	1,27	0,22	vgskeqifdgaerkfl	-0,03	0,00	wnketnwtataltkel	0,02	0,00	tnqeeqseagalrmfl	-0,06	-0,01
	**Lead from Gen4**	**x**	**c**	**Lead from Gen5**	**x**	**c**	**Lead from Gen6**	**x**	**c**	**Lead from Gen7**	**x**	**c**
	asqrlqwwagalrkfl	27,47	4,72	tnkewnwfagalrhfy	37,47	5,14	pnkevhwwdgalrmwl	86,14	10,05	nwknwnwwagalrmfl	62,44	17,00
	tnkkewifygalrqfy	21,19	3,64	vaskwqwwagalrkfl	44,27	6,07	tsqevqswagalrmwl	51,98	6,07	tnkdwnwwaqalrkfl	46,61	12,69
	tnkqwnwfagalrhfy	23,44	4,03	tnkkewifygalrqfs	32,73	4,49	tnkewnwfngalrmfl	44,25	5,16	tqkewqswagalrmwl	21,31	5,80
	tnqrldwfagalrhfy	17,89	3,08	vgskewifygtlrqfy	33,15	4,55	tnkelnwwaqalrkfl	43,98	5,13	qnkevhwwdgalrmwl	13,07	3,56
	tnnrlqwfvgalwhfh	20,44	3,52	wqwwltwfagalrhay	35,65	4,89	wkwwltwfagalrkfh	60,50	7,06	ymskwqwwaqalrmfl	30,27	8,24
	qnhylwifagalrhfd	16,97	2,92	ttqwldwfagalrhfl	38,43	5,27	ttqwldwaagalrhws	47,17	5,50	pnkevhwwdgalrmwl	11,36	3,09
	tnwylwwfvgalrhfh	18,63	3,20	mqwwhwifagalrhfd	31,20	4,28	asqwlqwwagalrqfy	52,44	6,12	wqwwlnwfngalrmfl	34,25	9,33
	aqwwlqcfagalrhfy	16,34	2,81	tnkvwnwvagalrcfy	33,96	4,66	vakkwqwwagllshfy	58,64	6,84	tnqrlqwwygllrqfl	24,23	6,60
	vgskwnffagalrhfy	13,56	2,33	tnnrlqwwagalrkfl	33,90	4,65	vmskwqwwagalrkfl	57,15	6,67	vaskwnwwagqlrkwl	26,27	7,15
	thhyltwfagalrhfy	16,03	2,76	tnkkewifygalrqfy	30,14	4,14	takvwnwwagalrkfl	59,38	6,93	tqkewnwfngalrhwl	22,53	6,13
	tnhywlwfagqlrhfy	20,60	3,54	lsqrlqwwagalrkfl	32,75	4,49	wqwwlmwfagalrhfl	47,95	5,60	wkwwlhwfdgalrkfl	35,26	9,60
	tnhyllwfagalrhfs	21,69	3,73	asmevqwwagalrmwl	30,80	4,23	wqwwlwwfvgalrkml	41,86	4,89	tsqevqswagalrmwl	9,33	2,54
	tnhylqwfagalrhfy	16,24	2,79	asqdlqwwagalrhfy	37,16	5,10	thpevqwwagalrmwl	44,70	5,22	qakkwqwwagllshfl	34,58	9,42
	tnqtlqwfagalrhfy	16,21	2,79	tnhywlwwagalrkfl	30,23	4,15	vaskwqwwagalrkfl	53,02	6,19	pnkkwqwwagwlshwy	37,60	10,24
	lnhalswfagalrhfy	18,70	3,22	tnknwnwvagalrhfy	26,53	3,64	nwknwnwvvgalakfc	40,21	4,69	tnqetqswagalrmwl	8,09	2,20
	lnhylswfcgalrhfy	17,24	2,97	tsqrlqwwagalrkfl	28,60	3,92	tnkewnwfagalrhfy	41,73	4,87	takvwnwwlgalrhfl	34,72	9,45
	tnhrlnwfalfycrfh	14,39	2,48	asprlqwwagalwhfh	28,29	3,88	tnkkewifygalrqfs	38,29	4,47	nwknwnwvdtalakwl	34,40	9,37
	tnkkwnwvagalrhfy	16,20	2,79	tnwylwwfvgalrkfl	24,32	3,34	tnwnlwwfagalrhfy	39,52	4,61	tnkawqwwagalthfl	42,14	11,47
	tnkkewifagalrqfy	18,92	3,25	tnykwnwfagalmhfy	25,81	3,54	asmevqwwagalrmfl	35,59	4,15	wqwwlmwfagalrhfl	33,21	9,04
	tnyylvwfagalrnfd	17,02	2,93	tnnalswfagalrhfy	31,84	4,37	tnqrlqwwygalrqfl	52,53	6,13	wqwwlwwfvgayrkmd	33,18	9,03
	ecqrtqwfagalrqfy	12,09	2,08	wnkqpnwfagalwhfh	35,41	4,86	vgskewifygtlrqfy	48,51	5,66	vmskwwwwagalrmfl	28,84	7,85
	lnhylmwfagparhfy	12,45	2,14	tnhyslwfagalrhfm	33,69	4,62	wqywltwfadalrhfl	42,50	4,96	hqywqtwfadalrqfy	20,48	5,58
	lthylqwfagalrhfy	15,15	2,61	tnkkewifygalrkss	31,18	4,28	tnkkewifygalrqms	49,05	5,72	tnkewnwfngalrmfl	17,71	4,82

	Generation 9	x	c	Generation 10	x
1	dwknwnwwanalrmfn	97,47	27,26	dwknwawwaqalknfl	47,705
2	dwktwnwwaqalkkfl	93,96	26,28	dwknwnwwygalkkfl	34,395
3	nwknwewwagalrmwl	88,94	24,88	dwknwnwwanalrmfl	32,7
4	dwknwnwwanalrmcd	85,17	23,82	dwktwnwwaqalkkfl	31,415
5	dwknwnwwanalrvfn	84,82	23,72	dwknwawwaqalkvfl	30,365
6	nwknwewwagalrkfl	83,93	23,48	dwknwnwwanalrmfn	29,75
7	nwknwawwaqalkkfl	79,77	22,31	dwkqwnwwlgqlrkwl	28,21
8	nwknwnwwlgqlrkwl	79,46	22,22	dwknwsvwlgqlrkwl	27,005
9	nwknwawwaealrmfl	78,95	22,08	dpknwswwlgqlpkwl	26,995
10	dwknwnwwagqlrhwl	73,12	20,45	dwkewswwlqalrkwl	26,845
11	nwknwewwagylrmwl	70,37	19,68	dwktwnwwagalckfl	25,92
12	nwknwnwwaklllhfl	67,80	18,96	dwknwnwwagqlrmwl	24,96
13	nwknwnwwagalrmfl	67,30	18,82	awmnwnwwaqalrkfl	24,56
14	dwknwswwlqalrhfl	66,48	18,59	dwknwevwasalrmwl	24,42
15	nwknwawwaqalckfn	66,21	18,52	ewknwnwwsgalkkfl	23,725
16	nwknwnwwagalkkfl	64,87	18,14	nwknwaswaqaltqhl	23,71
17	wkkdwnwwaqllshdv	64,64	18,08	dwknwnwwanalrmcd	23,64
18	nwknwnwwanaltqhl	63,79	17,84	eqknwewwaqalkkfl	23,615
19	awmnwnwwaqalrkfl	60,02	16,79	dwknwewwagylmmwl	23,08
20	nwknwnwwaqelrmfl	58,85	16,46	dwktwnwwaqalkkca	22,535
21	nwknwswwaqalrmfl	58,21	16,28	dwknwnwwanalrvfn	21,73
22	nwknwnwwagaltqfl	55,20	15,44	dwkhwnwwagalrmwl	21,635
23	nwenwawwqqalkkfl	55,15	15,42	dwknwqwwalalrmfl	21,285
24	wqknwewwagalrmll	53,09	14,85	dwknwntwanalrmcd	21,105
25	dwknwnwwaglllhfl	52,71	14,74	dwhnwnwwamaltqha	20,945
26	lwknwnwwaqalkkfl	52,51	14,69	dwknlewwacalkkfl	20,895
27	wnkdwnwwlqllchvv	51,79	14,48	nwknwewwagklrmwl	20,79
28	hwknwnwwanalrmfl	51,16	14,31	awdntnwwaqylrkfl	20,36
29	nwknwnwwdqalrhfl	50,58	14,15	nwknwnwwlqalkkfn	20,355
30	pwknwnwwagllstwl	49,27	13,78	dwkswnwwlgqlrqwl	20,345
31	dwanwnwwanalrmkl	47,76	13,36	dwknwnwwanalsvdc	20,17
32	nwddwnwwaqalrkfl	44,60	12,47	nwvnwnnwanaltkwl	20,095
33	vwknwnwwenalrefl	44,14	12,35	dwktwnwwaqaakkfd	19,81
34	nwknwlwwagqlrdwl	43,51	12,17	vwkawewwkgylrmwl	19,465
35	nwknwawwtqalkkfe	43,09	12,05	nwknwnwwanllrmcd	19,225
36	dwktwnwwasalrmfn	42,92	12,00	mwklwnwwaqllphdv	19,155
37	nwknwnwwegalrmhl	42,83	11,98	nwknwawwaeklrhfl	19,14
38	nwknwlwwagqlrhfy	42,44	11,87	kwanwemwagalhmwl	19,08
39	dwwnwnwwasalrkfn	41,65	11,65	dwknwnmwqqalkkfl	18,86
40	nwknwawwcqalkmfv	41,64	11,65	nlknwnwwaqalkkfl	18,82
41	twwnwnwwaqalrkfq	39,90	11,16	nwknwawwakalkkfl	18,74
42	tnkdwnwwhqllshfw	39,69	11,10	nwknwswwaqalkwfy	18,67
43	nwknyewwagalrmfl	39,67	11,10	nwktwewwaqalkkfl	17,935
44	vwknwnwwvgalrmal	39,36	11,01	nwknwawwlgqlrkwl	17,675
45	nwknwnwwagalltfl	38,46	10,76	qwknwnwwanaltqhs	17,49
46	pwknwnwwaealamnl	38,27	10,70	nwknwnwwanaltqhv	17,19
47	nwknwcwwdgqlrkwl	37,93	10,61	dwktwnwwtqaykcfl	17,1
48	qvknwnwwdgalrmwl	37,04	10,36	dcktwnwwwnalrmfn	16,91
49	tnkdwnwwhqllrpfl	36,73	10,27	nwknwkwwlqalrhfl	16,745
50	nwknwnwwhqlllhfv	36,71	10,27	dwknmnwwagalrnwl	16,705
51	wqwnsawwaqalkkfl	36,56	10,22	dykdwncwaqllshdv	16,675
52	tmhnwnwwagalrmfl	35,83	10,02	nwknwawwaqalrmcy	16,63
53	tnqdwnwwagalrmwl	35,58	9,95	ddknwnwwaealrmfh	16,585
54	nwknwewwaealtyfl	35,08	9,81	nwmtwnwwsqalkkfl	16,525
55	tmpkwqwwaallssfl	34,72	9,71	nwknwlswatalkkfl	16,345
56	nwknnewwagalrmfl	34,23	9,57	nwknwsdwaqalrmml	16,23
57	twcnwnwwanalrmfn	34,18	9,56	nwknwnwwagalrmfn	16,19

58	nwknwlwwagqlsktl	34,13	9,54	nnknwnwwlnalnmfn	15,99
59	tvknwawwaqalkkyl	34,09	9,53	nwknwawwaqslkkfl	15,815
60	nwknwwwwalalrqfl	33,82	9,46	nwknwswwlqalrhfl	15,685
61	nwknwlwwavaltmwl	33,66	9,41	nwvnwnwwagalkkhn	15,66
62	nsnnwnhwlqalrhfl	33,56	9,39	kwknwnwwanlllhfw	15,52
63	nwknwnwvwgalrkwn	33,55	9,38	nwknwnwwanalrmcd	15,51
64	nwtnwywwaqalrmfl	33,25	9,30	dwkdwnwyaqalrkfl	15,465
65	nwknwtwwawalrwal	33,12	9,26	dwknwntwanalrvpn	15,2
66	tykdwnwwhqllshfv	32,56	9,11	dwktwnwwahalckan	15,195
67	qakkwmwwagalemfl	32,33	9,04	dkkdqnwwaqllshyv	15,195
68	nwknwnwwagalrvfl	32,00	8,95	nwknwnswaqalkkfn	15,125
69	mwkdwvwwygllrqfy	31,86	8,91	nwknwsvwaqalnhfl	14,995
70	wkkywwwwllalrhfl	31,75	8,88	nwknwnwwanalrvvn	14,94
71	dwkwnnwwanalrhwl	30,48	8,52	nwknwewwagalrmfl	14,915
72	pnkywqwwaqalkkfl	29,89	8,36	nsknwaqwaealamfl	14,905
73	nwknwylwaqalrmfl	29,86	8,35	hwkkwawwaqallhfl	14,875
74	dnktwwwwlcalrhfl	29,82	8,34	dwkawawsaqalckfn	14,565
75	pwknlnwwagalamfv	29,77	8,33	nnkmwnwwagalthfl	14,485
76	nwkcwawwalawtqfl	29,44	8,23	ewenwawwqqalkkfl	14,43
77	dpkywqwwagllssfl	29,07	8,13	nwknwnwwagalhmfl	14,355
78	pwwnwnwwagalrmvl	28,77	8,05	nwknwnwwaklylhfl	14,345
79	nwknwywwagqlqktl	28,23	7,90	nwknwewwagylrhfl	14,16
80	nmenwnwwlqaltqfl	27,63	7,73	nwknwnwwanalrmca	14,15
81	nykkwqwwaglllhsa	27,56	7,71	dwknwvvwagalrmll	14,06
82	vwknwacwagalrmwl	27,02	7,56	dwknwpwwaklllhwl	13,95
83	nwhtwwwwllalrhfl	26,88	7,52	hwknwnwwanalrhfl	13,905
84	nwqnhewwaglldhfl	26,70	7,47	nwknwawwaealrmfl	13,84
85	nwknwvwwagalrlal	26,26	7,34	nwknwkwwagalrvfn	13,78
86	nwknwywwllaerhfl	26,19	7,32	wcwdwnwwaqklrmfn	13,775
87	vwsnwnwwagllssfl	26,12	7,30	nwknwhwwacalrkfl	13,7
88	nwknwvwwygslmmwl	25,88	7,24	nwknwewwagalrmwl	13,56
89	nwknwnwvagalamsl	25,70	7,19	lwktwnewaqalrmkn	13,555
90	pnkhwqwwcgwlrmfl	25,50	7,13	dwkywetwagalrmwl	13,515
91	qwkpwnwwaqalrkfl	25,01	6,99	cwsnwewwlgqlrkwl	13,5
92	qakywmwwagalrmfl	24,75	6,92	nwknwnwwlgttrkwl	13,465
93	wqwnwawwdqalkksq	24,73	6,92	nwkhwnwwaqllshkv	13,255
94	nwknwywwagtlrmwl	24,58	6,87	dwknyewwagclrkfl	13,19
95	pnchwvnwlgllrqfy	24,57	6,87	nwknwnwwaqelrmfl	13,15
96	nwknwywwagalrhfs	24,54	6,86	nwknwecwagalrkfe	13,07
97	pwknwnwwagalrmvc	24,43	6,83	wkkvwnwwagwltqvl	13,045
98	wmkhwqawagwlhmwl	23,72	6,63	dwkkwncwagalkkfl	12,97
99	nwkewnwvwgalrmdl	23,38	6,54	nwkncnwwaealrmfn	12,85
100	nwknwnvllqalrhfl	22,95	6,42	nwkqwnwwaklllhfl	12,72
101	tnddwnwwvkalrmfl	22,73	6,36	nwknwnwwagalraws	12,69
102	wdktwwwwldslrmwl	22,52	6,30	nwknsewwagalrkfl	12,645
103	tmsnwnwwacalrmfn	22,51	6,30	nwhnwnwwagwlksfl	12,47
104	qykpwqwwaglllhfl	22,33	6,25	nqknwnwwagllyhfl	12,43
105	vwkvwnwwanalrmfn	22,10	6,18	nwknwawwacalrmfn	12,42
106	wqwwlnwkngalrmwl	22,00	6,15	nwknweewagalrmwl	12,375
107	wkwtwwwwagalrmwl	21,95	6,14	nwkntnwwanalrtfn	12,375
108	npkcwnwvwgalrhfl	21,49	6,01	nwknwnwwalkllhfl	12,365
109	nwkncewwagallhwl	21,37	5,98	nwknwawwcealrmfl	12,35
110	pnknwawwaqwakkkl	21,36	5,97	kwpnwnwwagalrmfn	12,295
111	wqwwnnwaagalrhfl	21,04	5,88	nwknwewwagalchwy	12,235
112	vwenwnwwagalrmfl	20,86	5,83	dwkncqwwaglltqtl	12,185
113	qylkwqwwagalrmwl	20,73	5,80	nwkkwykwadalvmcp	12,15
114	tmsnwncwagalrhfl	20,44	5,72	nwknwywwakllchfl	12,075
115	nwknwnwwhgalrmsl	20,40	5,70	nwknwewwagvlrcfn	12,05
116	wnsswnwwagqlrlfc	20,16	5,64	nwknwwwwlgqlrnfl	11,975
117	wkktwpwwllalrhfl	20,13	5,63	nwknwawlaqalskfl	11,93
118	wqwwlwtfdaklrhfl	20,09	5,62	nwkwwawwakalkasl	11,87
119	nwknwnwwagaytqfv	20,03	5,60	nwknwawwnqalkkel	11,555
120	wdktkwwwnlalrmwl	19,74	5,52	mwwnwykwacalrvfn	11,525
121	wqwwwnwaagysrmwl	19,18	5,36	nwykwswwaqalrmfl	11,43
122	wcwwwnwwannlrwfn	19,05	5,33	nwknwewwanaarhfn	11,24
123	twknwnwwdgawrhhl	18,76	5,25	dwenwnwwanalrmfc	11,2
124	nwknwnkwdaaltqfl	18,58	5,20	dwknwnwvasalrvfn	11,2
125	nwknwvwyygalrhfl	18,45	5,16	nwknnnwwaealrmfq	11,025
126	wqwwwqwwlqamrhfl	18,06	5,05	awmnwnwwanalwqhm	10,925
127	nwhnwvwwagaltqfl	17,97	5,03	dwknwpwwanalrvfn	10,885
128	nakkwmwvanllshwl	17,91	5,01	nwknwawwaealcmfn	10,835
129	dwwnwnwwavalrsdn	17,83	4,99	dwktwnwwaqaqkkfl	10,83
130	qykknqwwaglllhfl	17,50	4,89	dwtnwnwwagcllhfl	10,825
131	qykkwqwwaglllkkl	17,06	4,77	dwknwnpwanalrsfl	10,365
132	nhkkwqwwagtllhfl	16,82	4,70	nkknwswwaqalrmfl	10,345
133	pwkawnwwvaklrphl	16,79	4,70	nwknmnwwagqlrhwl	10,315
134	wkktwwhwllalrmnl	16,60	4,64	nwknwnwwanaqrkfl	10,2
135	ntknwnwlagaltqfl	16,38	4,58	nhknwnwwagalkcfl	10,12
136	nwknanwwagaltqfq	16,31	4,56	nwqnwnwwanalrvfn	10,095
137	tvqkwqwwagslshwl	16,24	4,54	dwknwnwwpnalrmcd	10,095
138	nwenwnwwlqdlrmwl	16,21	4,53	nqknwewwcgalrmll	10,025
139	nwknwnwwmgqlsktl	16,05	4,49	nwknwnwwdgalrhwl	10,02
140	wnsswnwwayalelfl	15,72	4,40	nwktwnwdaqalkkfl	9,8
141	nwltwwwwllaerhfc	15,57	4,35	ncknwewwagalrcwl	9,78
142	dwwnwnwwaeanrmfl	15,26	4,27	dwkywewwtgalrmwl	9,77
143	nwknwvwyygllrqfy	15,24	4,26	nwknwswwaqamrmfl	9,635
144	nwkyweswagalrqfd	14,98	4,19	nwkvwndwaqllshdv	9,53
145	qakkwmwwagalrafn	14,54	4,07	nwknwewyagalrkvd	9,485
146	wqwwvnwwawaltqfl	14,49	4,05	nakmwdqwagclkkfl	9,375
147	nwknwnwqwgalrhal	14,20	3,97	nwknwnwwawahrmfl	9,335
148	pwknwpwwagallmsl	14,14	3,95	awmyvnwwqqalrlfl	9,205
149	nwwywnwwagalrmal	13,94	3,90	ewknwnwwapqlrhwl	8,995
150	wkatwwwqanhlrmfn	13,28	3,71	vwknwnwlanalrmfn	8,965
151	nkknnnwvwgalrhfl	12,82	3,58	nvynwnwwagalrlfl	8,915
152	wkktwwwlvlamrhfs	12,69	3,55	nwktwnwwaqaeckfl	8,87
153	hwkhwqwyagwvrmwl	12,64	3,54	ncknwnwwanplkafl	8,85

154	nwynwnwwagatamsl	12,51	3,50	nwsnwnwwngalrmwc	8,665
155	twknwnwwdgaltcwy	12,09	3,38	nwknwkdwedahdmwl	8,64
156	mwknwnvwagalrqfn	11,97	3,35	nwknwnwwwnahtqhl	8,52
157	tcqkwqwwagqlsntl	11,93	3,34	nwknwnwcagylrwwl	8,34
158	wcknwnwvwgamrhdl	11,67	3,26	dwhnpnwwqqaykkfl	8,235
159	tvknwnwwagplrmfl	11,43	3,20	nqknwlwwaeyyrkfn	8,21
160	tnkdwnwyaqalrmfl	11,11	3,11	dwknwnwsamalckfn	7,95
161	tnkdwnwwagalrmks	10,69	2,99	dwkhwnwwdgnlrkwl	7,93
162	cnkdwnwwhgalrhtl	10,19	2,85	nwknkywwaealrmfl	7,87
163	nwknwewwagearmfv	10,17	2,84	nwknnswwaqayrmfn	7,8
164	wnsswnvwagaltqfl	10,08	2,82	nwkneawwaealrmwl	7,735
165	nwknwnwsasaltqfl	8,88	2,48	nwknvewwaqelrmfl	7,64
166	pnkhwawwaqplkkfl	8,86	2,48	wqknwewwsgalrmnl	7,47
167	nwknhnavwgalrhwl	8,83	2,47	nwcnwcwwagaltqfl	7,38
168	wkktwwyfvaklrpel	8,77	2,45	nwknwaweaqalckfs	7,315
169	wqwnwnwwdgaprmwl	8,74	2,44	dwkqwnwwpnalrmmn	7,3
170	qakkwmwwadlhrmfl	8,09	2,26	nwkkwnwwanacrvfl	7,295
171	nwkcwnwaagalemfv	7,29	2,04	nwevwawwqvalkkfl	7,26
172	nwknkywwaqtlrmfl	7,25	2,03	wwenwnwwaglllcfl	7,015
173	wwknwvwnynllrqmy	7,02	1,96	nwknwnwhansllvfn	6,995
174	twknwnwaagalrmfv	6,59	1,84	wwknknwwanalymfn	6,99
175	tvqkwqwwalacrkyl	6,38	1,78	dwknwnwwagaprmfl	6,905
176	nwknwewmahalrmfl	6,00	1,68	nwkawnwwanaprmfn	6,525
177	nwknwawwmqamkkfy	5,43	1,52	nwknhnewagalkkfl	6,385
178	qaklpnwwsgelrmwl	4,99	1,40	nwnnmeqwagalrmwl	6,25
179	qykywqweagalrmwl	4,80	1,34	nwknenwwecqlthwl	6,145
180	nqknplwwagaltqfv	4,73	1,32	nwkndaswaealrmfl	6,14
181	qykkwqwwagdlrtfp	3,92	1,10	dwwvwnwwagqlrmfl	6,12
182	naknwewdagalrmwl	3,66	1,02	dwknwnwwanapymfl	6,105
183	nwknvnwvwgaprmwl	3,50	0,98	dwknwnwpygqlrhwy	5,555
184	ansswnwwagqmrkwl	3,26	0,91	nlknqnwwaqelymfl	5,34
185	nwknwewmagalrmwn	2,84	0,79	wkkdwnwyaqlcstfl	5,25
186	nwklvlwnagsesmtl	2,77	0,77	wkkdwnwwagasrmll	5,105
187	nemhwewwtgatvmwl	2,72	0,76	nwknqewwagylyvwl	4,905
188	twknwnywqgactqaq	2,51	0,70	dwknwcwkanalrmcd	4,86
189	tyknelwwagqlsktl	2,34	0,65	nwnneewwagalrmwl	4,83
190	qykkwqwdagllrphl	2,18	0,61	nwknwndkaqalkkfl	4,265
191	lssswclwegekrmwl	1,72	0,48	pwknwnwwanplrvln	4,145
192	qakkwmwnagllsmfn	1,70	0,47	awknwawhayalckfn	3,99
193	nwmnvnwwpgdlactl	1,31	0,37	nmenwawmsqqlrhwl	3,91
194	tmsnwnwvagmlaksn	1,28	0,36	wqknwdwwegalrmdv	3,375
195	hkkwwnwaanaermfn	1,01	0,28	dwktpkwwqqwpkkfl	3,015
196	wnsswnkwagakrmwl	0,80	0,22	dyknwewwagaermll	2,005
197	qnkdwnwwhqhqshfv	0,66	0,18	awvnwnwsqqaprmvl	1,815
198	nwknlnwnngalrmvy	-0,03	-0,01	awmntnwqaqalrkfl	1,5
199	kvenwnqwdnactmtn	-0,32	-0,09	dwktknwwaqaprmcd	1,125
200	nwknpnwvtgqlsktl	-0,69	-0,19	dwknenwwaqaeckfn	0,58
	**Lead from Gen8**	**x**	**c**	**Lead from Gen9**	**x**
	nwknwewwagalrmwl	94,13	26,33	dwknwnwwanalrmfn	33,02
	nwknwnwwagaltqfl	58,44	16,35	dwktwnwwaqalkkfl	34,85
	nwknwawwaqalkkfl	79,60	22,26	nwknwewwagalrmwl	14,81
	dwknwnwwanalrmfn	97,48	27,27	dwknwnwwanalrmcd	28,91
	nwknwnwvwgalrhfl	43,31	12,11	dwknwnwwanalrvfn	30,15
	nwknwnwwagalrmfl	60,17	16,83	nwknwewwagalrkfl	18,46
	nwknwnwwdgalrmwl	44,71	12,51	nwknwawwaqalkkfl	18,38
	nwknwlwwagqlsktl	41,71	11,67	nwknwnwwlgqlrkwl	15,55
	nwknwywwaqalrmfl	39,43	11,03	nwknwawwaealrmfl	14,81
	wkktwwwwllalrhfl	38,16	10,67	dwknwnwwagqlrhwl	22,10
	wnsswnwwagqlrkwl	48,29	13,51	nwknwewwagylrmwl	13,58
	tnkdwnwwaqalrkfl	50,43	14,11	nwknwnwwaklllhfl	15,03
	qykkwqwwaglllhfl	35,09	9,81	nwknwnwwagalrmfl	14,92
	nwknwvwwygllrqfy	33,67	9,42	dwknwswwlqalrhfl	29,59
	pnkhwqwwagwlrmwl	36,27	10,14	nwknwawwaqalckfn	16,74
	tmsnwnwwakalrmfl	37,65	10,53	nwknwnwwagalkkfl	17,50
	wqwwwnwaagalrmfv	21,30	5,96	wkkdwnwwaqllshdv	18,82
	dwwnwnwwagalrmal	32,28	9,03	nwknwnwwanaltqhl	14,67
	tnkdwnwwhqllshfv	30,14	8,43	awmnwnwwaqalrkfl	22,56
	pwknwnwwagalamsl	37,41	10,46	nwknwnwwaqelrmfl	13,78
	tvqkwqwwagllssfl	29,28	8,19	nwknwswwaqalrmfl	18,43
	qakkwmwwagllshwl	33,11	9,26	nwknwnwwagaltqfl	14,41
	wqwwlnwfngalrmfl	28,90	8,08	nwenwawwqqalkkfl	14,53
